# Host Immunity Influences the Composition of Murine Gut Microbiota

**DOI:** 10.3389/fimmu.2022.828016

**Published:** 2022-03-15

**Authors:** Vincent Van averbeke, Matilda Berkell, Mohamed Mysara, Juan Pablo Rodriguez-Ruiz, Basil Britto Xavier, Fien H. R. De Winter, Bart ‘s Jongers, Ravi Kumar Jairam, An Hotterbeekx, Herman Goossens, E. Suzanne Cohen, Surbhi Malhotra-Kumar, Samir Kumar-Singh

**Affiliations:** ^1^ Molecular Pathology Group, Laboratory of Cell Biology and Histology, University of Antwerp, Antwerp, Belgium; ^2^ Laboratory of Medical Microbiology - Vaccine and Infectious Disease Institute, University of Antwerp, Antwerp, Belgium; ^3^ Microbiology Unit, Belgian Nuclear Research Centre (SCK-CEN), Mol, Belgium; ^4^ Bioscience Asthma, Research and Early Development, Respiratory and Immunology, BioPharmaceuticals R&D, AstraZeneca, Cambridge, United Kingdom; ^5^ Translational Neurosciences, University of Antwerp, Antwerp, Belgium

**Keywords:** Th1-Th2 balance, BALB/c, C57BL/6, IL-4Rα knockout, IL-33 knockout, NSG, cytokines/chemokines, T-cell analysis

## Abstract

The influence of gut microbiota on host immunity is widely studied, and its disturbance has been linked to several immune-mediated disorders. Conversely, whether and how inherently disturbed canonical Th1 (pro-inflammatory) and/or Th2 (anti-inflammatory) immune pathways modify the host microbiome is not sufficiently investigated. Here, we characterized the humoral, cellular, and cytokine immunity, and associated alterations in gut microbiota of naïve wild-type mice (C57BL/6 and BALB/c), and mice with deficiencies in Th2 responses (IL-4Rα and IL-33 knockout mice) or in both Th1 and Th2 responses (NOD *scid* gamma, NSG mice). A global analysis by *de novo* clustering of 16S rRNA profiles of the gut microbiota independently grouped wild-type immunocompetent (C57BL/6 and BALB/c), Th2-deficient (IL-4Rα^-/-^ and IL-33^-/-^), and severely immunodeficient (NSG) mice; where wild-type mice, but not Th2 or severely immunodeficient mice, were enriched in gut bacteria that produce short-chain fatty acids. These include members of phyla Firmicutes, Verrucomicrobia, and Bacteroidetes such as *Lactobacillus* spp., *Akkermansia muciniphila*, and *Odoribacter* spp. Further comparison of the two naïve wild-type mouse strains showed higher microbial diversity (Shannon), primarily linked to higher richness (Chao1), as well as a distinct difference in microbial composition (weighted UniFrac) in BALB/c mice compared to C57BL/6. T-cell and blood cytokine analyses demonstrated a Th1-polarization in naïve adaptive immunity in C57BL/6 animals compared to BALB/c mice, and an expected Th2 deficient cellular response in IL-4Rα^-/-^ and IL-33^-/-^ mice compared to its genetic background BALB/c strain. Together, these data suggest that alterations in the Th1/Th2 balance or a complete ablation of Th1/Th2 responses can lead to major alterations in gut microbiota composition and function. Given the similarities between the human and mouse immune systems and gut microbiota, our finding that immune status is a strong driver of gut microbiota composition has important consequences for human immunodeficiency studies.

## Introduction

The mammalian gastrointestinal tract harbors a large community of microorganisms consisting of bacteria, viruses, and fungi that play an important role in digestion, metabolism, and immunity in the host (1, 2). The intestinal commensal microbiota of healthy adults is shown to be primarily composed of Bacteroidetes, Firmicutes, Actinobacteria, Proteobacteria, and Verrucomicrobia of which the first two phyla are predominant (3, 4). This microbial community reportedly interacts in health and disease with gut tissue, brain, lungs and other organs mostly by producing essential metabolites such as vitamins, indoles, bacteriocins, and short-chain fatty acids (SCFAs), but also by direct transfer of gut microbiota to respiratory tract especially in immuno-compromised conditions (1, 5–9). SCFAs are dietary fiber-derived metabolites and are mainly produced by *Ruminococcaceae* (e.g., *Faecalibacterium prausnitzii*) and *Lachnospiraceae* (e.g., *Roseburia* and *Anaerostipes* spp.) that belong to the phylum Firmicutes (1, 5). SCFAs have been proposed to strengthen the intestinal epithelial layer and influence local and systemic immune responses by modulating levels of cytokines such as the anti-inflammatory interleukin (IL)-10, and by inhibiting the expression of colonic pro-inflammatory cytokines such as tumor necrosis factor (TNF)-α, interferon (IFN)-γ, IL-1β, and IL-6 (5, 8).

Pro-inflammatory cytokines are secreted chiefly by T helper type 1 (Th1) cells and promote inflammation necessary for clearance of pathogens and for perpetuating autoimmune responses in the gut and other tissue (10–12). The other group of cytokines comprising IL-4, IL-5, and IL-13, amongst others, are secreted chiefly by Th2 cells and are essentially anti-inflammatory, but are also involved in several allergic responses (10–12). Other types of Th cells also exist, notably Th17 cells that secrete pro-inflammatory cytokines belonging to the IL-17 family (12, 13). There is growing evidence that nutrients that fuel metabolic processes impact on the ways immune cells, in particular, macrophages, respond to different stimuli under physiological and pathological conditions (14).

Interestingly, the human and murine immune systems show remarkable similarity with more than 70% conservation observed in gene expression patterns (15). Thus, immune responses to infection or other interventions are routinely studied in common laboratory wild-type mouse strains such as C57BL/6 and BALB/c [reviewed in (16)]. Several studies have also shown that C57BL/6 animals, when challenged with infectious agents or foreign exogenous immunogenic material, show a Th1-polarized immune response compared to wild-type BALB/c (17–21). To our knowledge, no study has investigated cellular and cytokine immunity in naïve C57BL/6 and BALB/c, which could be an important consideration for immune response studies and for pre-clinical trials on immunological diseases.

Additionally, as the dominant bacterial phyla in the rodent and human gut are grossly similar (22), rodent models also allow the study of gut microbiota dynamics in a more controlled setting that is not possible in humans. Several studies in mice have shown a link between immunodeficiency and altered microbial composition where certain taxa modulate the animal’s sensitivity to colonization and infection with enteropathogens (23–26). However, studies aiming to characterize the cellular and cytokine/chemokine responses in relation to gut microbial composition particularly in absence of any immune stimulation, remain scarce (24–27).

Here, we characterized the humoral, cellular, and cytokine immunity and associated alterations in gut microbiota in naïve wild-type mice (C57BL/6 and BALB/c), in mice with deficiencies in Th2 responses (IL-4Rα^-/-^ and IL-33^-/-^ mice), and in severely immunocompromised mice with a deficiency in both Th1 and Th2 responses (NOD *scid* gamma, NSG mice). We show that the gut microbial composition is highly immunocompetence-dependent where immunocompromised animals demonstrated reduced or depleted levels of probiotic bacteria producing metabolites essential for gut homeostasis.

## Materials and Methods

### Animals and Housing

Adult mixed-gender mice aged 3–6 months included C57BL/6NRj (n = 18; Janvier Laboratories), BALB/cJR (n = 28; Janvier Laboratories), IL-4Rα^-/-^ BALB/c (n = 28; BALB/c-IL-4Rα^tm1Sz^/J, Jackson Laboratory/JAX), and IL-33^-/-^ BALB/c (n = 21; AstraZeneca). A proportionately successive age category between 3–6 months, considered as adults (28), was chosen to mitigate any potential confounding effect of ageing in our immunodeficient mouse strains. This is because immunodeficiency causes differential ageing (29–31), and ageing is strongly linked to an altered gut microbiota profile (32–36). As no effect of ageing is known for NOD *scid* gamma mice (n = 26 mice; NSG, NOD.Cg-*Prkdc^scid^ Il2rg^tm1Wjl^*/SzJ, JAX) that is one of the most immunocompromised mouse strains known so far, we studied two age groups of 3–6 months (n = 19) and of 10 months (n = 7). A highly significant difference was observed between NSG 3–6 months and the purposefully chosen 10 months NSG mice (*P* < 0.001; pairwise comparisons with Bonferroni correction), but not between any of the 3–6 months wild type and immuno-compromised mouse strains. All animals were housed in individually ventilated cages (IVCs, up to 5 mice per cage) under specific pathogen free (SPF) conditions. C57BL/6NRj and BALB/c/cJR (hereafter referred to as C57BL/6 and BALB/c, respectively) mice were obtained directly from Janvier Laboratories and were allowed to acclimatize for 4 weeks. All other mice were acquired previously and were maintained through sibling breeding colonies. Autoclaved water and chow (Ssniff, Germany) were provided *ad libitum*, and cages were changed weekly.

### Sample Collection

Animals were anaesthetized with isoflurane (2.5%) and euthanized by exsanguination while collecting cardiac blood in 1.5 mL microcentrifuge tubes containing 20 µL EDTA. The cecum was promptly harvested and contents removed by squeezing it in sterile tubes under aerobic conditions, as described previously (37), followed by storage at –80°C until further use. In a pilot experiment, caecal contents were harvested under both aerobic and anaerobic conditions, the latter utilizing an anaerobic chamber (Bugbox, Baker Ruskinn, UK), and did not show any difference on 16S rRNA gene analysis (data not shown).

### Immune Staining and Flow Cytometry

An aliquot of whole blood (100 µL) was lysed for 5 min using multi-species RBC lysis buffer as described by the vendor (Invitrogen, USA) and washed with PBS. The following antibodies (BioLegend, USA) were used for staining: BV421 hamster anti-mouse CCR6, APC hamster anti-mouse CXCR3 (1:10), PE hamster anti-mouse CCR4, PerCP/Cy5.5 rat anti-mouse CD4 (1:5), BV510 rat anti-mouse CD45 (1:5), and FITC hamster anti-mouse CD3ϵ (1:5). Non-specific antibody binding was blocked by a 10 min incubation with mouse FcR blocking Reagent (Miltenyi Biotec, The Netherlands), followed by an immediate incubation with the antibody cocktail (5 µL per antibody) for 15 min, and cell fixation with 4% paraformaldehyde in PBS for 15 min. Cells were washed with PBS and resuspended in 200 µL staining buffer for storage at 4°C until further use. Washing steps were performed between all incubation steps using cell staining buffer [PBS with 1% bovine serum albumin (BSA), and 0.1% sodium azide (NaN_3_)] unless stated otherwise.

Samples were analyzed on a BD FACSAria II system (BD Biosciences, US), and analysis was performed in FlowJo v10.8.1. Total white blood cells were isolated by CD45^+^ gating. Within the CD45^+^ population, CD3^+^/CD4^+^ cells were selected. To determine relative abundance of T helper cell subsets, CD4^+^ populations were gated on CXCR3, CCR4, and CCR6 markers. Th1 cells were selected as CXCR3^+^/CCR4^-^, Th2 cells as CCR4^+^/CCR6^-^, and Th17 cells as CCR6^+^/CCR4^-^.

### Cytokine Analysis

Plasma was collected by spinning down leftover EDTA-blood at 3,000 ×g for 15 min. Collected plasma was utilized to measure cytokine levels using MSD mouse U-plex assays containing antibodies for IL-1β, IL-2, IL-4, IL-5, IL-6, IL-10, IL-12 (p70), MIP-2, MCP-1, IP-10, IFN-γ, TNF-α, KC-GRO, IL-13, IL-17A, IL-17F, IL-22, IL-23, IL-33, and GM-CSF. These assays were performed using a MESO QuickPlex SQ 120 system (Mesoscale Discovery; MSD, Rockville, MD, USA) followed by data analysis and imaging in R v4.0.2.

### DNA Extraction, Library Preparation, and Sequencing

Total bacterial DNA was extracted from collected caecal samples using the MP FastDNA Spin Kit for Feces (MP Biomedicals, USA) according to the manufacturer’s instructions and quantified with the Qubit dsDNA HS Assay Kit with a Qubit 3.0 Fluorometer (ThermoFisher Scientific, USA). The average DNA concentration (± standard deviation, SD) of all animals in this study was 332 ± 134.0 ng/µL. PCR amplification of the V3–V4 regions of the 16S rRNA gene was performed using Illumina fusion primers (341F and 802R) with 2x KAPA HiFi Hot Start Ready Mix (Roche, Switzerland) with the following settings: initial denaturation at 95°C for 3 min; 25 cycles of 95°C for 30 sec, 60°C for 30 sec and 72°C for 30 sec; elongation at 72°C for 10 min in triplicates of each sample. Multiplexed 16S rRNA gene libraries were prepared using the Nextera XT kit (Illumina Inc., USA) followed by 2 x 250 paired-end sequencing performed with V2 chemistry on a MiSeq instrument (Illumina Inc., USA).

### Primary Data Analysis

Raw read quality was assessed using FastQC followed by primary analysis using the OCToPUS pipeline v1.0 (38). Briefly, raw reads were denoised based on k-mer frequency implemented in SPAdes v3.5.0 (39) followed by heuristically merging denoised paired end reads into contigs based on Phred score in mothur v.1.39 (40). Contigs were aligned to the SILVA database v.132 (41), followed by trimming of contigs with ambiguous bases, more than 8 homopolymers, a length below 390 bp, and reads not aligned to the V3–V4 regions of the 16S rRNA gene. De-replication and removal of sequencing errors were conducted using IPED v1.0 (42), followed by chimera removal by utilizing the *de novo* mode of CATCh v1.0 (43), and clustering of sequences into operational taxonomical units (OTUs) at 97% identity using UPARSE (USEARCH v8.1.186 implementation) (44). Samples were rarefied to the minimum sequencing depth of 26,453 processed reads (range: 26,453 – 32,261) to avoid sample size dependencies in assessing diversity (**Supplementary Figure 1A**). Finally, taxonomic classification was conducted utilizing the RDP database v.18 (45). Data reproducibility was assessed by incorporating positive controls consisting of well-characterized mock communities containing quantified proportions of DNA from 20 species (HM-783D, BEI Resources, USA) as well as biological sample repeats between batches (**Supplementary Figures 1B, C**). Each sequencing batch further included negative control samples consisting of negative PCR controls.

### Statistical Analysis

Plasma cytokine levels were stratified by genotype and tested for homogeneity of variance using Levene’s test. Statistical comparisons of cytokine levels were conducted using ANOVA or Kruskal-Wallis tests as indicated, followed by Tukey HSD or Dunn *P*-value correction, respectively. Group differences in cytokine, chemokine, and growth factor (CCG) profiles were explored by Partial Least Squares Discriminant Analysis (PLS-DA) in Metaboanalyst (https://www.metaboanalyst.ca/). Missing data was fixed to 1/5 of the lowest detected value followed by data normalization using autoscaling and natural log transformation. A-diversity expressed as Shannon diversity and Chao1 richness indices, and β-diversity described by weighted UniFrac were calculated in mothur. Statistical comparisons of α-diversity indices were conducted in R v.4.0.2 using two-sided non-parametric Kruskal-Wallis (KW) and Mann-Whitney (MW) tests as indicated, followed by Bonferroni *P*-value correction. β-diversity was compared using analysis of molecular variances (AMOVA) and analysis of similarities (ANOSIM) in mothur. Differences in sample composition were captured by principal coordinate analysis (PCoA). Differentially abundant OTUs (LDA ≥ 3.0) were identified using linear discriminant analysis effect size (LEfSe) v1.0.0 (46), and *de novo* clustering of samples was conducted using the Dirichlet multinomial mixtures (DMM) clustering module (47) in mothur. When comparing BALB/c and C57BL/6 animals, Pearson correlations between CCG data and differentially abundant OTUs identified by LEfSe were done using the *otu.association* command in mothur. Missing CCG data was set to 1/5 of the lowest detected value. Functional analysis was conducted using PICRUSt2 (48, 49) v.2.1.3 with KEGG Ortholog (KOs) reactions (50) from the IMG v.4 database (51) as reference. Statistical assessment of different groups was conducted in STAMP v.2.1.3 (52) using non-parametric testing (Kruskal-Wallis) with Welch’s *post-hoc* test and Benjamini-Hochberg FDR correction of *P*-values. Only results with an effect size ≥ 0.5 are reported. For all statistical assessments in this study, a corrected *P*-value < 0.05 was considered significant. While all comparisons were taken into consideration when conducting Bonferroni correction, significant differences for only the discussed groups in the manuscript are shown in **Figures 2** and **4A**.

## Results

### Naïve C57BL/6 Mice Are Th1-Leaning Compared to BALB/c Mice

**Figure 1:**
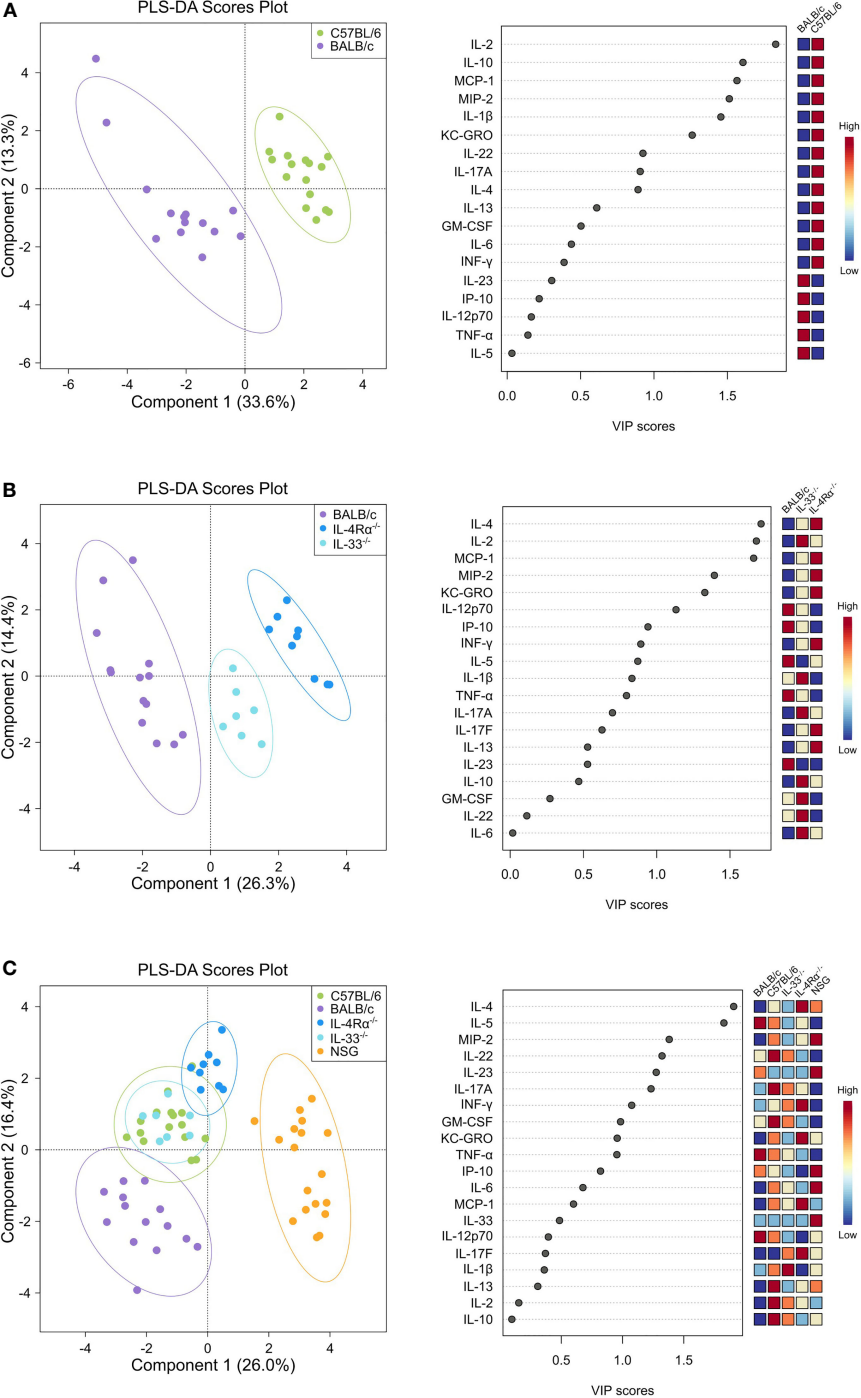
Discriminant analysis reveals distinct cytokine, chemokine, and growth factor (CCG) profiles in naïve wild-type, Th2-deficient, and Th1/Th2-deficient animals. Plasma CCG levels were measured with multiplexed assays in SPF-housed wild-type C57BL/6 (n = 16, green) and BALB/c (n = 14, purple), Th2-deficient BALB/c IL-4Rα^-/-^ (n = 9, blue) and IL-33^-/-^ (n = 7, light blue), and Th1/Th2-deficient NSG (n = 18, orange) animals and analyzed with partial least squares discriminant analysis (PLS-DA). Two-component plots on the left visualize the differences between genotypes whereas variable importance (VIP) scores are visualized with dot plots and indicate the contribution of each CCG with the contribution of individual genotypes displayed as a heatmap on the right. **(A)** C57BL/6 and BALB/c wild-type mice were compared, showing a clear separation in cytokine profiles that was mostly attributed to cytokines IL-2, IL-10, MCP-1, MIP-2, and IL-1β as indicated by the VIP scores. **(B)** Cytokine profiles of BALB/c wild-type, IL-4Rα^-/-^, and IL-33^-/-^ mice were analyzed, showing notable differences between knock-out and wild-type mice, mostly due to IL-4, IL-2, and MCP-1, which were more pronounced in IL-4Rα^-/-^ mice. **(C)** By comparing all genotypes, a clear distinction between NSG mice cytokines and all other studied genotypes was observed, where IL-4 and IL-5 constituted the major contributing factors. VIP, Variable importance in projection; CCG, cytokine chemokine and growth factors.

Differences in baseline plasma levels of pro- and anti-inflammatory cytokines, chemokines, and growth factors (CCGs) were first assessed in 3 to 6 months old, mixed-gender C57BL/6 (n = 16) and BALB/c (n = 15) mice, which are two of the most common wild-type strains utilized in preclinical research. First, we identified patterns of inflammatory mediators associated with C57BL/6 and BALB/c by partial least-squares discriminant analysis (PLS-DA), which led to an overall classification accuracy of 97.6% between the two wild-type strains (R^2^ = 0.92, Q^2^ = 0.87 for 2 components, **Figure 1A**, **Table 1**).

**Table 1:** Accuracy of discriminant models in predicting cytokine, chemokine, and growth factor (CCG) profiles across mouse genotypes.

	C57BL/6 - BALB/c			BALB/c - IL-4Rα^-/-^ - IL-33^-/-^			All genotypes		
	Accuracy	R^2^	Q^2^	Accuracy	R^2^	Q^2^	Accuracy	R^2^	Q^2^
**1 component**	0.952	0.799	0.762	0.767	0.904	0.873	0.531	0.792	0.746
**2 components**	1.0	0.924	0.872	1.0	0.960	0.912	0.750	0.879	0.820
**3 components**	1.0	0.945	0.885	1.0	0.978	0.925	0.891	0.905	0.824
**4 components**	1.0	0.953	0.876	1.0	0.982	0.917	0.859	0.924	0.812
**5 components**	1.0	0.957	0.856	1.0	0.984	0.903	0.875	0.932	0.808

Partial least squares discriminant analysis (PLS-DA) for up to 5 components is shown.

**Figure 2:**
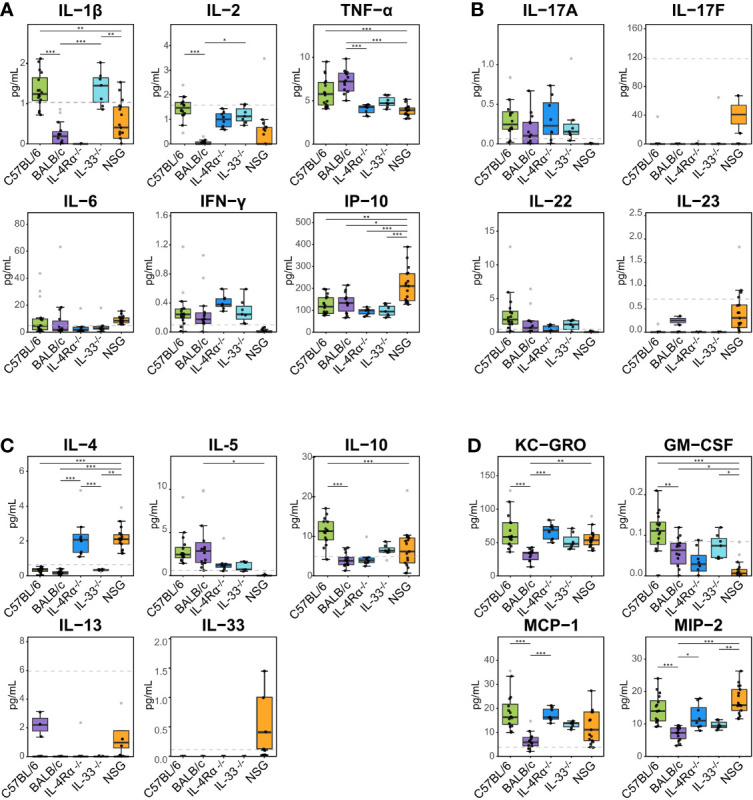
Plasma cytokine, chemokine, and growth factor (CCG) levels in naïve immunocompetent and immunodeficient mice show immune polarizations. Plasma cytokine levels were measured with multiplexed cytokine assays in SPF-housed wild-type C57BL/6 (n = 16, green) and BALB/c (n = 14, purple), Th2-deficient IL-4Rα^-/-^ (n = 9, blue) and IL-33^-/-^ (n = 7, light blue), and Th1/Th2-deficient NSG (n = 18, orange) animals. Cytokines were studied as **(A)** pro-inflammatory Th1, **(B)** pro-inflammatory Th17, **(C)** anti-inflammatory Th2, and **(D)** immunomodulatory and other growth factors. Naïve C57BL/6 animals are Th1-leaning compared to BALB/c mice due to higher levels of e.g., IL-2, IL-1β, and immunomodulatory IL-10. Similarly, naïve Th2-deficient animals are also Th1 leaning displaying higher levels of IL-2, KC-GRO, MCP-1, and MIP-2 compared to wild-type BALB/c mice. Dashed lines indicate limit of detection. Normality was assessed with Levene’s test, followed by parametric ANOVA or non-parametric Kruskal-Wallis tests as indicated. Box plots indicate median (middle line), 25th, 75th percentiles (box), and 5th and 95th percentiles (whiskers) as well as outliers (single gray dots). Only significances from main comparisons as described in the results section are shown. *P < 0.05. **P < 0.01. ***P < 0.001.

To identify the underlying inflammatory CCGs involved in discriminating between these genotypes, measured CCGs were assessed for differences of mean by ANOVA followed by pairwise comparisons with correction for multiple testing. Among pro-inflammatory Th1/Th17 cytokines, IL-2 and IL-1β, which are important mediators of the pro-inflammatory Th1 response, showed significant differences (16-fold and 4-fold higher in C57BL/6 compared to BALB/c, *P* < 0.001, **Figure 2A**). Neutrophil chemokines linked with a pro-inflammatory state were also elevated in C57BL/6 compared to BALB/c, including KC-GRO and MIP-2 (both > 2-fold elevated; *P* < 0.001, **Figure 2D**). Furthermore, other cytokines that were significantly different between C57BL/6 and BALB/c were IL-10 (immunomodulatory), GM-CSF (a colony stimulating factor), and MCP-1 (monocyte chemoattractant), where 2 to 10-fold elevation in levels were observed for C57BL/6 compared to BALB/c (*P* < 0.001 for all except GM-CSF where *P* = 0.025, **Figure 2C, D**). The VIP scores in the right panel of **Figure 1A** indicate the relative contribution of individual CCGs to the discriminant model.

**Figure 3:**
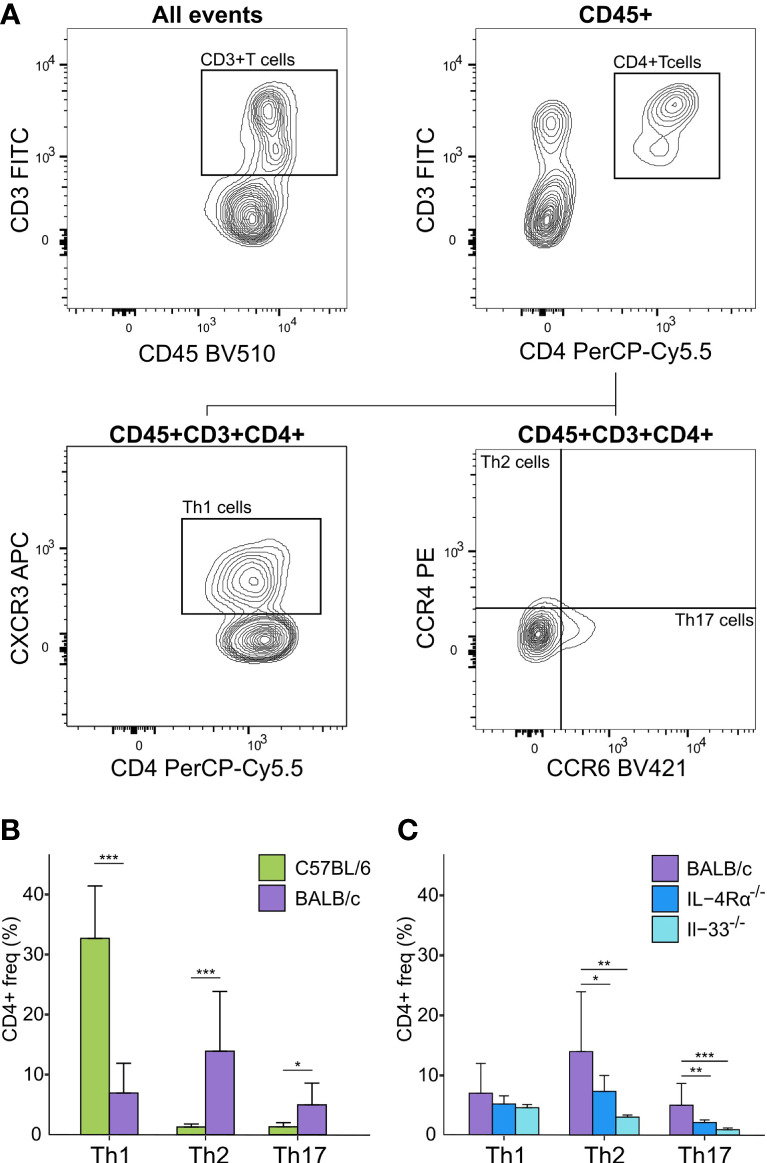
Flow cytometry analysis of Th cell subsets in blood of naïve immunocompetent and immunodeficient mice show immune polarizations. **(A)** Flow cytometry gating strategy for T-cells (CD45+/CD3+/CD4+) further distinguished as Th1 (CXCR3+/CCR4-), Th2 (CCR4+/CCR6-), and Th17 (CCR6+/CCR4-) cells. **(B)** C57BL/6 animals (green, n = 16) are Th1-polarized whereas BALB/c (purple, n = 12) are Th2 polarized, and show significantly higher Th17 cells compared to C57BL/6. **(C)** BALB/c mice also showed a Th2 polarization compared to both IL-4Rα^-/-^ (blue, n = 16) and IL-33^-/-^ (light blue, n = 7) mice that also showed significantly higher levels of Th17 cells compared to knock-out mice. No T-cells were observed for NSG mice. *P < 0.05. **P < 0.01. ***P < 0.001.

Concerning cell-based immunity, flow cytometry analysis showed significant differences between C57BL/6 and BALB/c mice in all T-cell subsets. Compared to BALB/c mice, C57BL/6 mice showed a 5-fold increase in Th1 cells (CD3+/CD4+/CXCR3^+^/CCR4^-^), but an 11-fold decrease in Th2 cells (CD3+/CD4+/CCR4^+^/CCR6^-^), and a 4-fold decrease in Th17 cells (CD3+/CD4+/CCR6^+^/CCR4^-^) (*P* < 0.05, **Figure 3A, B**). Thus, in support of earlier data where a Th1-polarized immune response for C57BL/6 and a Th2-polarized response for BALB/c mice was shown under bacterial or other exogenously challenged conditions (18–21), our data suggest that naïve C57BL/6 are already Th1-leaning compared to naive BALB/c mice in absence of any prior challenge.

### Exaggerated Th2 Deficiency in Naïve IL-33^-/-^ Mice Compared to Naïve IL-4Rα^−/−^ Mice

Next, we assessed cellular and CCG immune responses in Th2-deficient IL-4Rα^-/-^ (n = 16) and IL-33^-/-^ (n = 7) mice. Because IL-4Rα^-/-^ and IL-33^-/-^ were constructed in BALB/c genetic background, we compared these two knockouts with wild-type BALB/c mice. IL-4 is the key Th2 cytokine and is recognized by the IL-4 receptor, which is a heterodimer complex comprised of an IL-4Rα-chain and common γ-chains. IL-13, having similar properties to IL-4, also uses the IL-4R α-chain for signaling along with the ligand-specific chain, IL-13Rα1. Therefore, IL-4Rα^-/-^ mice would be defective in both IL-4 and IL-13 signaling and would, consequently, be Th2-deficient. In support of this premise, compared to BALB/c wild-type mice, we showed a highly discriminant CCG PLS-DA profile for IL-33^-/-^ and IL-4Rα^-/-^ mice. The overall classification accuracy achieved with PLS-DA was 100% between the three genotypes (R^2^ = 0.96, Q^2^ = 0.91 for 2 components; **Figure 1B**, **Table 1**).

Differences of mean analysis showed that several pro-inflammatory chemokines responsible for chemotaxis of neutrophil and monocyte-macrophages such as KC-GRO, MCP-1, and MIP-2 were significantly increased in IL-4Rα^-/-^ mice (*P* < 0.05, **Figure 2D**). However, levels of IL-1β were unmeasurable and TNF-α was significantly downregulated compared to BALB/c wild-type, which is not surprising as IL-4Rα^-/-^ mice also showed a paradoxical upregulation of IL-4 (*P* < 0.001, **Figure 2A, C**) due to lack of functional IL-4 receptors. In this regard, high levels of IL-4 are shown to block polarization of M1 macrophages and secretion of select Th1 cytokines (53–55). In contrast, IL-33^-/-^ mice showed a significant upregulation of IL-1β compared to BALB/c wild-type (*P* < 0.001; **Figure 2A**). Significantly lower Th2 cell frequencies were observed for IL-4Rα^-/-^ and IL-33^-/-^ mice that also showed significantly lower Th17 cells (*P* < 0.014, **Figure 3C**). These data suggest that naïve IL-4Rα^-/-^ and IL-33^-/-^ mice have a deficient Th2 cellular and cytokine immunity compared to wild-type BALB/c mice. This effect is more pronounced in IL-33^-/-^ mice, likely due to the paradoxical higher levels of IL-4 in IL-4Rα^-/-^ mice.

### Presence of Both Th1 and Th2 Cytokines in Severely Immunodeficient Naïve NSG Mice Despite Near Complete Absence of Innate and Adaptive Immune Cells

NOD *scid* gamma (NSG) mice have been considered as one of the most immunodeficient mouse models with completely eliminated adaptive immunity due to the Prkdc^scid^ mutation, severely reduced hematopoietic cell differentiation due to an IL-2 receptor mutation, and substantial reduction in innate immunity due to the inbred NOD mouse strain (NOD/ShiLtJ). Because of a lack of an appropriate genetic background control strain for NSG, these mice were studied in comparison with the Th2 immunodeficient and the wild-type immunocompetent mouse strains. PLS-DA analysis showed the most discriminant cytokine profile for NSG mice compared to all other genotypes with classification accuracy of 89.1% (R^2^ = 0.91, Q^2^ = 0.82 for 3 components, **Figure 1C**, **Table 1**). Also, while flow cytometry expectedly revealed no T-cells for NSG mice and certain CCGs such as GM-CSF were amongst the lowest compared to other mouse strains, surprisingly, several CCGs were distinctly higher in NSG mice (**Figure 2D**). For instance, despite the lowest IFN-γ observed for NSG mice, IFN-γ-induced protein 10 (IP-10), primarily induced by IFN-γ itself, was significantly upregulated in NSG mice compared to both wild-type and Th2-deficient mouse strains studied here (*P* < 0.044, **Figure 2A**). Similarly, within IL-17 family members, while IL-17A and IL-22 were completely absent in NSG mice, IL-17F was notably expressed along with IL-23. While IL-17 is also linked with neutrophil function (56–58), the neutrophil recruiting MIP-2 chemokine was also elevated in NSG mice, which was significantly different from BALB/c and IL-33^-/-^ mice (*P* < 0.001, **Figure 2B, D**). Lastly, similar to IL-4Rα^-/-^ mice, a notable presence of IL-4 was also observed in NSG mice which was > 10-fold elevated compared to all other mouse groups (*P* < 0.001, **Figure 2C**). These data suggest that although most of the cellular responses are stunned in NSG mice, several CCGs are highly upregulated in this most severely immunodeficient mouse model, likely due to a lack of appropriate cellular feedback mechanisms.

### *De Novo* Clustering of Caecal Microbial Composition Clearly Differentiates Wild-Type, Th2-Deficient, and Severely Immunodeficient Mice

To investigate the link between immune response profiles and microbial composition, the caecal microbiota of C57BL/6 (n = 18), BALB/c (n = 16), IL-33^-/-^ (n = 14), IL-4Rα^-/-^ (n = 12), and NSG (n = 17) animals was compared using 16S rRNA gene profiling. As the murine microbiota could be influenced by various factors inherent to animals or the environment, we first studied and showed that caecal microbial composition of the immunocompetent or immunocompromised mice groups in our study were not confounded by experimental variables such as housing cages, dates of experimental procedures, gender, or age. The effect of age, especially, has been strongly linked to an altered gut microbiota (32–36). This has been discussed and illustrated further in **Supplementary Results**, **Supplementary Figure 2**, and **Supplementary Table 1**.

**Figure 4:**
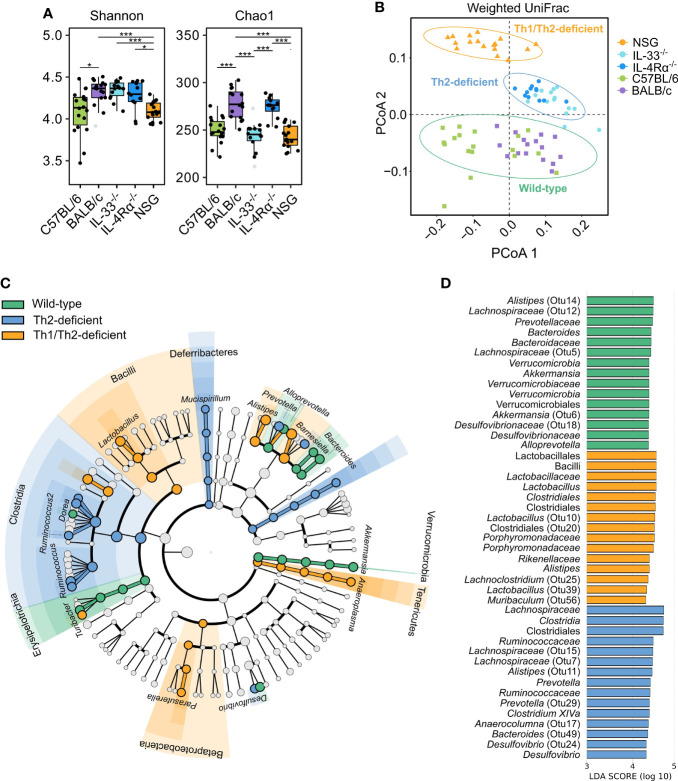
Unsupervised clustering showed three distinct microbial community types that distinguish wild-type from Th2- and Th1/Th2-deficient animals. 16S rRNA gene profiling was used to study the caecal microbiota composition in naïve, SPF-housed, wild-type C57BL/6 (green, n = 18) and BALB/c (purple, n = 16), Th2-deficient BALB/c IL-33^-/-^ (light blue, n = 14) and IL-4Rα^-/-^ (blue, n = 12), and Th1/Th2-deficient NSG (orange, n = 17) animals. **(A)** Alpha diversity described by the Shannon and Chao1 diversity indices was compared using the two-sided non-parametric Wilcoxon rank sum test followed by Bonferroni correction of *P*-values, revealing a comparable overall diversity between all BALB/c animals (WT, IL-33^-/-^ and IL-4Rα^-/-^), whereas both NSG and C57BL/6 animals showed lower diversity due to decreased richness. **(B)** Differences in microbial composition were further assessed by conducting principal coordinate analysis (PCoA) and unsupervised clustering based on weighted UniFrac distances. Three primary clusters that correlated with the animals’ immunocompetence were identified and are visualized by different shapes in the PCoA. **(C)** Differentially abundant OTUs between the three clusters were identified by linear discriminant analysis effect size (LEfSe, LDA > 3.0), which showed several taxa distinctly associated with each cluster as visualized in the cladogram. Bacterial class is shown with respective taxa at genus level. More detailed taxonomy is shown in supplement (**Supplementary Table 2**). **(D)** The LDA values of the top 15 differentially abundant taxa associated with each group are visualized. Box plots indicate median (middle line), 25th, 75th percentiles (box), and 5th and 95th percentiles (whiskers) as well as outliers (single gray dots). Only significant comparisons between groups defined by the clusters displayed in **(B)** are shown. SPF, specific pathogen free; OTU, Operational taxonomic unit; LDA, linear discriminant analysis score; IQR, interquartile range; SD, standard deviation. *P < 0.05. ***P < 0.001.

We next employed different methods of microbial diversity measurements over different groups of animals. Alpha (α) diversity, a measure of gut microbial variance within individual animals, is commonly studied by estimating overall diversity (Shannon) and richness (Chao1). While both indices identified significant differences between and across the different immunocompetent and immunodeficient animal groups (**Figure 4A**), we employed Dirichlet multinomial mixtures (DMM) analysis to find *de novo* clusters and PCoA to visualize the data. Remarkably, DMM and PCoA analysis identified three distinct microbial communities: one cluster of all collected samples from wild-type animals (C57BL/6 and BALB/c), the second from Th2-immunodeficient animals (IL-4Rα^-/-^ and IL-33^-/-^), and the third cluster from the severely-immunodeficient (NSG) animals (weighted UniFrac, AMOVA, *P* < 0.001, **Figure 4B**).

We further assessed the underlying microbial differences identified in the three *de novo* clusters of wild-type, Th2 deficient, and Th1/Th2 deficient mice by LEfSe (Linear discriminant analysis (LDA) Effect Size). Choosing a threshold of LDA > 3.0, the largest differences between groups were observed within the *Lachnospiraceae, Porphyromonadaceae*, and *Ruminococcaceae* families, and largely within the same genera (**Figures 4C, D**, **Supplementary Table 2**). Each of the three microbial communities harbored distinct levels of different members of the *Alistipes* and *Barnesiella* genera. Wild-type and Th2-deficient *de novo* clusters were further associated with elevated levels of members of the *Clostridium* XIVa and *Bacteroides* genera, whereas both wild-type and NSG-associated communities were enriched in *Lactobacillus* and *Odoribacter* spp. Additionally, unique taxa were reported to be associated with each cluster, as described in **Figure 4D** (**Supplementary Figure 3** and **Supplementary Table 2**).

We next inferred metabolic activity based on relative abundance of taxa in the three *de novo* clusters of wild-type, Th2 deficient, and Th1/Th2 deficient mice using PICRUSt (**Supplementary Table 3**). The wild-type-associated taxa were found to be more active in different kinds of biosynthesis, such as of the essential amino acid histidine, nucleotides (purine-guanosine and pyrimidines), and vitamins (biotin). The Th2-deficient cluster showed taxa associated with biosynthesis of the essential amino acid lysine as well as with adenosine and guanosine degradation, while the NSG cluster showed taxa particularly associated with increased peptidoglycan and phospholipid biosynthesis, tRNA charging, and glycolysis I/III (variants of the Embden-Meyerhof-Parnas pathway). The latter pathway, linked to SCFA production, was influenced by the differentially abundant taxa in wild-type and Th2 deficient (IL-4Rα^-/-^ and IL-33^-/-^) animals, namely *Bacteroides*, *Alistipes*, and *Akkermansia* spp. (**Supplementary Tables 2–4**). Yet, the main taxa contributing to glycolysis pathways were *Lactobacillus johnsonnii* in NSG animals, and *Desulfovibrio* spp. in both Th2-deficient and NSG animals. Together these data suggest that the immune status is strongly associated with murine gut microbiota composition and might influence production of important microbiota-derived metabolites and related functions.

### Th1- and Th2-Leaning C57BL/6 and BALB/c Are Characterized by Unique Caecal Microbial Composition

**Figure 5:**
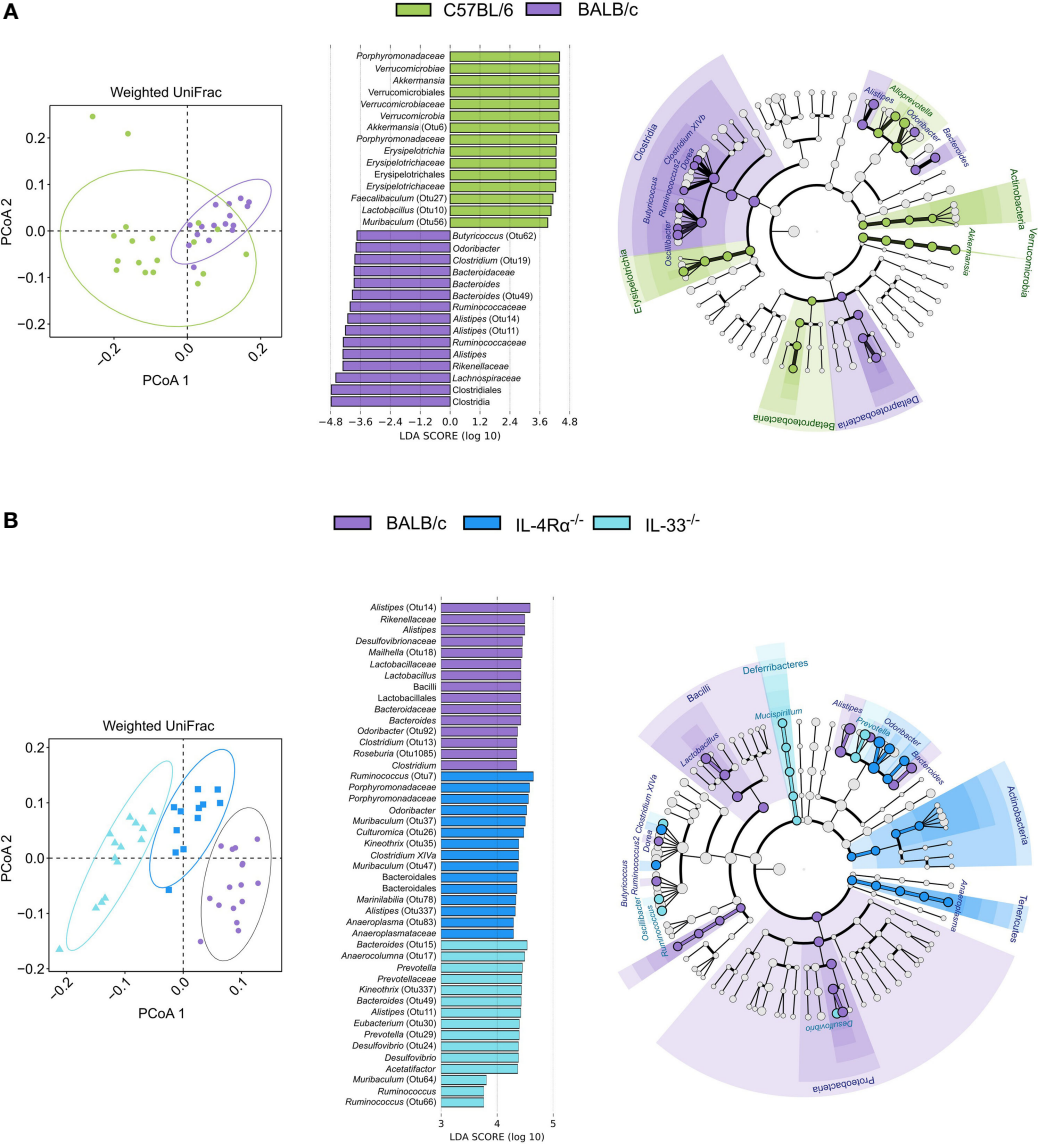
The naïve caecal mouse microbial community is immunocompetence-specific. Differences in microbial composition were assessed by AMOVA/ANOSIM, principal coordinate analysis (PCoA), and unsupervised clustering based on weighted UniFrac distances. Differentially abundant OTUs between genotypes were identified by linear discriminant analysis effect size (LEfSe, LDA > 3.0). **(A)** When comparing BALB/c (n = 16, purple) and C57BL/6 (n = 18, green) animals, only one cluster was observed as indicated by shapes in the PCoA despite AMOVA/ANOSIM indicating distinct differences between the two genotypes (*P* ≤ 0.014). Several differentially abundant OTUs distinctly associated with each cluster were observed where members of primarily the Clostridia class and Porphyromonadaceae family were associated with BALB/c and C57BL/6, respectively. **(B)** By comparing BALB/c wild-type (n = 16, purple), IL-4Rα^-/-^ (n = 12, blue), and IL-33^-/-^ (n = 14, light blue) mice, distinct clusters were observed for each genotype as indicated by the shapes in the PCoA (*P* < 0.001). Identified taxa are visualized in cladograms annotated at class and genus level, and the top 15 differentially abundant taxa for each genotype are visualized in bar plots. Additional taxonomic information can be found in **Supplementary Tables 5, 8**. AMOVA, analysis of molecular variance, ANOSIM, analysis of similarities (non-parametric); OTU, Operational taxonomic unit; LDA, linear discriminant analysis score; SD, standard deviation.

Because C57BL/6 and BALB/c animals were shown to be Th1- or Th2-leaning, we further studied microbial composition differences between these two of the most commonly utilized mouse strains in animal research. BALB/c animals showed significantly higher α-diversity compared to C57BL/6 (Shannon, *P* = 0.024), driven by higher richness (Chao1, *P* < 0.001, **Figure 4A**). As discussed above, unsupervised clustering analysis for microbial communities associated with C57BL/6 and BALB/c animals could not differentiate the two genotypes (**Figures 4B** and **5A**); however, the microbial composition of C57BL/6 and BALB/c animals was significantly distinct (*P* < 0.001, AMOVA/ANOSIM). Further analysis by LEfSe identified important differences in several distinct taxa, primarily within the *Lachnospiraceae*, *Porphyromonadaceae*, and *Ruminococcaceae* families (**Figure 5A** and **Supplementary Table 5**). Both these mouse strains were characterized by distinct *Alistipes* and *Bacteroides* spp. as well as members within the *Clostridium* XIVa cluster. Furthermore, C57BL/6 animals harbored elevated levels of *Enterorhabdus*, *Parabacteroides*, *Alloprevotella*, *Lactobacillus*, *Parasutterella*, and *Akkermansia* spp., whereas BALB/c animals were particularly characterized by *Odoribacter*, *Clostridium* XIVb, *Dorea*, *Ruminococcus*, *Butyricicoccus*, and *Oscillibacter* spp. Inferred metabolic activity displayed higher menaquinol biosynthesis in C57BL/6 animals (**Supplementary Table 6**).

As immunity and metagenomic data were available for individual animals, we sought independent correlations between CCGs and differentially abundant OTUs. In total, 31 OTUs were found to be directly or inversely associated with one or more of the analyzed CCGs in this study (Pearson Coefficient > 0.5 or < 0.05; FDR-corrected *P* < 0.001; **Supplementary Table 7**). Amongst these, *A. muciniphila* (Otu6, associated with C57BL/6) was found to be positively correlated with IL-2, MCP-1, and GM-CSF. Further, several distinct associations between CCGs and *Alistipes* spp. were observed. *A. timonensis* and *Bacteroides acidifaciens* (Otu67 and Otu81, respectively, both associated with C57BL/6) were positively correlated with CCGs such as IL-2, IL-10, IL1-β, and MCP-1, whereas other *Alistipes* spp. (Otu11, associated with BALB/c) were negatively correlated with these CCGs (**Supplementary Tables 2, 5, 7**).

### Th2-Deficiency in IL-33^-/-^ and IL-4Rα^-/-^ Mice Is Linked With Distinct Microbiota Profiles

To investigate the specific effect of Th2 immunodeficiency on caecal microbiota composition, IL-4Rα^-/-^ (n = 12) and IL-33^-/-^ (n = 14) animals were compared with their genetic background control BALB/c (n = 16). The overall diversity between wild-type BALB/c and Th2 deficient genotypes (IL-4Rα^-/-^ and IL-33^-/-^) was comparable (Shannon, *P >* 0.05, **Figure 4A**); however, IL-33^-/-^ mice showed a distinctly lower richness (Chao1, *P* < 0.001) compared to the IL-4Rα^-/-^ and wild-type animals.

Further, each group showed a unique microbial community composition (*P* < 0.001, AMOVA/ANOSIM). Differences between IL-4Rα^-/-^, IL-33^-/-^, and wild-type BALB/c were largely observed within the *Lachnospiraceae*, *Porphyromonadaceae*, and *Ruminococcaceae* families (**Figure 5B** and **Supplementary Table 8**). Common to all three mice strains were distinct members of the *Alistipes* genus. Both IL33^-/-^ and IL4^-/-^ mice harbored elevated levels of *Clostridium* XIVa and *Ruminococcus* spp., although the species differed between the two knockouts. Additionally, both BALB/c and IL-4Rα^-/-^ animals harbored elevated levels of *Odoribacter* and *Oscillibacter* spp., whereas both BALB/c and IL-33^-/-^ animals harbored elevated levels of *Bacteroides* spp. Finally, BALB/c mice were uniquely associated with *Alloprevotella*, *Butyricoccus*, *Dorea*, and *Lactobacillus* spp., whereas IL-33^-/-^ mice were associated with *Acetatifactor*, *Desulfovibrio*, and *Prevotella* spp., and IL-4Rα^-/-^ mice harbored elevated levels of *Anaeroplasma* and *Parasutterella* spp.

Differences in metabolic activity between the three BALB/c genotypes revealed that wild-type BALB/c harbored a microbiota associated with higher biosynthesis of amino acids cysteine, methionine, and histidine (the latter two are essential amino acids), purine (guanosine), pyrimidine, and thiazole. IL-4Rα^-/-^ mice were characterized by higher glycogen and starch degradation, succinate fermentation, and nitrate reduction. IL-33^-/-^ mice were characterized by higher pyrimidine and purine degradation, as well as ubiquinol, biotin, and lysine biosynthesis (**Supplementary Table 9**). These data suggest that the immunocompetence status is a strong driver of murine gut microbiota and could alter the microbial community-associated metabolic functions.

## Discussion

Observational studies conducted over the last decade have shown that the gut microbiota contributes to human health and, in part, this effect is mediated by regulating immune homeostasis (1, 2, 59–61). The Th1/Th2 balance is an essential factor for immune homeostasis as it maintains a critical equilibrium between pro- and anti-inflammatory states. Utilizing animal models that have substantial conclusive power in supporting hypotheses about cause-and-effect relationships, we show here that the immunity-microbiota relationship is bi-directional where alterations in the Th1/Th2 balance or a complete ablation of Th1/Th2 responses leads to major alterations in gut microbiota composition and function.

We first addressed wild-type C57BL/6 and BALB/c mice that have classically been considered as Th1- and Th2-dominant mouse strains, respectively (17, 19–21). Macrophages or splenic cells derived from C57BL/6 mice when challenged with lipopolysaccharides, macrophage-activating ligands, or other pro-inflammatory activators, secrete higher levels of Th1 cytokines like IFN-γ, TNF-α, and IL-12 than those from BALB/c mice (18, 19). Similarly, infection of C57BL/6 and BALB/c mice with a highly virulent *Pasteurella* strain was also shown to lead to Th1 and Th2 humoral responses, respectively (21). Thus, while the immunological response under pro-inflammatory challenge conditions is well-studied, how these commonly utilized mouse strains would behave in unstimulated conditions was not known. This is important as the baseline immunity could be a strong driver of gut microbiota composition that in turn might influence the disease phenotype modelled in these mouse models. We demonstrate here that while indeed naïve C57BL/6 mice display a robust Th1 polarization indicated by increased Th1 cells and pro-inflammatory cytokines, naïve BALB/c mice showed only a moderate Th2 cellular polarization along with a higher pro-inflammatory Th17 response. BALB/c mice also did not show any polarization in CCG profiles towards the Th2 side of the immune spectrum as major Th2 cytokines—IL-4, IL-5, and IL-13—showed similar levels as in C57BL/6 mice. These data fit well with prior studies where BALB/c mice when challenged with a pathogenic agent, showed increased IL-17 but lower Th1 (IFN-γ) cytokine levels compared to C57BL/6 mice (62, 63). Overall, our results suggest utilizing C57BL/6 in experiments where an exaggerated Th1 response is needed, such as bacterial or viral infections or to model cancer, while BALB/c could be utilized for experiments where immune homeostasis is a desirable endpoint.

As immunodeficient animals are also studied in context of various diseases, we investigated Th2-deficient IL-4Rα^-/-^ and IL-33^-/-^ knockouts bred in the BALB/c genetic background. IL-4 is a key cytokine in the differentiation of naive CD4+ T-cells into Th2 cells, which produce a panel of cytokines including IL-4, IL-5, IL-10, and IL-13 (64). IL-33 is different from other cytokines as (i) it is constitutively produced from the structural and lining cells that are exposed to the environment such as endothelial and epithelial cells, and (ii) IL-33 is localized predominantly in the host intranuclear compartment and in contrast to other cytokines, is released only after endothelial or epithelial cell damage where it functions as an alarmin. IL-33 has also recently received immense scientific attention as it plays an important role in type-2 innate immunity *via* activation of allergic inflammation-related eosinophils, basophils, mast cells, macrophages, and group 2 innate lymphoid cells through its receptor ST2 (64, 65). Due to deletion of Th2 or type-2 driven pathways, as expected, both IL-4Rα^-/-^ and IL-33^-/-^ mice displayed a Th1-polarized cytokine/chemokine profile despite increased IL-4 levels observed in IL-4Rα^-/-^ mice due to an absent receptor and a deficient ligand self-regulation. These data indicate that similar to C57BL/6 animals, mildly immunodeficient IL-4Rα^-/-^ and IL-33^-/-^ mice are also Th1 leaning. Interestingly, loss-of-function mutations in the *IL33* gene in humans show reduced blood eosinophil counts and lower susceptibility to Th2-driven diseases such as asthma (66, 67). It would be interesting to study how IL-33 haploinsufficiency affects the gut microbiota of *IL33* mutation carriers.

NSG mice, harboring a distinct genetic background combined with targeted mutations, are currently the most immunodeficient animals utilized in fundamental and preclinical research (68). Consistent with published data on deficient cellular immunity as well as deficiency in multiple cytokine signaling pathways (68–70), we also reported in this study a complete absence of innate and adaptive immune cells as well as very low levels of CCGs including STAT signaling pathway-linked IFN-γ levels. However, two cytokines, IL-4 and IP-10, were notably increased in NSG mice compared to all other genotypes. IP-10 is produced by a variety of immune and non-immune cells and by binding to Th1-associated CXCR3, it plays a primarily chemotactic role in inflammation by inducing several interferons (71). These data might have implications for studies utilizing NSG mice. For instance, these mice are commonly utilized in humanization experiments where different disease aspects are being studied including the impact of the human microbiome (72). Such studies should consider the influence of possibly elevated IL-4 levels, a dominant Th2 driving cytokine, or of IP-10 levels, a strong pro-inflammatory cytokine, on the dynamics of the studied phenotype.

Concerning the microbiota-immunity axis, while most studies have addressed how gut microbiota plays a vital role for the host in regulating the immune system, limited attention has been given to how inherent immunodeficiency impacts the gut microbiota (23–26, 73). One of these studies utilizing Th1- or Th2-deficient mice showed only subtle microbiota differences; however, this study was limited in resolution as a result of utilizing qPCR for microbial compositional assessment on a select number of gut microbes (25). Here, we applied 16S rRNA gene profiling to analyze the composition of two immunocompetent and three immunocompromised mouse strains in the same setting. The mouse caecum is known to display higher taxonomic complexity and bacterial load compared to fecal material (74, 75), and was therefore selected for analysis in this study. Independent *de novo* clustering based on caecal microbial composition significantly differentiated wild-type immunocompetent (C57BL/6 and BALB/c), mildly immunodeficient (IL-4Rα^-/-^ and IL-33^-/-^), and severely immunodeficient (NSG) mice. The identified differences were unique to the genotype class as sex, age, housing or other environmental or experimental conditions did not confound this clustering. Interestingly, despite the microbial composition being non-distinctive among the two wild-type strains, BALB/c mice showed higher microbial diversity and richness than C57BL/6 and NSG animals, and this was also inherent to the IL-4Rα^-/-^, but not to the IL-33^-/-^ genotype. Another immunity-related factor that could potentially contribute to overall microbial diversity/richness is the expression of the Toll-like receptor (TLR) (76), which has been shown to be highly diverse between BALB/c and C57BL/6 mice (77). Several studies have shown strong associations between reduced microbial diversity and unbalanced species composition with inflammation or disease (2). Our data further suggest that a Th1-leaning immunity in C57BL/6 mice, possibly along with other factors inherent to this mouse strain, leads to the observed decrease in microbial diversity (Shannon) as shown in several pro-inflammatory models (2, 27), and a decreased microbial diversity in turn could further contribute to the pro-inflammatory phenotype displayed by C57BL/6 compared to BALB/c mice, as shown in other studies (78). Of note, *de novo* clustering analysis of caecal microbial composition did not group IL-4Rα^-/-^ and IL-33^-/-^ with their wild-type BALB/c genetic background. Rather, BALB/c mice clustered with wild-type C57BL/6 mice from which it has been genetically apart for more than 100 years. This observation is important evidence supporting the premise that genomic loci other than those driving immunity are of lesser importance in driving gut microbiota in our study.

Lastly, we directed our attention to differentially abundant microbial species in the studied immunocompetent and immunocompromised mice, reflected as differences in microbiota-associated metabolic functions. We showed in this study that wild-type mice were enriched in bacteria such as *Lactobacillus* spp. (*L. animalis, L. murinus. L apodeme*), *A. muciniphila*, *Odoribacter*, *Bacteroides*, and *Alistipes* spp., which have previously been linked with the production of short-chain fatty acids (SCFAs) (5, 79) Several statistically significant differences were observed between C57BL/6 and BALB/c mice, and many of these SCFA-producing species were especially enriched in C57BL/6 mice compared to BALB/c mice. SCFAs such as acetate, propionate, and butyrate are fiber-derived metabolites and have been shown to suppress inflammatory pathways and maintain intestinal barrier function (5, 80), and a reduction of SCFA-producing organisms in humans is linked with inflammatory bowel disease (81, 82). Studies have also shown that SCFAs can induce CD4+ effector Th1 cells (83), and among all mouse strains studied here, we show that C57BL/6 carried the highest proportions of CD4+ Th1 cells. Moreover, in accordance with earlier data that microbiota-derived SCFA promote IL-10 production (84), we also show here that SCFA-producing microbe-enriched C57BL/6 display prominently higher levels of IL-10 compared to BALB/c and to the immunocompromised mouse strains studied here. Notably, in our study, C57BL/6 animals were enriched in *A. muciniphila*, a bacterium previously associated with Th1-polarization of T-cells (85). This fits well with our data of elevated levels of pro-inflammatory Th1 cytokines such as IL-2 and IL-1β in C57BL/6 mice compared to BALB/c mice, as has also been previously shown in these mice under challenged conditions (17–21). In contrast, immunodeficient (NSG, IL-4Rα^-/-^, IL-33^-/-^) mice were enriched in *Desulfovibrio* spp., a genus shown before to be associated with reduced levels of SCFAs (86). The NSG mice microbiota showed the most drastic changes compared to wild-type mice, which were also reflected as differences in metabolic associations in NSG strain; specifically, a reduced biosynthesis of essential amino acids, increased phospholipid biosynthesis and altered activity within glycolysis pathways. It is worth noting, however, that these results are an approximation and do not account for the actual abundances of SCFAs or other metabolites in the mouse cecum that could be quantitatively assessed through metabolomic analysis.

As limitations, while we in this study investigated commonly utilized C57BL/6 and BALB/c mice, several other strains are also utilized in the field such as DBA, A/J, CD1, and ICR mice. Some of these strains resemble either C57BL/6 or BALB/c (18), but studies dedicated to assessing the immune-microbiota relationship in these strains need to be addressed in future research. Moreover, while we specifically studied C57BL/6NRj and BALB/cJR bought from Janvier Laboratories, subtle differences exist in these strains from across different facilities and it would be interesting to study if these results are fully replicated in strains of these mice from other vendors. Lastly, it is also worth noting that we utilized an age group of 3–6 months for all genotypes and an additional 10 months NSG mouse group based on the premise that immunodeficiency might have an impact on ageing (30). However, because the 10-month-old age group of NSG mice, when analyzed together with 3–6 months old NSG mice, had no influence on the *de novo* clustering of groups, we believe that age should have had a marginal impact on the main outcome of this study.

Despite these limitations, we conclusively show that immune status is a strong driver of gut microbiota composition and, given the similarities between the human and mouse immune systems and gut microbiota, we believe that these findings will have important consequences for human immunodeficiency studies.

## Data Availability Statement

The datasets presented in this study can be found in an online repository. The name of the repository and accession number can be found below: https://www.ncbi.nlm.nih.gov/bioproject/PRJNA745937.

## Ethics Statement

The animal study was reviewed and approved by UA/UZA ethical review board 2016-08/2018-03/2019-25.

## Author Contributions

Conceptualization, SK-S. Animal experiments, VA and RJ. Sequencing, JR-R and BX. Data analysis and interpretation, MB, MM, and VA. Supervision, SK-S. Visualization, MB, MM, and VA. Writing – original draft, MB, VA, and SK-S. Writing – review and editing, MM, FD, B’sJ, JR-R, BX, RJ, AH, EC, SM-K, and HG. All authors contributed to the article and approved the submitted version.

## Funding

This research was funded by the Flemish Institute for Sciences and Technology (IWT-SBO), grant number 140746 and University of Antwerp-GOA grant number s30729. VVA and BSJ are PhD fellows of IWT/FWO.

## Conflict of Interest

ESC is employed by AstraZeneca.

The remaining authors declare that the research was conducted in the absence of any commercial or financial relationships that could be construed as a potential conflict of interest.

## Publisher’s Note

All claims expressed in this article are solely those of the authors and do not necessarily represent those of their affiliated organizations, or those of the publisher, the editors and the reviewers. Any product that may be evaluated in this article, or claim that may be made by its manufacturer, is not guaranteed or endorsed by the publisher.
