# Supplementary material for: Host Immunity Influences the Composition of Murine Gut Microbiota
Source: Front Immunol. 2022 Mar 15;13:828016. doi: 10.3389/fimmu.2022.828016 (PMC8965567; doi:10.3389/fimmu.2022.828016)
Supplement: Supplementary file 1 [file DataSheet_1.zip › Data Sheet 1/Supplementary Information.docx]

Supplementary Information

# Supplementary Results

The impact of baseline characteristics such as housing cage, date of handling, and animal experiment date was assessed for mice of the same genotype within the study. Specifically, beta diversity expressed by weighted unifrac distances were compared between groups using AMOVA followed by Bonferroni correction for multiple comparisons. Only two instances of cage effect comparisons showed small but significant differences (*P* ≤ 0.024, **Supplementary Table 1**) compared to larger effect sizes caused by immunity-driving genotypes. For all other cages, the differences were not significant under multiple comparison testing suggesting that the cage effect was smaller than the impact of inherent immunity as chief independent variables investigated in this study.

Next, we assessed the impact of gender and age on caecal microbial composition of mice with the same genotype as these are also important confounders. First addressing gender, weighted unifrac distances stratified by gender and genotype were utilized to conduct principal coordinate analysis of all samples in the study (**Supplementary Figure 2A**). Comparisons of beta diversity between stratified groups revealed differences in microbial composition by AMOVA (*P* < 0.05). However, when subjecting these to *de novo* clustering using the Dirichlet multinomial mixtures (DMM), all stratified groups resulted in one single cluster. When subjecting all animals from different genotype groups to the same analysis, only three clusters were identified, which corresponded to the immunocompetency as shown in **Figure 4B** in the manuscript.

All animals in the wild-type C57BL/6 and BALB/c as well as IL-33^-/-^ and IL-4Rα^-/-^ groups all contained mice with an age range of 3–6 months. This was done as ageing is an important factor influencing gut microbiota (1–5), and because we amongst others have shown that different genotypes that might age differently (6,7), we opted for this age range as mature adult mice range in age from 3–6 months (8). The NOD.Cg-Prkdcscid Il2rgtm1Wjl/SzJ (NSG, NOD scid gamma) mice utilized in this study were the most vulnerable group because of being most immunodeficient. Also, because experimental procedures commonly applied in NSG involves irradiation, grafting, and graft maturation take considerable time and the mice age before they could be utilized in experiments, therefore besides 3 months, we also studied mice of 10 months old. Animals were stratified by age to assess its impact on the microbiota (**Supplementary Figure 2B**). Similar to the gender assessment, differences in beta diversity were observed between the groups when assessed by AMOVA (*P* < 0.05), but these were not detected by *de novo* clustering when conducted individually, nor when clustering all samples.

These data all suggest that housing conditions, gender, and age are not confounding the observed relationship between alterations in innate immune status and microbiota identified in the current study.

# References

1. Nagpal R, Mainali R, Ahmadi S, Wang S, Singh R, Kavanagh K, Kitzman DW, Kushugulova A, Marotta F, Yadav H. Gut microbiome and aging: Physiological and mechanistic insights. *Nutr Heal Aging* (2018) **4**:267–285. doi:10.3233/NHA-170030

2. Boehme M, Guzzetta KE, Bastiaanssen TFS, van de Wouw M, Moloney GM, Gual-Grau A, Spichak S, Olavarría-Ramírez L, Fitzgerald P, Morillas E, et al. Microbiota from young mice counteracts selective age-associated behavioral deficits. *Nat Aging* (2021) **1**:666–676. doi:10.1038/s43587-021-00093-9

3. Binyamin D, Werbner N, Nuriel-Ohayon M, Uzan A, Mor H, Abbas A, Ziv O, Teperino R, Gutman R, Koren O. The aging mouse microbiome has obesogenic characteristics. *Genome Med* (2020) **12**:87. doi:10.1186/s13073-020-00784-9

4. Lynn MA, Eden G, Ryan FJ, Bensalem J, Wang X, Blake SJ, Choo JM, Chern YT, Sribnaia A, James J, et al. The composition of the gut microbiota following early-life antibiotic exposure affects host health and longevity in later life. *Cell Rep* (2021) **36**:109564. doi:10.1016/j.celrep.2021.109564

5. Langille MGI, Meehan CJ, Koenig JE, Dhanani AS, Rose RA, Howlett SE, Beiko RG. Microbial shifts in the aging mouse gut. *Microbiome* (2014) doi:10.1186/s40168-014-0050-9

6. Wils H, Kleinberger G, Pereson S, Janssens J, Capell A, Van Dam D, Cuijt I, Joris G, De Deyn PP, Haass C, et al. Cellular ageing, increased mortality and FTLD-TDP-associated neuropathology in progranulin knockout mice. *J Pathol* (2012)n/a-n/a. doi:10.1002/path.4043

7. Pinchuk LM, Filipov NM. Differential effects of age on circulating and splenic leukocyte populations in C57BL/6 and BALB/c male mice. *Immun Ageing* (2008) **5**:1. doi:10.1186/1742-4933-5-1

8. Flurkey K, Mcurrer J, Harrison D. “Mouse Models in Aging Research,” in *The Mouse in Biomedical Research* (Elsevier), 637–672. doi:10.1016/B978-012369454-6/50074-1

# Supplementary Figures


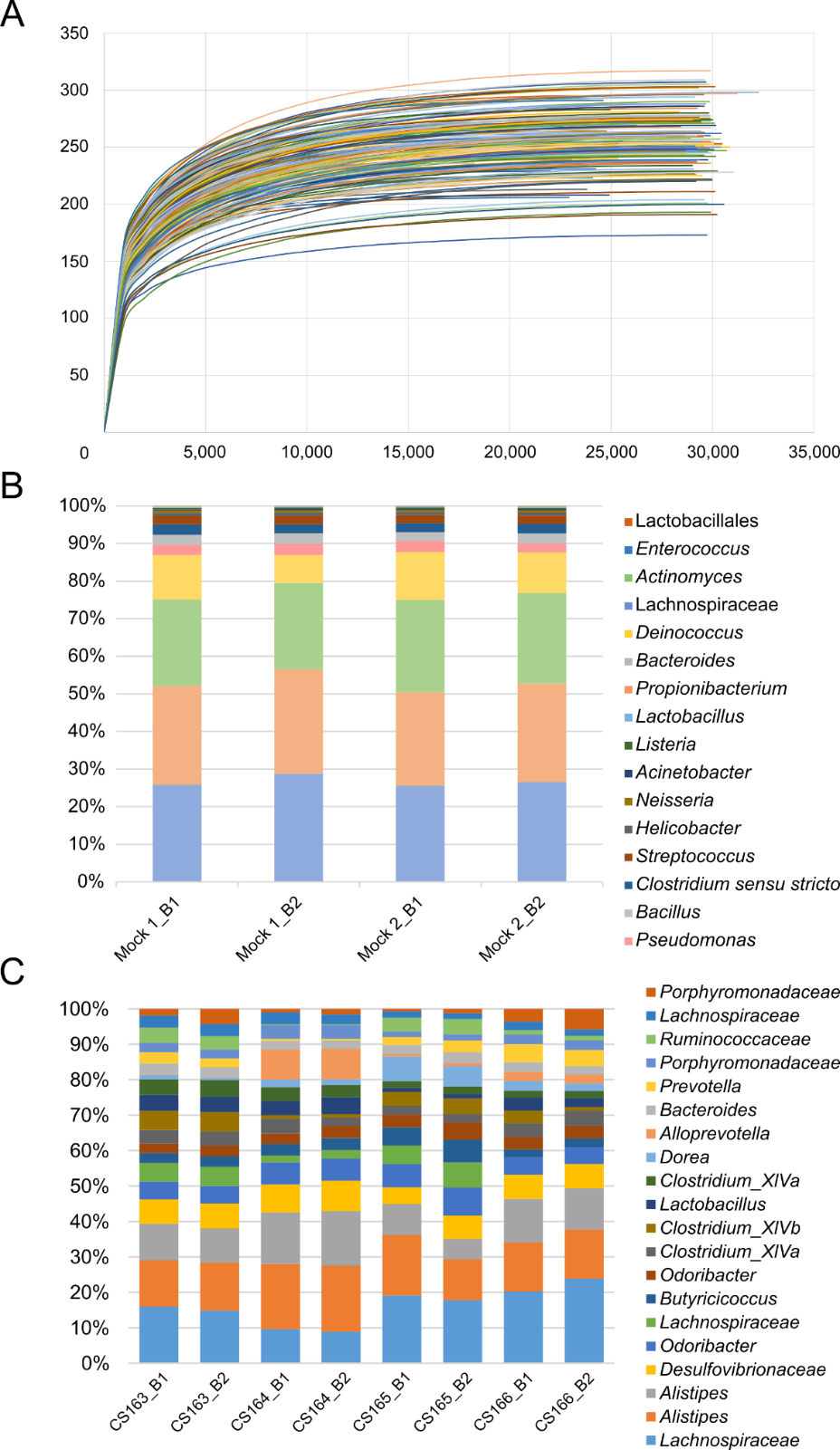


**Supplementary Figure 1.** Control of sequencing data. **(A)** To ensure that a sufficient sequencing depth was obtained in the study for capturing sample diversity across all sequenced samples, rarefaction curves (number of OTUs on the y-axis, sequence reads on the x-axis) were plotted. **(B)** Relative abundances of the top 20 OTUs observed in the mock communities sequenced in batches 1 (B1) and 2 (B2) were compared, which revealed a Pearson correlation of >99%. **(C)** Similarly, by comparing positive sample controls sequenced in the two batches, a Pearson correlation of 97% was observed.


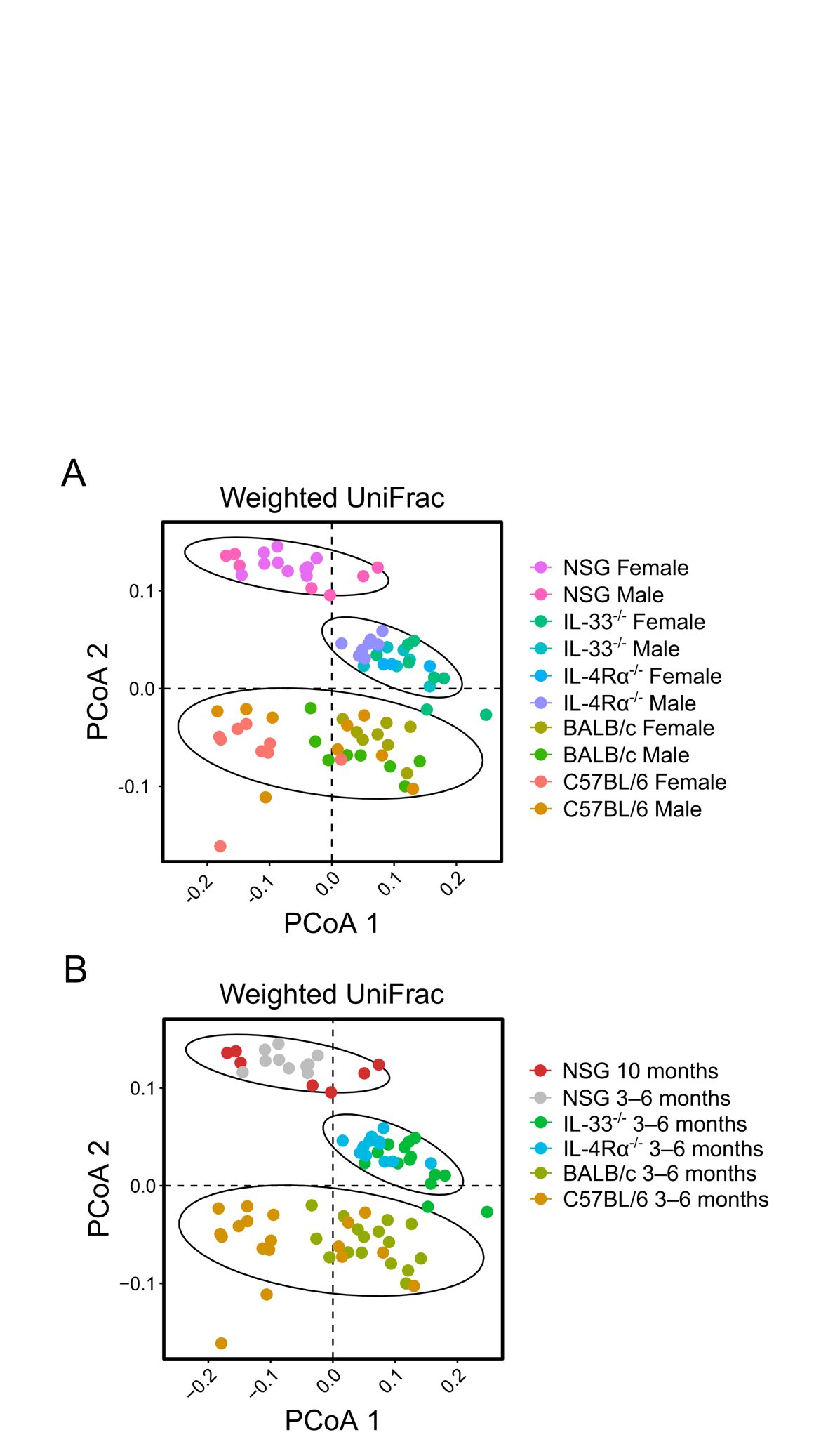


**Supplementary Figure 2.** Principal coordinate analysis was performed by utilizing weighted unifrac beta diversity distances for all samples subjected to 16S rRNA gene profiling in the study. Ellipses in the plots indicate the clusters identified by *de novo* clustering using the Dirichlet multinomial mixtures model. The plot illustrates analyzed samples stratified by gender **(A)** and age **(B)**.


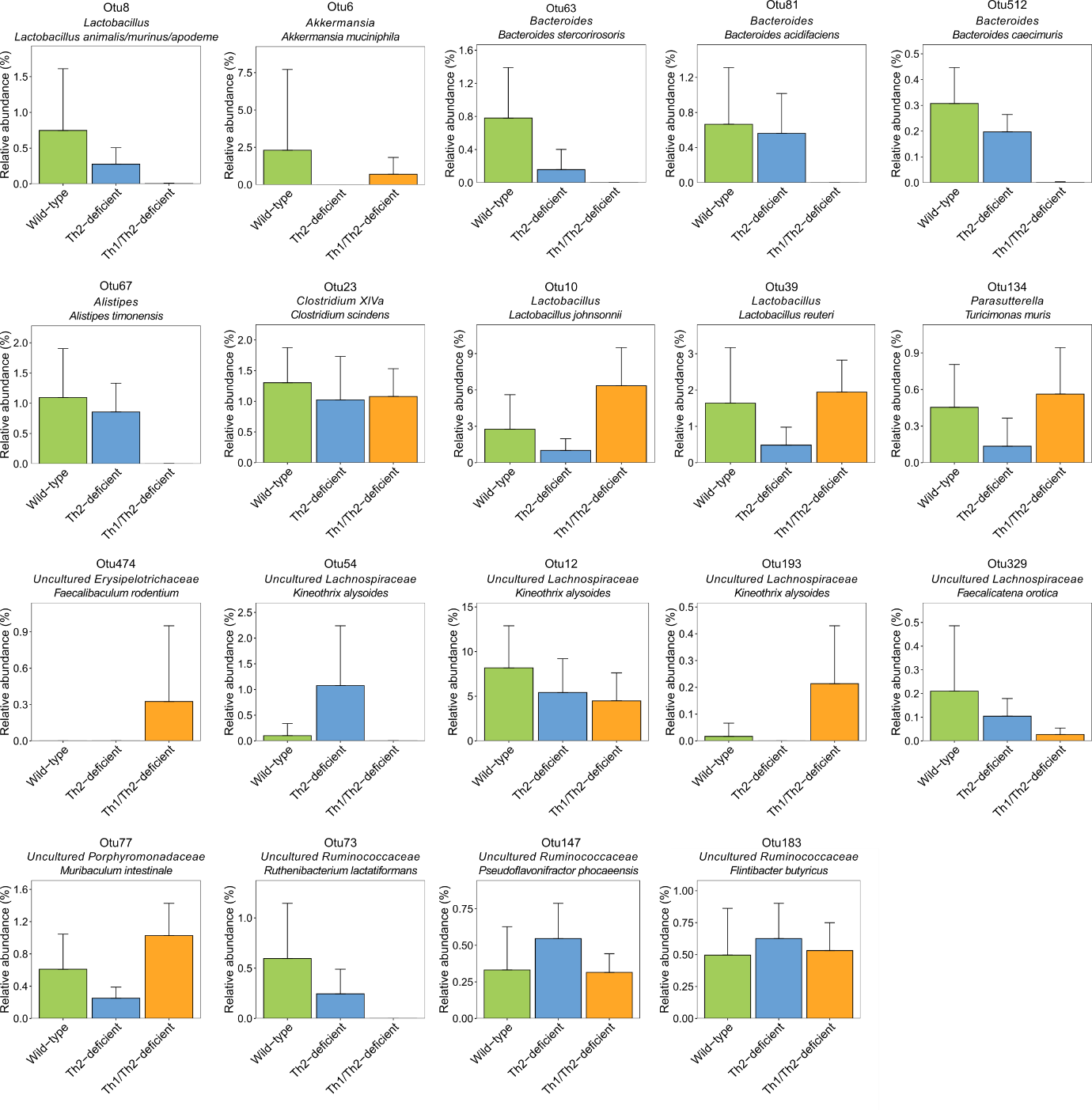


**Supplementary Figure 3.** Relative abundances of distinct OTUs classified to species level associated with *de novo* clusters. The relative abundances of differential abundant OTUs identified by linear discriminant analysis effect size (LEfSe, LDA > 3.0) that could be classified to species levels (**Supplementary Table 2**) were compared between the three clusters identified after unsupervised, de novo clustering of all caecal samples collected from SPF-housed wild-type (green) C57BL/6 (n = 18) and BALB/c (n = 16), Th2-deficient (blue) BALB/c IL-4Rα^-/-^ (n = 12) and IL-33^-/-^ (n = 14), and Th1/Th2-deficient NSG (orange, n = 17) animals. Bar plot error bars indicate SD. WT: wild type; OTU: Operational taxonomical unit; LDA: linear discriminant analysis score; SD: standard deviation.

**Supplementary Table 1.** To assess the cage effect and its impact on caecal microbial composition within different genotypes, beta diversity expressed by weighted unifrac distances was compared between cages containing at least three animals using AMOVA, followed by Bonferroni correction for multiple comparisons.

| **Genotype** | **Cage 1** | **Cage 2** | **Corrected *P*-value** |
| --- | --- | --- | --- |
| C57BL/6 | 1650_20 | 1651_20 | 0.116 |
|  | 1650_20 | 1652_20 | 1 |
|  | 1650_20 | 1653_20 | 0.808 |
|  | 1651_20 | 1652_20 | **0.004** |
|  | 1651_20 | 1653_20 | 0.052 |
|  | 1652_20 | 1653_20 | 0.128 |
| IL-4Rα^-/-^ | 1946_19 | 1948_19 | 0.072 |
|  | 1946_19 | 2533_19 | 0.102 |
|  | 1948_19 | 2533_19 | 0.084 |
| IL-33^-/-^ | 2216_19 | 2217_19 | 0.188 |
|  | 2216_19 | 2218_19 | 0.092 |
|  | 2216_19 | 2532_19 | **0.024** |
|  | 2217_19 | 2218_19 | 0.212 |
|  | 2217_19 | 2532_19 | 0.2 |
|  | 2218_19 | 2532_19 | 0.42 |
| BALB/c wild type | 4114_19 | 4115_19 | 0.56 |
|  | 4114_19 | 4116_19 | 0.228 |
|  | 4114_19 | 4117_19 | 0.628 |
|  | 4115_19 | 4116_19 | 0.068 |
|  | 4115_19 | 4117_19 | 0.152 |
|  | 4116_19 | 4117_19 | 0.972 |
| NSG | 770_20 | 771_20 | 0.277 |

**Supplementary Table 2.** Differentiation in microbial composition between identified *de novo* clusters. Distinctions in microbiota composition were assessed using LEfSe where OTUs with an LDA > 3.0. The most representative sequence of each OTU was blasted against the NCBI 16S gene database to determine the associated species classification. Only hits with a percent identity of 97% were considered valid and are displayed in the table. - indicates that no valid hits were achieved (percent identity < 97.0%).

| **Group** | **OTU** | **LDA** | ***P*-value** | **Genus** | **NCBI classification** | | **Query cover (%)** | **Percentage identity (%)** |
| --- | --- | --- | --- | --- | --- | --- | --- | --- |
| Th2-deficient | Otu49 | 3.81 | 1.32E-09 | *Bacteroides* | **-** | **-** | **-** | **-** |
|  | Otu78 | 3.63 | 2.29E-09 | Uncultured Bacteroidales | **-** | **-** | **-** | **-** |
|  | Otu89 | 3.69 | 4.04E-08 | *Barnesiella* | **-** | **-** | **-** | **-** |
|  | Otu50 | 3.7 | 1.32E-04 | Uncultured *Porphyromonadaceae* | **-** | **-** | **-** | **-** |
|  | Otu87 | 3.6 | 1.08E-13 | Uncultured *Porphyromonadaceae* | **-** | **-** | **-** | **-** |
|  | Otu102 | 3.19 | 1.21E-05 | Uncultured *Porphyromonadaceae* | **-** | **-** | **-** | **-** |
|  | Otu124 | 3.42 | 2.56E-07 | Uncultured *Porphyromonadaceae* | **-** | **-** | **-** | **-** |
|  | Otu217 | 3.03 | 1.57E-05 | Uncultured *Porphyromonadaceae* | **-** | **-** | **-** | **-** |
|  | Otu29 | 4.03 | 1.59E-09 | *Prevotella* | **-** | **-** | **-** | **-** |
|  | Otu11 | 4.18 | 7.09E-07 | *Alistipes* | **-** | **-** | **-** | **-** |
|  | Otu337 | 3.55 | 8.21E-08 | *Alistipes* | **-** | **-** | **-** | **-** |
|  | Otu35 | 3.84 | 5.41E-08 | *Clostridium* *XlVa* | **-** | **-** | **-** | **-** |
|  | Otu7 | 4.47 | 1.40E-05 | Uncultured *Lachnospiraceae* | **-** | **-** | **-** | **-** |
|  | Otu15 | 4.33 | 6.61E-12 | Uncultured *Lachnospiraceae* | **-** | **-** | **-** | **-** |
|  | Otu17 | 3.87 | 1.13E-07 | Uncultured *Lachnospiraceae* | **-** | **-** | **-** | **-** |
|  | Otu32 | 3.53 | 4.15E-04 | Uncultured *Lachnospiraceae* | **-** | **-** | **-** | **-** |
|  | Otu44 | 3.24 | 9.86E-07 | Uncultured *Lachnospiraceae* | **-** | **-** | **-** | **-** |
|  | Otu54 | 3.78 | 4.89E-08 | Uncultured *Lachnospiraceae* | *Kineothrix* | *alysoides* | 100 | 97.26 |
|  | Otu108 | 3.02 | 1.80E-04 | Uncultured *Lachnospiraceae* | **-** | **-** | **-** | **-** |
|  | Otu112 | 3.08 | 1.24E-04 | Uncultured *Lachnospiraceae* | **-** | **-** | **-** | **-** |
|  | Otu127 | 3.1 | 4.27E-05 | Uncultured *Lachnospiraceae* | **-** | **-** | **-** | **-** |
|  | Otu129 | 3.39 | 1.30E-07 | Uncultured *Lachnospiraceae* | **-** | **-** | **-** | **-** |
|  | Otu157 | 3.21 | 6.44E-06 | Uncultured *Lachnospiraceae* | **-** | **-** | **-** | **-** |
|  | Otu106 | 3.7 | 1.24E-05 | *Ruminococcus2* | **-** | **-** | **-** | **-** |
|  | Otu70 | 3.19 | 3.23E-05 | *Oscillibacter* | **-** | **-** | **-** | **-** |
|  | Otu30 | 3.7 | 2.09E-12 | Uncultured *Ruminococcaceae* | **-** | **-** | **-** | **-** |
|  | Otu74 | 3.52 | 1.07E-05 | Uncultured *Ruminococcaceae* | **-** | **-** | **-** | **-** |
|  | Otu147 | 3.25 | 9.69E-04 | Uncultured *Ruminococcaceae* | *Pseudoflavonifractor* | *phocaeensis* | 100 | 97.28 |
|  | Otu184 | 3.19 | 1.25E-07 | Uncultured *Ruminococcaceae* | **-** | **-** | **-** | **-** |
|  | Otu66 | 3.73 | 4.49E-08 | *Ruminococcus* | **-** | **-** | **-** | **-** |
|  | Otu24 | 3.85 | 2.11E-13 | *Desulfovibrio* | **-** | **-** | **-** | **-** |
| NSG | Otu28 | 3.59 | 1.22E-11 | *Barnesiella* | **-** | **-** | **-** | **-** |
|  | Otu26 | 3.58 | 7.77E-03 | *Odoribacter* | **-** | **-** | **-** | **-** |
|  | Otu47 | 3.94 | 3.63E-04 | Uncultured *Porphyromonadaceae* | **-** | **-** | **-** | **-** |
|  | Otu56 | 3.83 | 3.81E-04 | Uncultured *Porphyromonadaceae* | **-** | **-** | **-** | **-** |
|  | Otu64 | 3.61 | 7.92E-06 | Uncultured *Porphyromonadaceae* | **-** | **-** | **-** | **-** |
|  | Otu77 | 3.62 | 5.96E-09 | Uncultured *Porphyromonadaceae* | *Muribaculum* | *intestinale* | 100 | 99.76 |
|  | Otu99 | 3.56 | 6.61E-13 | Uncultured *Porphyromonadaceae* | **-** | **-** | **-** | **-** |
|  | Otu145 | 3.51 | 9.50E-12 | Uncultured *Porphyromonadaceae* | **-** | **-** | **-** | **-** |
|  | Otu150 | 3.52 | 1.03E-11 | Uncultured *Porphyromonadaceae* | **-** | **-** | **-** | **-** |
|  | Otu160 | 3.45 | 3.33E-16 | Uncultured *Porphyromonadaceae* | **-** | **-** | **-** | **-** |
|  | Otu221 | 3.22 | 5.71E-14 | Uncultured *Porphyromonadaceae* | **-** | **-** | **-** | **-** |
|  | Otu252 | 3.01 | 4.61E-13 | Uncultured *Porphyromonadaceae* | **-** | **-** | **-** | **-** |
|  | Otu280 | 3.2 | 9.99E-16 | Uncultured *Porphyromonadaceae* | **-** | **-** | **-** | **-** |
|  | Otu86 | 3.77 | 4.49E-05 | *Alistipes* | **-** | **-** | **-** | **-** |
|  | Otu10 | 4.45 | 1.66E-06 | *Lactobacillus* | *Lactobacillus* | *johnsonnii* | 100 | 100 |
|  | Otu39 | 3.89 | 1.00E-04 | *Lactobacillus* | *Lactobacillus* | *reuteri* | 100 | 100 |
|  | Otu20 | 4.39 | 2.38E-11 | Uncultured Clostridiales | **-** | **-** | **-** | **-** |
|  | Otu85 | 3.8 | 9.99E-16 | Uncultured Clostridiales | **-** | **-** | **-** | **-** |
|  | Otu111 | 3.27 | 9.56E-03 | Uncultured Clostridiales | **-** | **-** | **-** | **-** |
|  | Otu149 | 3.06 | 3.75E-10 | Uncultured Clostridiales | **-** | **-** | **-** | **-** |
|  | Otu25 | 3.75 | 1.60E-02 | Uncultured *Lachnospiraceae* | **-** | **-** | **-** | **-** |
|  | Otu31 | 3.13 | 5.97E-03 | Uncultured *Lachnospiraceae* | **-** | **-** | **-** | **-** |
|  | Otu58 | 3.09 | 5.10E-09 | Uncultured *Lachnospiraceae* | **-** | **-** | **-** | **-** |
|  | Otu90 | 3.37 | 8.21E-08 | Uncultured *Lachnospiraceae* | **-** | **-** | **-** | **-** |
|  | Otu1899 | 3.72 | 1.26E-10 | Uncultured *Lachnospiraceae* | **-** | **-** | **-** | **-** |
|  | Otu126 | 3.16 | 5.31E-04 | Uncultured *Lachnospiraceae* | **-** | **-** | **-** | **-** |
|  | Otu139 | 3.36 | 8.05E-06 | Uncultured *Lachnospiraceae* | **-** | **-** | **-** | **-** |
|  | Otu117 | 3.24 | 2.04E-03 | Uncultured *Ruminococcaceae* | **-** | **-** | **-** | **-** |
|  | Otu474 | 3.24 | 9.55E-13 | Uncultured *Erysipelotrichaceae* | *Faecalibaculum* | *rodentium* | 100 | 99.3 |
|  | Otu203 | 3.15 | 2.70E-08 | Uncultured Firmicutes | **-** | **-** | **-** | **-** |
|  | Otu134 | 3.41 | 4.17E-06 | *Parasutterella* | *Turicimonas* | *muris* | 100 | 100 |
|  | Otu83 | 3.73 | 5.56E-14 | *Anaeroplasma* | **-** | **-** | **-** | **-** |
| Wild type | Otu130 | 3.09 | 1.34E-07 | Uncultured Bacteria | **-** | **-** | **-** | **-** |
|  | Otu63 | 3.65 | 6.02E-11 | *Bacteroides* | *Bacteroides* | *stercorirosoris* | 100 | 98.82 |
|  | Otu81 | 3.48 | 2.48E-09 | *Bacteroides* | *Bacteroides* | *acidifaciens* | 100 | 99.53 |
|  | Otu512 | 3.11 | 4.99E-11 | *Bacteroides* | *Bacteroides* | *caecimuris* | 100 | 99.76 |
|  | Otu162 | 3.5 | 5.01E-12 | Uncultured Bacteroidales | **-** | **-** | **-** | **-** |
|  | Otu196 | 3.18 | 1.92E-10 | *Barnesiella* | **-** | **-** | **-** | **-** |
|  | Otu92 | 3.65 | 1.35E-06 | *Odoribacter* | **-** | **-** | **-** | **-** |
|  | Otu165 | 3.04 | 2.41E-09 | *Parabacteroides* | *Parabacteroides* | *goldsteinii* | 100 | 100 |
|  | Otu46 | 3.54 | 7.79E-09 | Uncultured *Porphyromonadaceae* | **-** | **-** | **-** | **-** |
|  | Otu91 | 3.52 | 1.51E-11 | Uncultured *Porphyromonadaceae* | **-** | **-** | **-** | **-** |
|  | Otu107 | 3.5 | 3.80E-14 | Uncultured *Porphyromonadaceae* | **-** | **-** | **-** | **-** |
|  | Otu118 | 3.47 | 2.24E-13 | Uncultured *Porphyromonadaceae* | **-** | **-** | **-** | **-** |
|  | Otu136 | 3.21 | 5.13E-09 | Uncultured *Porphyromonadaceae* | **-** | **-** | **-** | **-** |
|  | Otu175 | 3.19 | 4.15E-09 | Uncultured *Porphyromonadaceae* | **-** | **-** | **-** | **-** |
|  | Otu21 | 4.1 | 1.47E-12 | *Alloprevotella* | **-** | **-** | **-** | **-** |
|  | Otu14 | 4.37 | 8.82E-09 | *Alistipes* | **-** | **-** | **-** | **-** |
|  | Otu67 | 3.7 | 4.81E-09 | *Alistipes* | *Alistipes* | *timonensis* | 100 | 98.1 |
|  | Otu229 | 3.27 | 2.73E-08 | *Alistipes* | **-** | **-** | **-** | **-** |
|  | Otu8 | 3.57 | 4.85E-11 | *Lactobacillus* | *Lactobacillus* | *animalis* | 100 | 100 |
|  | Otu279 | 3.06 | 2.54E-09 | Uncultured Clostridiales | **-** | **-** | **-** | **-** |
|  | Otu33 | 3.29 | 7.83E-04 | *Clostridium XlVa* | *Kineothrix* | *alysoides* | 100 | 98.51 |
|  | Otu456 | 3.67 | 7.92E-07 | *Clostridium XlVa* | **-** | **-** | **-** | **-** |
|  | Otu96 | 3.07 | 8.86E-03 | *Dorea* | **-** | **-** | **-** | **-** |
|  | Otu5 | 4.21 | 5.68E-04 | Uncultured *Lachnospiraceae* | **-** | **-** | **-** | **-** |
|  | Otu12 | 4.29 | 5.21E-03 | Uncultured *Lachnospiraceae* | *Kineothrix* | *alysoides* | 100 | 97.01 |
|  | Otu13 | 3.78 | 7.74E-03 | Uncultured *Lachnospiraceae* | **-** | **-** | **-** | **-** |
|  | Otu19 | 3.68 | 4.03E-02 | Uncultured *Lachnospiraceae* | **-** | **-** | **-** | **-** |
|  | Otu1085 | 3.63 | 1.53E-04 | Uncultured *Lachnospiraceae* | **-** | **-** | **-** | **-** |
|  | Otu158 | 3.19 | 4.58E-08 | Uncultured *Lachnospiraceae* | **-** | **-** | **-** | **-** |
|  | Otu678 | 3.39 | 8.62E-11 | Uncultured *Lachnospiraceae* | **-** | **-** | **-** | **-** |
|  | Otu52 | 3.29 | 1.22E-04 | Uncultured *Lachnospiraceae* | **-** | **-** | **-** | **-** |
|  | Otu57 | 3.18 | 5.98E-05 | Uncultured *Lachnospiraceae* | **-** | **-** | **-** | **-** |
|  | Otu902 | 3.68 | 8.63E-11 | Uncultured *Lachnospiraceae* | **-** | **-** | **-** | **-** |
|  | Otu94 | 3.08 | 1.53E-03 | Uncultured *Lachnospiraceae* | **-** | **-** | **-** | **-** |
|  | Otu105 | 3.01 | 3.93E-10 | Uncultured *Lachnospiraceae* | **-** | **-** | **-** | **-** |
|  | Otu329 | 3.18 | 5.07E-06 | Uncultured *Lachnospiraceae* | *Faecalicatena* | *orotica* | 100 | 98.26 |
|  | Otu73 | 3.47 | 2.95E-10 | Uncultured *Ruminococcaceae* | *Ruthenibacterium* | *lactatiformans* | 100 | 97.26 |
|  | Otu18 | 4.08 | 1.05E-12 | Uncultured *Desulfovibrionaceae* | **-** | **-** | **-** | **-** |
|  | Otu6 | 4.21 | 6.15E-06 | *Akkermansia* | *Akkermansia* | *muciniphila* | 100 | 100 |

**Supplementary Table 3.** Each *de novo* cluster is associated with distinct metabolic activity. Distinctions in metabolic activity were assessed by conducting functional analysis with PICRUSt using the EC database, followed by non-parametric Kruskal-Wallis testing with Welch’s uncorrected post-hoc test and Benjamini-Hochberg FDR correction. Only results with an effect size > 0.5 are reported.

| Group | description | p-values (corrected) | Effect size | Th2-deficient: mean rel. freq. (%) | Th2-deficient: std. dev. (%) | Th1/Th2-deficient: mean rel. freq. (%) | Th1/Th2-deficient: std. dev. (%) | Wild type mean rel. freq. (%) | Wild type std. dev. (%) |
| --- | --- | --- | --- | --- | --- | --- | --- | --- | --- |
| Th2-Deficient | L-lysine biosynthesis I | 4.34E-10 | 0.60 | 0.804 | 0.039 | 0.766 | 0.040 | 0.691 | 0.043 |
|  | photorespiration | 1.54E-11 | 0.75 | 0.032 | 0.014 | 0.029 | 0.008 | 0.000 | 0.000 |
|  | L-lysine biosynthesis III | 6.91E-10 | 0.55 | 0.985 | 0.023 | 0.964 | 0.034 | 0.899 | 0.043 |
|  | biotin biosynthesis II | 1.60E-11 | 0.70 | 0.012 | 0.006 | 0.011 | 0.004 | 0.000 | 0.000 |
|  | guanosine nucleotides degradation III | 1.34E-08 | 0.52 | 0.356 | 0.088 | 0.346 | 0.118 | 0.162 | 0.078 |
|  | superpathway of sulfolactate degradation | 1.48E-11 | 0.69 | 0.012 | 0.006 | 0.011 | 0.004 | 0.000 | 0.000 |
|  | superpathway of glucose and xylose degradation | 2.14E-08 | 0.74 | 0.262 | 0.036 | 0.092 | 0.033 | 0.258 | 0.048 |
|  | adenosine nucleotides degradation II | 6.84E-09 | 0.52 | 0.332 | 0.088 | 0.330 | 0.117 | 0.144 | 0.071 |
| Th1/Th2-deficient | glycolysis III (from glucose) | 7.92E-09 | 0.67 | 1.017 | 0.022 | 1.099 | 0.016 | 1.002 | 0.034 |
|  | superpathway of arginine and polyamine biosynthesis | 1.11E-07 | 0.53 | 0.099 | 0.025 | 0.215 | 0.034 | 0.121 | 0.054 |
|  | glycolysis I (from glucose 6-phosphate) | 3.98E-08 | 0.53 | 0.775 | 0.051 | 0.972 | 0.072 | 0.854 | 0.075 |
|  | acetylene degradation | 2.85E-10 | 0.63 | 0.516 | 0.054 | 0.650 | 0.102 | 0.369 | 0.094 |
|  | hexitol fermentation to lactate, formate, ethanol and acetate | 7.61E-09 | 0.75 | 0.032 | 0.017 | 0.142 | 0.038 | 0.044 | 0.021 |
|  | peptidoglycan biosynthesis I (meso-diaminopimelate containing) | 5.28E-09 | 0.66 | 0.881 | 0.017 | 0.964 | 0.030 | 0.863 | 0.034 |
|  | superpathway of phospholipid biosynthesis I (bacteria) | 2.58E-09 | 0.60 | 0.919 | 0.041 | 0.993 | 0.028 | 0.875 | 0.038 |
|  | superpathway of polyamine biosynthesis I | 1.58E-07 | 0.53 | 0.054 | 0.014 | 0.129 | 0.025 | 0.069 | 0.034 |
|  | urea cycle | 1.04E-08 | 0.84 | 0.032 | 0.011 | 0.248 | 0.074 | 0.041 | 0.021 |
|  | CDP-diacylglycerol biosynthesis I | 4.25E-10 | 0.66 | 1.036 | 0.036 | 1.121 | 0.025 | 0.988 | 0.041 |
|  | ubiquinol-7 biosynthesis (prokaryotic) | 1.34E-11 | 0.73 | 0.014 | 0.006 | 0.014 | 0.004 | 0.001 | 0.001 |
|  | ubiquinol-9 biosynthesis (prokaryotic) | 1.68E-11 | 0.73 | 0.014 | 0.006 | 0.014 | 0.004 | 0.001 | 0.001 |
|  | ubiquinol-10 biosynthesis (prokaryotic) | 1.58E-11 | 0.73 | 0.014 | 0.006 | 0.014 | 0.004 | 0.001 | 0.001 |
|  | purine nucleotides degradation II (aerobic) | 3.13E-09 | 0.57 | 0.298 | 0.050 | 0.342 | 0.079 | 0.178 | 0.059 |
|  | peptidoglycan biosynthesis III (mycobacteria) | 9.07E-09 | 0.60 | 0.877 | 0.018 | 0.950 | 0.025 | 0.860 | 0.036 |
|  | UDP-N-acetylmuramoyl-pentapeptide biosynthesis II (lysine-containing) | 3.81E-09 | 0.63 | 0.892 | 0.020 | 0.977 | 0.027 | 0.871 | 0.039 |
|  | UDP-N-acetylmuramoyl-pentapeptide biosynthesis I (meso-diaminopimelate containing) | 3.82E-09 | 0.66 | 0.892 | 0.018 | 0.980 | 0.031 | 0.871 | 0.037 |
|  | adenine and adenosine salvage III | 1.52E-11 | 0.62 | 0.879 | 0.017 | 0.940 | 0.034 | 0.831 | 0.041 |
|  | ubiquinol-8 biosynthesis (prokaryotic) | 1.41E-11 | 0.73 | 0.014 | 0.006 | 0.014 | 0.004 | 0.001 | 0.001 |
|  | pyrimidine deoxyribonucleosides salvage | 5.46E-09 | 0.55 | 0.483 | 0.034 | 0.614 | 0.053 | 0.549 | 0.045 |
|  | superpathway of pyrimidine nucleobases salvage | 3.42E-08 | 0.67 | 1.034 | 0.021 | 1.153 | 0.048 | 1.028 | 0.038 |
|  | adenosine ribonucleotides de novo biosynthesis | 3.61E-09 | 0.64 | 1.013 | 0.023 | 1.085 | 0.027 | 0.989 | 0.031 |
|  | guanosine ribonucleotides de novo biosynthesis | 6.00E-09 | 0.63 | 0.870 | 0.018 | 0.946 | 0.031 | 0.847 | 0.034 |
|  | superpathway of adenosine nucleotides de novo biosynthesis I | 5.55E-09 | 0.61 | 0.948 | 0.021 | 1.025 | 0.039 | 0.920 | 0.035 |
|  | taxadiene biosynthesis (engineered) | 1.48E-08 | 0.51 | 0.149 | 0.067 | 0.404 | 0.082 | 0.319 | 0.121 |
|  | superpathway of purine deoxyribonucleosides degradation | 1.45E-11 | 0.70 | 0.563 | 0.046 | 0.647 | 0.067 | 0.408 | 0.076 |
|  | superpathway of pyrimidine deoxyribonucleosides degradation | 1.70E-11 | 0.71 | 0.594 | 0.037 | 0.660 | 0.070 | 0.438 | 0.067 |
|  | CDP-diacylglycerol biosynthesis II | 4.39E-10 | 0.66 | 1.036 | 0.036 | 1.121 | 0.025 | 0.988 | 0.041 |
|  | phosphatidylglycerol biosynthesis I (plastidic) | 7.60E-09 | 0.55 | 0.858 | 0.044 | 0.925 | 0.030 | 0.809 | 0.042 |
|  | phosphatidylglycerol biosynthesis II (non-plastidic) | 7.45E-09 | 0.55 | 0.858 | 0.044 | 0.925 | 0.030 | 0.809 | 0.042 |
|  | tRNA charging | 4.05E-09 | 0.70 | 0.884 | 0.018 | 0.952 | 0.022 | 0.868 | 0.023 |
|  | superpathway of ubiquinol-8 biosynthesis (prokaryotic) | 1.49E-11 | 0.73 | 0.013 | 0.006 | 0.013 | 0.004 | 0.001 | 0.001 |
| Wild type | biotin biosynthesis I | 1.28E-08 | 0.71 | 0.157 | 0.054 | 0.002 | 0.003 | 0.171 | 0.045 |
|  | superpathway of purine nucleotides de novo biosynthesis II | 1.66E-11 | 0.67 | 0.288 | 0.042 | 0.252 | 0.055 | 0.454 | 0.080 |
|  | superpathway of histidine, purine, and pyrimidine biosynthesis | 1.64E-11 | 0.73 | 0.239 | 0.044 | 0.229 | 0.050 | 0.399 | 0.052 |
|  | palmitate biosynthesis II (bacteria and plants) | 1.20E-08 | 0.55 | 0.184 | 0.089 | 0.001 | 0.002 | 0.208 | 0.079 |
|  | superpathway of guanosine nucleotides de novo biosynthesis II | 2.16E-11 | 0.64 | 0.198 | 0.033 | 0.152 | 0.035 | 0.339 | 0.083 |
|  | 8-amino-7-oxononanoate biosynthesis I | 1.48E-08 | 0.64 | 0.154 | 0.062 | 0.002 | 0.003 | 0.163 | 0.051 |
|  | pyrimidine deoxyribonucleotides de novo biosynthesis III | 1.64E-11 | 0.62 | 0.124 | 0.021 | 0.095 | 0.019 | 0.197 | 0.046 |
|  | chondroitin sulfate degradation I (bacterial) | 5.11E-11 | 0.57 | 0.023 | 0.012 | 0.000 | 0.000 | 0.049 | 0.023 |
|  | TCA cycle V (2-oxoglutarate:ferredoxin oxidoreductase) | 5.25E-09 | 0.56 | 0.423 | 0.065 | 0.334 | 0.042 | 0.491 | 0.049 |
|  | pyrimidine deoxyribonucleotides de novo biosynthesis I | 1.95E-11 | 0.59 | 0.132 | 0.022 | 0.105 | 0.021 | 0.236 | 0.068 |
|  | pyrimidine deoxyribonucleotides de novo biosynthesis II | 2.56E-11 | 0.65 | 0.227 | 0.032 | 0.192 | 0.042 | 0.351 | 0.066 |
|  | superpathway of pyrimidine ribonucleosides salvage | 5.10E-11 | 0.65 | 0.194 | 0.033 | 0.145 | 0.034 | 0.346 | 0.088 |
|  | pyrimidine deoxyribonucleotide phosphorylation | 2.24E-11 | 0.58 | 0.119 | 0.021 | 0.092 | 0.023 | 0.221 | 0.069 |
|  | superpathway of pyrimidine deoxyribonucleoside salvage | 1.47E-11 | 0.67 | 0.170 | 0.027 | 0.142 | 0.033 | 0.284 | 0.058 |
|  | superpathway of pyrimidine deoxyribonucleotides de novo biosynthesis | 1.37E-11 | 0.65 | 0.182 | 0.029 | 0.150 | 0.028 | 0.290 | 0.059 |
|  | superpathway of guanosine nucleotides de novo biosynthesis I | 2.79E-11 | 0.62 | 0.174 | 0.029 | 0.132 | 0.031 | 0.305 | 0.080 |
|  | inosine-5’-phosphate biosynthesis III | 9.28E-09 | 0.58 | 0.135 | 0.052 | 0.296 | 0.101 | 0.323 | 0.069 |
|  | myo-, chiro- and scillo-inositol degradation | 1.47E-08 | 0.55 | 0.036 | 0.014 | 0.075 | 0.032 | 0.097 | 0.025 |
|  | superpathway of purine nucleotides de novo biosynthesis I | 3.37E-11 | 0.69 | 0.275 | 0.040 | 0.214 | 0.045 | 0.433 | 0.082 |
|  | superpathway of pyrimidine deoxyribonucleotides de novo biosynthesis (E. coli) | 4.44E-11 | 0.63 | 0.223 | 0.032 | 0.190 | 0.042 | 0.343 | 0.065 |
|  | superpathway of pyridoxal 5’-phosphate biosynthesis and salvage | 6.16E-09 | 0.63 | 0.101 | 0.027 | 0.010 | 0.014 | 0.123 | 0.043 |
|  | mycolate biosynthesis | 1.50E-08 | 0.50 | 0.159 | 0.084 | 0.001 | 0.002 | 0.170 | 0.072 |
|  | pyridoxal 5’-phosphate biosynthesis I | 5.72E-09 | 0.60 | 0.079 | 0.022 | 0.008 | 0.011 | 0.100 | 0.039 |
|  | superpathway of sulfate assimilation and cysteine biosynthesis | 1.64E-10 | 0.55 | 0.073 | 0.041 | 0.051 | 0.023 | 0.179 | 0.067 |
|  | TCA cycle I (prokaryotic) | 4.98E-09 | 0.51 | 0.252 | 0.060 | 0.172 | 0.038 | 0.330 | 0.070 |

**Supplementary Table 4.** Determination of differentially abundant OTUs identified by LEfSe (**Supplementary Table 2**) contributing to predicted activity within glycolysis I and III pathways (**Supplementary Table 3**). Only OTUs classified to genus level are reported.

| **Cluster** | **Genotype** | **Animal ID** | ***Bacteroides* (Otu49)** | ***Alistipes* (Otu11)** | ***Alistipes* (Otu337)** | ***Desulfovibrio* (Otu24)** | ***Odoribacter* (Otu26)** | ***Alistipes* (Otu86)** | ***Lactobacillus* (Otu10)** | ***Lactobacillus* (Otu39)** | ***Bacteroides* (Otu63)** | ***Bacteroides* (Otu81)** | ***Bacteroides* (Otu512)** | ***Odoribacter* (Otu92)** | ***Alistipes* (Otu14)** | ***Alistipes* (Otu67)** | ***Alistipes* (Otu229)** | ***Lactobacillus* (Otu8)** | ***Akkermansia* (Otu6)** | ***Barnesiella* (Otu89)** | ***Prevotella* (Otu29)** | ***Clostridium XlVa* (Otu35)** | ***Ruminococcus2* (Otu106)** | ***Oscillibacter* (Otu70)** | ***Ruminococcus* (Otu66)** | ***Barnesiella* (Otu28)** | ***Parasutterella* (Otu134)** | ***Barnesiella* (Otu196)** | ***Parabacteroides* (Otu165)** | ***Alloprevotella* (Otu21)** |
| --- | --- | --- | --- | --- | --- | --- | --- | --- | --- | --- | --- | --- | --- | --- | --- | --- | --- | --- | --- | --- | --- | --- | --- | --- | --- | --- | --- | --- | --- | --- |
| **Glycolysis I pathway (from glucose 6-phosphate, Embden-Meyerhof-Parnas pathway)** | | | | | | | | | | | | | | | | | | | | | | | | | | | | | | |
| **Th2-deficient** | **IL-33^-/-^** | CS123 | 0.053 | 0.074 | 0.010 | 0.012 | 0.000 | 0.011 | 0.032 | 0.010 | 0.000 | 0.011 | 0.001 | 0.000 | 0.000 | 0.011 | 0.002 | 0.002 | 0.000 | 0.004 | 0.063 | 0.005 | 0.012 | 0.003 | 0.025 | 0.000 | 0.000 | 0.000 | 0.001 | 0.000 |
|  |  | CS124 | 0.021 | 0.052 | 0.011 | 0.015 | 0.000 | 0.005 | 0.006 | 0.003 | 0.000 | 0.010 | 0.001 | 0.000 | 0.000 | 0.009 | 0.000 | 0.003 | 0.000 | 0.007 | 0.034 | 0.005 | 0.006 | 0.005 | 0.028 | 0.000 | 0.000 | 0.000 | 0.000 | 0.000 |
|  |  | CS125 | 0.057 | 0.095 | 0.010 | 0.008 | 0.000 | 0.010 | 0.019 | 0.005 | 0.000 | 0.004 | 0.001 | 0.000 | 0.000 | 0.006 | 0.001 | 0.002 | 0.000 | 0.003 | 0.054 | 0.007 | 0.008 | 0.005 | 0.016 | 0.000 | 0.000 | 0.000 | 0.000 | 0.000 |
|  |  | CS126 | 0.026 | 0.037 | 0.007 | 0.009 | 0.000 | 0.003 | 0.000 | 0.002 | 0.000 | 0.009 | 0.001 | 0.000 | 0.000 | 0.008 | 0.000 | 0.001 | 0.000 | 0.003 | 0.033 | 0.000 | 0.008 | 0.010 | 0.025 | 0.000 | 0.000 | 0.000 | 0.002 | 0.000 |
|  |  | CS127 | 0.033 | 0.038 | 0.007 | 0.002 | 0.000 | 0.010 | 0.000 | 0.000 | 0.000 | 0.027 | 0.001 | 0.000 | 0.000 | 0.010 | 0.000 | 0.002 | 0.000 | 0.002 | 0.065 | 0.000 | 0.006 | 0.007 | 0.019 | 0.000 | 0.000 | 0.000 | 0.001 | 0.000 |
|  |  | CS128 | 0.042 | 0.062 | 0.011 | 0.009 | 0.000 | 0.011 | 0.000 | 0.007 | 0.000 | 0.020 | 0.002 | 0.000 | 0.000 | 0.014 | 0.000 | 0.002 | 0.000 | 0.004 | 0.046 | 0.000 | 0.007 | 0.009 | 0.023 | 0.000 | 0.000 | 0.000 | 0.001 | 0.000 |
|  |  | CS129 | 0.032 | 0.058 | 0.008 | 0.005 | 0.000 | 0.007 | 0.020 | 0.010 | 0.000 | 0.003 | 0.001 | 0.000 | 0.000 | 0.008 | 0.000 | 0.002 | 0.000 | 0.002 | 0.023 | 0.006 | 0.002 | 0.009 | 0.011 | 0.000 | 0.000 | 0.000 | 0.000 | 0.000 |
|  |  | CS130 | 0.043 | 0.079 | 0.016 | 0.017 | 0.000 | 0.008 | 0.034 | 0.012 | 0.000 | 0.004 | 0.001 | 0.000 | 0.000 | 0.007 | 0.001 | 0.002 | 0.000 | 0.006 | 0.017 | 0.004 | 0.001 | 0.006 | 0.020 | 0.000 | 0.000 | 0.000 | 0.002 | 0.000 |
|  |  | CS131 | 0.038 | 0.033 | 0.009 | 0.006 | 0.000 | 0.002 | 0.011 | 0.003 | 0.000 | 0.003 | 0.001 | 0.000 | 0.000 | 0.006 | 0.001 | 0.003 | 0.000 | 0.002 | 0.012 | 0.001 | 0.013 | 0.003 | 0.008 | 0.000 | 0.000 | 0.000 | 0.000 | 0.000 |
|  |  | CS132 | 0.047 | 0.000 | 0.026 | 0.000 | 0.000 | 0.004 | 0.026 | 0.012 | 0.000 | 0.006 | 0.002 | 0.000 | 0.000 | 0.012 | 0.002 | 0.002 | 0.000 | 0.005 | 0.012 | 0.001 | 0.009 | 0.002 | 0.007 | 0.000 | 0.000 | 0.000 | 0.000 | 0.000 |
|  |  | CS133 | 0.032 | 0.062 | 0.011 | 0.006 | 0.000 | 0.004 | 0.002 | 0.001 | 0.000 | 0.002 | 0.001 | 0.000 | 0.000 | 0.008 | 0.000 | 0.006 | 0.000 | 0.007 | 0.028 | 0.000 | 0.001 | 0.007 | 0.009 | 0.000 | 0.000 | 0.001 | 0.001 | 0.000 |
|  |  | CS134 | 0.028 | 0.068 | 0.010 | 0.008 | 0.000 | 0.001 | 0.021 | 0.007 | 0.000 | 0.004 | 0.002 | 0.000 | 0.000 | 0.009 | 0.000 | 0.006 | 0.000 | 0.005 | 0.032 | 0.000 | 0.013 | 0.011 | 0.005 | 0.000 | 0.000 | 0.000 | 0.000 | 0.000 |
|  |  | CS135 | 0.001 | 0.047 | 0.012 | 0.011 | 0.000 | 0.007 | 0.003 | 0.000 | 0.000 | 0.008 | 0.001 | 0.000 | 0.000 | 0.017 | 0.000 | 0.002 | 0.000 | 0.004 | 0.046 | 0.000 | 0.005 | 0.009 | 0.013 | 0.000 | 0.000 | 0.000 | 0.001 | 0.000 |
|  |  | CS136 | 0.038 | 0.078 | 0.010 | 0.011 | 0.000 | 0.002 | 0.015 | 0.010 | 0.000 | 0.006 | 0.003 | 0.000 | 0.000 | 0.003 | 0.000 | 0.002 | 0.000 | 0.003 | 0.024 | 0.000 | 0.009 | 0.011 | 0.005 | 0.000 | 0.000 | 0.000 | 0.001 | 0.000 |
|  | **IL-4Rα^-/-^** | CS138 | 0.011 | 0.086 | 0.015 | 0.008 | 0.011 | 0.003 | 0.034 | 0.009 | 0.004 | 0.016 | 0.002 | 0.000 | 0.004 | 0.000 | 0.005 | 0.002 | 0.000 | 0.003 | 0.006 | 0.009 | 0.005 | 0.001 | 0.012 | 0.000 | 0.000 | 0.001 | 0.001 | 0.000 |
|  |  | CS139 | 0.011 | 0.085 | 0.012 | 0.008 | 0.018 | 0.003 | 0.013 | 0.009 | 0.002 | 0.004 | 0.002 | 0.006 | 0.008 | 0.001 | 0.001 | 0.004 | 0.000 | 0.002 | 0.002 | 0.010 | 0.023 | 0.001 | 0.002 | 0.000 | 0.000 | 0.001 | 0.000 | 0.000 |
|  |  | CS140 | 0.010 | 0.035 | 0.007 | 0.006 | 0.021 | 0.002 | 0.030 | 0.012 | 0.002 | 0.006 | 0.001 | 0.000 | 0.002 | 0.012 | 0.003 | 0.003 | 0.000 | 0.005 | 0.009 | 0.009 | 0.041 | 0.001 | 0.009 | 0.000 | 0.003 | 0.000 | 0.001 | 0.000 |
|  |  | CS141 | 0.003 | 0.065 | 0.016 | 0.011 | 0.024 | 0.002 | 0.031 | 0.019 | 0.004 | 0.002 | 0.002 | 0.008 | 0.001 | 0.003 | 0.001 | 0.009 | 0.000 | 0.004 | 0.004 | 0.027 | 0.017 | 0.000 | 0.003 | 0.000 | 0.001 | 0.001 | 0.001 | 0.000 |
|  |  | CS146 | 0.006 | 0.047 | 0.012 | 0.004 | 0.006 | 0.008 | 0.052 | 0.032 | 0.002 | 0.007 | 0.002 | 0.005 | 0.017 | 0.010 | 0.003 | 0.005 | 0.000 | 0.003 | 0.026 | 0.015 | 0.007 | 0.002 | 0.026 | 0.000 | 0.002 | 0.000 | 0.001 | 0.001 |
|  |  | CS147 | 0.002 | 0.053 | 0.010 | 0.004 | 0.008 | 0.003 | 0.005 | 0.005 | 0.007 | 0.006 | 0.001 | 0.006 | 0.000 | 0.009 | 0.004 | 0.005 | 0.000 | 0.001 | 0.019 | 0.002 | 0.018 | 0.001 | 0.016 | 0.000 | 0.004 | 0.000 | 0.001 | 0.004 |
|  |  | CS148 | 0.004 | 0.050 | 0.007 | 0.006 | 0.006 | 0.008 | 0.000 | 0.000 | 0.011 | 0.004 | 0.002 | 0.008 | 0.000 | 0.016 | 0.003 | 0.019 | 0.000 | 0.001 | 0.054 | 0.020 | 0.008 | 0.008 | 0.031 | 0.000 | 0.006 | 0.001 | 0.001 | 0.004 |
|  |  | CS149 | 0.002 | 0.056 | 0.018 | 0.006 | 0.033 | 0.003 | 0.031 | 0.021 | 0.005 | 0.009 | 0.002 | 0.007 | 0.001 | 0.005 | 0.006 | 0.011 | 0.000 | 0.004 | 0.018 | 0.012 | 0.012 | 0.004 | 0.008 | 0.000 | 0.001 | 0.000 | 0.000 | 0.005 |
|  |  | CS150 | 0.010 | 0.042 | 0.015 | 0.004 | 0.003 | 0.005 | 0.000 | 0.000 | 0.010 | 0.005 | 0.001 | 0.008 | 0.015 | 0.024 | 0.012 | 0.003 | 0.000 | 0.004 | 0.025 | 0.000 | 0.007 | 0.002 | 0.023 | 0.000 | 0.007 | 0.000 | 0.002 | 0.004 |
|  |  | CS151 | 0.010 | 0.034 | 0.018 | 0.006 | 0.016 | 0.011 | 0.001 | 0.000 | 0.004 | 0.007 | 0.002 | 0.007 | 0.022 | 0.015 | 0.007 | 0.006 | 0.000 | 0.005 | 0.008 | 0.018 | 0.001 | 0.003 | 0.021 | 0.000 | 0.002 | 0.001 | 0.001 | 0.002 |
|  |  | CS152 | 0.010 | 0.035 | 0.014 | 0.016 | 0.014 | 0.000 | 0.012 | 0.001 | 0.001 | 0.007 | 0.002 | 0.001 | 0.009 | 0.004 | 0.002 | 0.004 | 0.000 | 0.004 | 0.009 | 0.003 | 0.018 | 0.001 | 0.006 | 0.000 | 0.001 | 0.000 | 0.001 | 0.000 |
|  |  | CS153 | 0.018 | 0.050 | 0.012 | 0.008 | 0.010 | 0.005 | 0.000 | 0.000 | 0.001 | 0.003 | 0.001 | 0.011 | 0.056 | 0.011 | 0.006 | 0.003 | 0.000 | 0.004 | 0.022 | 0.014 | 0.008 | 0.001 | 0.018 | 0.000 | 0.003 | 0.001 | 0.001 | 0.002 |
| **Th1/Th2-deficient** | **NSG** | CS154 | 0.000 | 0.025 | 0.007 | 0.007 | 0.017 | 0.011 | 0.061 | 0.017 | 0.000 | 0.000 | 0.000 | 0.000 | 0.032 | 0.000 | 0.000 | 0.000 | 0.009 | 0.002 | 0.000 | 0.000 | 0.012 | 0.003 | 0.002 | 0.003 | 0.005 | 0.000 | 0.000 | 0.000 |
|  |  | CS155 | 0.000 | 0.089 | 0.009 | 0.007 | 0.025 | 0.008 | 0.004 | 0.001 | 0.000 | 0.000 | 0.000 | 0.000 | 0.000 | 0.000 | 0.000 | 0.000 | 0.008 | 0.001 | 0.000 | 0.000 | 0.013 | 0.008 | 0.000 | 0.002 | 0.000 | 0.000 | 0.000 | 0.000 |
|  |  | CS156 | 0.000 | 0.044 | 0.007 | 0.005 | 0.015 | 0.012 | 0.065 | 0.025 | 0.000 | 0.000 | 0.000 | 0.000 | 0.011 | 0.000 | 0.000 | 0.000 | 0.005 | 0.002 | 0.000 | 0.000 | 0.002 | 0.006 | 0.002 | 0.006 | 0.003 | 0.000 | 0.000 | 0.000 |
|  |  | CS157 | 0.000 | 0.100 | 0.009 | 0.008 | 0.021 | 0.013 | 0.023 | 0.006 | 0.000 | 0.000 | 0.000 | 0.000 | 0.000 | 0.000 | 0.000 | 0.000 | 0.010 | 0.000 | 0.000 | 0.000 | 0.019 | 0.007 | 0.002 | 0.002 | 0.000 | 0.000 | 0.000 | 0.000 |
|  |  | CS178 | 0.000 | 0.036 | 0.010 | 0.008 | 0.007 | 0.012 | 0.105 | 0.036 | 0.000 | 0.000 | 0.000 | 0.000 | 0.035 | 0.000 | 0.000 | 0.000 | 0.008 | 0.001 | 0.000 | 0.000 | 0.006 | 0.003 | 0.002 | 0.001 | 0.011 | 0.000 | 0.000 | 0.000 |
|  |  | CS179 | 0.000 | 0.126 | 0.011 | 0.014 | 0.016 | 0.013 | 0.104 | 0.039 | 0.000 | 0.000 | 0.000 | 0.000 | 0.035 | 0.000 | 0.000 | 0.000 | 0.000 | 0.000 | 0.000 | 0.000 | 0.009 | 0.004 | 0.001 | 0.011 | 0.006 | 0.000 | 0.000 | 0.000 |
|  |  | CS180 | 0.000 | 0.050 | 0.007 | 0.009 | 0.013 | 0.006 | 0.151 | 0.039 | 0.000 | 0.000 | 0.000 | 0.000 | 0.000 | 0.000 | 0.000 | 0.000 | 0.000 | 0.001 | 0.000 | 0.000 | 0.003 | 0.006 | 0.001 | 0.007 | 0.011 | 0.000 | 0.000 | 0.000 |
|  |  | CS193 | 0.000 | 0.027 | 0.007 | 0.007 | 0.011 | 0.009 | 0.114 | 0.026 | 0.000 | 0.000 | 0.000 | 0.000 | 0.073 | 0.000 | 0.000 | 0.000 | 0.000 | 0.001 | 0.000 | 0.000 | 0.001 | 0.001 | 0.003 | 0.000 | 0.002 | 0.000 | 0.000 | 0.000 |
|  |  | CS194 | 0.000 | 0.032 | 0.011 | 0.006 | 0.003 | 0.020 | 0.167 | 0.054 | 0.000 | 0.000 | 0.000 | 0.000 | 0.060 | 0.000 | 0.000 | 0.000 | 0.000 | 0.001 | 0.000 | 0.000 | 0.001 | 0.002 | 0.001 | 0.002 | 0.004 | 0.000 | 0.000 | 0.000 |
|  |  | CS195 | 0.000 | 0.043 | 0.004 | 0.007 | 0.005 | 0.008 | 0.125 | 0.033 | 0.000 | 0.000 | 0.000 | 0.000 | 0.044 | 0.000 | 0.000 | 0.000 | 0.000 | 0.001 | 0.000 | 0.000 | 0.007 | 0.001 | 0.001 | 0.000 | 0.002 | 0.000 | 0.000 | 0.000 |
|  |  | CS196 | 0.000 | 0.042 | 0.008 | 0.003 | 0.003 | 0.020 | 0.202 | 0.051 | 0.000 | 0.000 | 0.000 | 0.000 | 0.063 | 0.000 | 0.000 | 0.000 | 0.000 | 0.002 | 0.000 | 0.000 | 0.000 | 0.008 | 0.002 | 0.006 | 0.009 | 0.000 | 0.000 | 0.000 |
|  |  | CS197 | 0.000 | 0.032 | 0.009 | 0.007 | 0.012 | 0.016 | 0.123 | 0.045 | 0.000 | 0.000 | 0.000 | 0.000 | 0.060 | 0.000 | 0.000 | 0.000 | 0.000 | 0.001 | 0.000 | 0.000 | 0.001 | 0.001 | 0.004 | 0.000 | 0.002 | 0.000 | 0.000 | 0.000 |
|  |  | CS198 | 0.000 | 0.039 | 0.005 | 0.008 | 0.006 | 0.011 | 0.056 | 0.024 | 0.000 | 0.000 | 0.000 | 0.000 | 0.067 | 0.000 | 0.000 | 0.000 | 0.000 | 0.000 | 0.000 | 0.000 | 0.015 | 0.001 | 0.001 | 0.002 | 0.003 | 0.000 | 0.000 | 0.000 |
|  |  | CS199 | 0.000 | 0.069 | 0.009 | 0.004 | 0.013 | 0.025 | 0.097 | 0.031 | 0.000 | 0.000 | 0.000 | 0.000 | 0.046 | 0.000 | 0.000 | 0.000 | 0.000 | 0.001 | 0.000 | 0.000 | 0.000 | 0.002 | 0.004 | 0.000 | 0.005 | 0.000 | 0.000 | 0.000 |
|  |  | CS200 | 0.000 | 0.028 | 0.009 | 0.005 | 0.011 | 0.014 | 0.077 | 0.023 | 0.000 | 0.000 | 0.000 | 0.000 | 0.076 | 0.000 | 0.000 | 0.000 | 0.000 | 0.001 | 0.000 | 0.000 | 0.000 | 0.001 | 0.002 | 0.000 | 0.004 | 0.000 | 0.000 | 0.000 |
|  |  | CS201 | 0.000 | 0.045 | 0.012 | 0.008 | 0.010 | 0.032 | 0.116 | 0.033 | 0.000 | 0.000 | 0.000 | 0.000 | 0.069 | 0.000 | 0.000 | 0.000 | 0.000 | 0.001 | 0.000 | 0.000 | 0.000 | 0.002 | 0.010 | 0.000 | 0.009 | 0.000 | 0.000 | 0.000 |
|  |  | CS202 | 0.000 | 0.059 | 0.007 | 0.006 | 0.006 | 0.016 | 0.094 | 0.032 | 0.000 | 0.000 | 0.000 | 0.000 | 0.080 | 0.000 | 0.000 | 0.000 | 0.000 | 0.000 | 0.000 | 0.000 | 0.001 | 0.003 | 0.005 | 0.004 | 0.004 | 0.000 | 0.000 | 0.000 |
| **Wild type** | **BALB/c** | CS162 | 0.034 | 0.059 | 0.001 | 0.000 | 0.011 | 0.007 | 0.010 | 0.003 | 0.006 | 0.003 | 0.002 | 0.009 | 0.085 | 0.005 | 0.004 | 0.009 | 0.000 | 0.001 | 0.012 | 0.002 | 0.000 | 0.004 | 0.000 | 0.000 | 0.001 | 0.001 | 0.000 | 0.006 |
|  |  | CS163 | 0.020 | 0.048 | 0.000 | 0.000 | 0.011 | 0.001 | 0.034 | 0.022 | 0.013 | 0.007 | 0.002 | 0.007 | 0.073 | 0.008 | 0.001 | 0.003 | 0.000 | 0.002 | 0.015 | 0.000 | 0.004 | 0.002 | 0.000 | 0.000 | 0.001 | 0.001 | 0.000 | 0.001 |
|  |  | CS164 | 0.014 | 0.069 | 0.000 | 0.000 | 0.013 | 0.004 | 0.033 | 0.014 | 0.005 | 0.002 | 0.003 | 0.007 | 0.092 | 0.004 | 0.004 | 0.006 | 0.000 | 0.006 | 0.003 | 0.006 | 0.004 | 0.002 | 0.000 | 0.000 | 0.001 | 0.001 | 0.000 | 0.039 |
|  |  | CS165 | 0.021 | 0.028 | 0.000 | 0.000 | 0.017 | 0.008 | 0.009 | 0.007 | 0.020 | 0.002 | 0.002 | 0.011 | 0.061 | 0.007 | 0.000 | 0.003 | 0.000 | 0.001 | 0.018 | 0.000 | 0.001 | 0.000 | 0.001 | 0.000 | 0.000 | 0.001 | 0.001 | 0.004 |
|  |  | CS166 | 0.012 | 0.042 | 0.001 | 0.000 | 0.008 | 0.001 | 0.013 | 0.021 | 0.017 | 0.001 | 0.001 | 0.006 | 0.054 | 0.002 | 0.000 | 0.002 | 0.000 | 0.001 | 0.019 | 0.006 | 0.017 | 0.000 | 0.002 | 0.000 | 0.001 | 0.001 | 0.001 | 0.009 |
|  |  | CS167 | 0.018 | 0.026 | 0.000 | 0.000 | 0.012 | 0.008 | 0.035 | 0.020 | 0.009 | 0.003 | 0.001 | 0.005 | 0.054 | 0.002 | 0.002 | 0.006 | 0.000 | 0.002 | 0.027 | 0.012 | 0.003 | 0.001 | 0.003 | 0.000 | 0.001 | 0.001 | 0.001 | 0.019 |
|  |  | CS168 | 0.033 | 0.043 | 0.004 | 0.000 | 0.016 | 0.010 | 0.006 | 0.004 | 0.022 | 0.004 | 0.001 | 0.007 | 0.074 | 0.006 | 0.004 | 0.002 | 0.000 | 0.001 | 0.037 | 0.003 | 0.005 | 0.001 | 0.003 | 0.000 | 0.000 | 0.001 | 0.001 | 0.013 |
|  |  | CS169 | 0.026 | 0.061 | 0.018 | 0.000 | 0.022 | 0.005 | 0.050 | 0.036 | 0.010 | 0.001 | 0.006 | 0.007 | 0.000 | 0.004 | 0.006 | 0.003 | 0.000 | 0.003 | 0.003 | 0.000 | 0.001 | 0.005 | 0.002 | 0.000 | 0.003 | 0.002 | 0.000 | 0.007 |
|  |  | CS170 | 0.058 | 0.029 | 0.000 | 0.000 | 0.001 | 0.002 | 0.050 | 0.044 | 0.007 | 0.001 | 0.002 | 0.006 | 0.038 | 0.007 | 0.014 | 0.011 | 0.000 | 0.005 | 0.043 | 0.000 | 0.017 | 0.003 | 0.014 | 0.000 | 0.003 | 0.001 | 0.000 | 0.001 |
|  |  | CS171 | 0.041 | 0.035 | 0.000 | 0.000 | 0.002 | 0.000 | 0.008 | 0.002 | 0.004 | 0.006 | 0.004 | 0.004 | 0.070 | 0.008 | 0.006 | 0.067 | 0.000 | 0.004 | 0.053 | 0.000 | 0.006 | 0.005 | 0.001 | 0.000 | 0.003 | 0.001 | 0.001 | 0.032 |
|  |  | CS172 | 0.037 | 0.028 | 0.000 | 0.000 | 0.006 | 0.001 | 0.000 | 0.000 | 0.006 | 0.005 | 0.002 | 0.004 | 0.071 | 0.004 | 0.005 | 0.008 | 0.000 | 0.003 | 0.064 | 0.000 | 0.000 | 0.000 | 0.001 | 0.000 | 0.003 | 0.001 | 0.000 | 0.014 |
|  |  | CS173 | 0.020 | 0.033 | 0.000 | 0.000 | 0.001 | 0.000 | 0.000 | 0.000 | 0.008 | 0.002 | 0.002 | 0.006 | 0.095 | 0.002 | 0.002 | 0.036 | 0.000 | 0.003 | 0.013 | 0.000 | 0.018 | 0.012 | 0.002 | 0.000 | 0.001 | 0.001 | 0.000 | 0.005 |
|  |  | CS174 | 0.037 | 0.025 | 0.000 | 0.000 | 0.005 | 0.017 | 0.019 | 0.008 | 0.027 | 0.006 | 0.003 | 0.005 | 0.054 | 0.036 | 0.007 | 0.014 | 0.000 | 0.002 | 0.014 | 0.000 | 0.000 | 0.001 | 0.009 | 0.000 | 0.001 | 0.001 | 0.002 | 0.017 |
|  |  | CS175 | 0.041 | 0.030 | 0.000 | 0.000 | 0.013 | 0.016 | 0.000 | 0.001 | 0.025 | 0.003 | 0.003 | 0.004 | 0.071 | 0.002 | 0.009 | 0.017 | 0.000 | 0.001 | 0.001 | 0.000 | 0.000 | 0.000 | 0.002 | 0.000 | 0.001 | 0.001 | 0.001 | 0.020 |
|  |  | CS176 | 0.029 | 0.035 | 0.000 | 0.000 | 0.003 | 0.019 | 0.049 | 0.060 | 0.013 | 0.003 | 0.004 | 0.008 | 0.088 | 0.015 | 0.003 | 0.012 | 0.000 | 0.003 | 0.001 | 0.000 | 0.000 | 0.001 | 0.017 | 0.000 | 0.003 | 0.001 | 0.001 | 0.001 |
|  |  | CS177 | 0.016 | 0.023 | 0.000 | 0.000 | 0.007 | 0.004 | 0.075 | 0.055 | 0.006 | 0.021 | 0.004 | 0.005 | 0.065 | 0.006 | 0.004 | 0.006 | 0.000 | 0.003 | 0.003 | 0.000 | 0.007 | 0.002 | 0.000 | 0.000 | 0.002 | 0.000 | 0.001 | 0.003 |
|  | **C57BL/6** | CS203 | 0.006 | 0.000 | 0.009 | 0.000 | 0.001 | 0.013 | 0.033 | 0.045 | 0.007 | 0.028 | 0.001 | 0.002 | 0.005 | 0.043 | 0.007 | 0.004 | 0.098 | 0.000 | 0.005 | 0.000 | 0.000 | 0.003 | 0.000 | 0.000 | 0.015 | 0.001 | 0.007 | 0.084 |
|  |  | CS204 | 0.008 | 0.003 | 0.011 | 0.000 | 0.003 | 0.022 | 0.049 | 0.017 | 0.009 | 0.037 | 0.001 | 0.004 | 0.010 | 0.041 | 0.005 | 0.001 | 0.088 | 0.001 | 0.005 | 0.000 | 0.000 | 0.001 | 0.000 | 0.000 | 0.004 | 0.000 | 0.004 | 0.038 |
|  |  | CS205 | 0.008 | 0.011 | 0.007 | 0.000 | 0.005 | 0.003 | 0.154 | 0.081 | 0.004 | 0.011 | 0.002 | 0.005 | 0.045 | 0.012 | 0.000 | 0.007 | 0.000 | 0.007 | 0.021 | 0.000 | 0.000 | 0.000 | 0.001 | 0.000 | 0.005 | 0.001 | 0.000 | 0.016 |
|  |  | CS206 | 0.011 | 0.013 | 0.011 | 0.000 | 0.004 | 0.007 | 0.120 | 0.047 | 0.008 | 0.038 | 0.003 | 0.005 | 0.054 | 0.013 | 0.001 | 0.004 | 0.012 | 0.004 | 0.012 | 0.000 | 0.000 | 0.001 | 0.001 | 0.000 | 0.006 | 0.001 | 0.001 | 0.006 |
|  |  | CS207 | 0.002 | 0.002 | 0.001 | 0.000 | 0.002 | 0.008 | 0.140 | 0.084 | 0.001 | 0.005 | 0.002 | 0.001 | 0.033 | 0.009 | 0.001 | 0.034 | 0.009 | 0.002 | 0.017 | 0.000 | 0.003 | 0.000 | 0.003 | 0.000 | 0.003 | 0.000 | 0.000 | 0.046 |
|  |  | CS208 | 0.010 | 0.006 | 0.015 | 0.000 | 0.020 | 0.008 | 0.103 | 0.036 | 0.011 | 0.006 | 0.005 | 0.008 | 0.049 | 0.018 | 0.001 | 0.006 | 0.001 | 0.004 | 0.020 | 0.000 | 0.000 | 0.000 | 0.012 | 0.000 | 0.008 | 0.001 | 0.002 | 0.038 |
|  |  | CS209 | 0.003 | 0.002 | 0.005 | 0.000 | 0.004 | 0.012 | 0.013 | 0.011 | 0.001 | 0.004 | 0.002 | 0.004 | 0.037 | 0.024 | 0.009 | 0.040 | 0.031 | 0.002 | 0.039 | 0.000 | 0.000 | 0.000 | 0.025 | 0.000 | 0.005 | 0.000 | 0.001 | 0.052 |
|  |  | CS210 | 0.004 | 0.004 | 0.011 | 0.000 | 0.021 | 0.016 | 0.002 | 0.000 | 0.007 | 0.003 | 0.003 | 0.006 | 0.067 | 0.014 | 0.000 | 0.027 | 0.000 | 0.001 | 0.029 | 0.000 | 0.000 | 0.000 | 0.006 | 0.000 | 0.007 | 0.001 | 0.002 | 0.010 |
|  |  | CS211 | 0.003 | 0.008 | 0.004 | 0.000 | 0.005 | 0.011 | 0.091 | 0.070 | 0.001 | 0.013 | 0.002 | 0.004 | 0.024 | 0.023 | 0.004 | 0.016 | 0.022 | 0.001 | 0.029 | 0.000 | 0.000 | 0.000 | 0.014 | 0.000 | 0.009 | 0.000 | 0.002 | 0.054 |
|  |  | CS212 | 0.002 | 0.007 | 0.008 | 0.000 | 0.004 | 0.005 | 0.174 | 0.061 | 0.004 | 0.016 | 0.005 | 0.002 | 0.023 | 0.005 | 0.000 | 0.007 | 0.005 | 0.005 | 0.013 | 0.000 | 0.000 | 0.000 | 0.013 | 0.000 | 0.008 | 0.000 | 0.001 | 0.046 |
|  |  | CS213 | 0.000 | 0.002 | 0.006 | 0.000 | 0.013 | 0.026 | 0.063 | 0.055 | 0.003 | 0.011 | 0.003 | 0.000 | 0.070 | 0.023 | 0.002 | 0.010 | 0.010 | 0.003 | 0.026 | 0.000 | 0.000 | 0.001 | 0.009 | 0.000 | 0.011 | 0.000 | 0.001 | 0.089 |
|  |  | CS214 | 0.003 | 0.012 | 0.014 | 0.000 | 0.006 | 0.022 | 0.004 | 0.001 | 0.008 | 0.012 | 0.001 | 0.005 | 0.052 | 0.022 | 0.000 | 0.004 | 0.014 | 0.002 | 0.022 | 0.000 | 0.000 | 0.001 | 0.003 | 0.000 | 0.002 | 0.001 | 0.002 | 0.019 |
|  |  | CS215 | 0.005 | 0.002 | 0.008 | 0.000 | 0.005 | 0.010 | 0.079 | 0.040 | 0.006 | 0.021 | 0.003 | 0.000 | 0.034 | 0.015 | 0.002 | 0.008 | 0.001 | 0.005 | 0.025 | 0.000 | 0.000 | 0.001 | 0.001 | 0.000 | 0.008 | 0.001 | 0.002 | 0.022 |
|  |  | CS216 | 0.006 | 0.043 | 0.009 | 0.000 | 0.007 | 0.012 | 0.007 | 0.007 | 0.026 | 0.014 | 0.002 | 0.007 | 0.033 | 0.013 | 0.001 | 0.005 | 0.006 | 0.001 | 0.030 | 0.000 | 0.000 | 0.002 | 0.000 | 0.000 | 0.005 | 0.002 | 0.001 | 0.006 |
|  |  | CS217 | 0.014 | 0.001 | 0.003 | 0.000 | 0.007 | 0.023 | 0.075 | 0.031 | 0.012 | 0.007 | 0.002 | 0.000 | 0.049 | 0.018 | 0.003 | 0.009 | 0.002 | 0.002 | 0.021 | 0.000 | 0.008 | 0.000 | 0.006 | 0.000 | 0.008 | 0.001 | 0.001 | 0.033 |
|  |  | CS218 | 0.002 | 0.009 | 0.006 | 0.000 | 0.011 | 0.014 | 0.002 | 0.001 | 0.007 | 0.019 | 0.002 | 0.002 | 0.040 | 0.012 | 0.002 | 0.006 | 0.011 | 0.002 | 0.030 | 0.000 | 0.006 | 0.001 | 0.005 | 0.000 | 0.004 | 0.001 | 0.002 | 0.003 |
|  |  | CS219 | 0.018 | 0.006 | 0.008 | 0.000 | 0.005 | 0.022 | 0.058 | 0.032 | 0.008 | 0.003 | 0.003 | 0.000 | 0.082 | 0.010 | 0.003 | 0.004 | 0.000 | 0.003 | 0.021 | 0.000 | 0.000 | 0.002 | 0.000 | 0.000 | 0.004 | 0.002 | 0.000 | 0.024 |
|  |  | CS220 | 0.004 | 0.006 | 0.007 | 0.000 | 0.014 | 0.007 | 0.000 | 0.003 | 0.024 | 0.013 | 0.001 | 0.003 | 0.064 | 0.010 | 0.002 | 0.011 | 0.002 | 0.001 | 0.025 | 0.000 | 0.000 | 0.003 | 0.000 | 0.000 | 0.002 | 0.001 | 0.001 | 0.004 |
| **Glycolysis III pathway (from glucose, Embden-Meyerhof-Parnas pathway)** | | | | | | | | | | | | | | | | | | | | | | | | | | | | | | |
| **Th2-deficient** | **IL-33^-/-^** | CS123 | 0.058 | 0.086 | 0.012 | 0.009 | 0.000 | 0.014 | 0.029 | 0.008 | 0.000 | 0.013 | 0.001 | 0.000 | 0.000 | 0.015 | 0.002 | 0.002 | 0.000 | 0.004 | 0.057 | 0.005 | 0.012 | 0.003 | 0.027 | 0.000 | 0.000 | 0.000 | 0.001 | 0.000 |
|  |  | CS124 | 0.023 | 0.062 | 0.013 | 0.011 | 0.000 | 0.007 | 0.006 | 0.002 | 0.000 | 0.012 | 0.002 | 0.000 | 0.000 | 0.012 | 0.000 | 0.002 | 0.000 | 0.007 | 0.031 | 0.005 | 0.005 | 0.005 | 0.031 | 0.000 | 0.000 | 0.000 | 0.001 | 0.000 |
|  |  | CS125 | 0.062 | 0.111 | 0.012 | 0.006 | 0.000 | 0.013 | 0.017 | 0.004 | 0.000 | 0.005 | 0.001 | 0.000 | 0.000 | 0.007 | 0.001 | 0.001 | 0.000 | 0.003 | 0.049 | 0.006 | 0.007 | 0.005 | 0.017 | 0.000 | 0.000 | 0.000 | 0.001 | 0.000 |
|  |  | CS126 | 0.029 | 0.044 | 0.008 | 0.007 | 0.000 | 0.004 | 0.000 | 0.002 | 0.000 | 0.010 | 0.001 | 0.000 | 0.000 | 0.010 | 0.000 | 0.001 | 0.000 | 0.003 | 0.030 | 0.000 | 0.008 | 0.010 | 0.027 | 0.000 | 0.000 | 0.000 | 0.002 | 0.000 |
|  |  | CS127 | 0.037 | 0.045 | 0.009 | 0.002 | 0.000 | 0.013 | 0.000 | 0.000 | 0.000 | 0.031 | 0.001 | 0.000 | 0.000 | 0.012 | 0.000 | 0.002 | 0.000 | 0.002 | 0.059 | 0.000 | 0.006 | 0.007 | 0.021 | 0.000 | 0.000 | 0.000 | 0.001 | 0.000 |
|  |  | CS128 | 0.046 | 0.073 | 0.013 | 0.007 | 0.000 | 0.014 | 0.000 | 0.006 | 0.000 | 0.024 | 0.002 | 0.000 | 0.000 | 0.017 | 0.000 | 0.002 | 0.000 | 0.004 | 0.042 | 0.000 | 0.006 | 0.008 | 0.025 | 0.000 | 0.000 | 0.000 | 0.001 | 0.000 |
|  |  | CS129 | 0.035 | 0.069 | 0.009 | 0.004 | 0.000 | 0.009 | 0.018 | 0.008 | 0.000 | 0.004 | 0.002 | 0.000 | 0.000 | 0.011 | 0.000 | 0.001 | 0.000 | 0.002 | 0.021 | 0.005 | 0.002 | 0.009 | 0.012 | 0.000 | 0.000 | 0.001 | 0.001 | 0.000 |
|  |  | CS130 | 0.048 | 0.093 | 0.019 | 0.013 | 0.000 | 0.010 | 0.030 | 0.010 | 0.000 | 0.005 | 0.002 | 0.000 | 0.000 | 0.009 | 0.001 | 0.001 | 0.000 | 0.006 | 0.016 | 0.003 | 0.001 | 0.006 | 0.021 | 0.000 | 0.000 | 0.000 | 0.002 | 0.000 |
|  |  | CS131 | 0.042 | 0.039 | 0.011 | 0.005 | 0.000 | 0.003 | 0.010 | 0.003 | 0.000 | 0.004 | 0.001 | 0.000 | 0.000 | 0.007 | 0.001 | 0.002 | 0.000 | 0.002 | 0.011 | 0.001 | 0.012 | 0.003 | 0.009 | 0.000 | 0.000 | 0.000 | 0.000 | 0.000 |
|  |  | CS132 | 0.052 | 0.000 | 0.030 | 0.000 | 0.000 | 0.005 | 0.024 | 0.010 | 0.000 | 0.007 | 0.002 | 0.000 | 0.000 | 0.016 | 0.002 | 0.002 | 0.000 | 0.005 | 0.010 | 0.001 | 0.009 | 0.002 | 0.008 | 0.000 | 0.000 | 0.000 | 0.000 | 0.000 |
|  |  | CS133 | 0.036 | 0.074 | 0.013 | 0.005 | 0.000 | 0.005 | 0.002 | 0.001 | 0.000 | 0.002 | 0.001 | 0.000 | 0.000 | 0.010 | 0.000 | 0.005 | 0.000 | 0.008 | 0.025 | 0.000 | 0.001 | 0.006 | 0.009 | 0.000 | 0.000 | 0.001 | 0.001 | 0.000 |
|  |  | CS134 | 0.031 | 0.081 | 0.011 | 0.006 | 0.000 | 0.002 | 0.019 | 0.006 | 0.000 | 0.004 | 0.002 | 0.000 | 0.000 | 0.012 | 0.000 | 0.005 | 0.000 | 0.006 | 0.029 | 0.000 | 0.012 | 0.010 | 0.005 | 0.000 | 0.000 | 0.000 | 0.000 | 0.000 |
|  |  | CS135 | 0.001 | 0.056 | 0.014 | 0.008 | 0.000 | 0.009 | 0.002 | 0.000 | 0.000 | 0.009 | 0.002 | 0.000 | 0.000 | 0.022 | 0.000 | 0.002 | 0.000 | 0.005 | 0.042 | 0.000 | 0.005 | 0.009 | 0.014 | 0.000 | 0.000 | 0.001 | 0.001 | 0.000 |
|  |  | CS136 | 0.042 | 0.093 | 0.012 | 0.008 | 0.000 | 0.003 | 0.014 | 0.009 | 0.000 | 0.007 | 0.003 | 0.000 | 0.000 | 0.003 | 0.000 | 0.002 | 0.000 | 0.003 | 0.022 | 0.000 | 0.009 | 0.011 | 0.005 | 0.000 | 0.000 | 0.001 | 0.001 | 0.000 |
|  | **IL-4Rα^-/-^** | CS138 | 0.012 | 0.102 | 0.017 | 0.006 | 0.012 | 0.004 | 0.031 | 0.007 | 0.005 | 0.019 | 0.002 | 0.000 | 0.005 | 0.000 | 0.006 | 0.002 | 0.000 | 0.003 | 0.006 | 0.008 | 0.005 | 0.001 | 0.013 | 0.000 | 0.000 | 0.001 | 0.001 | 0.000 |
|  |  | CS139 | 0.013 | 0.100 | 0.015 | 0.006 | 0.020 | 0.004 | 0.011 | 0.007 | 0.003 | 0.005 | 0.002 | 0.007 | 0.011 | 0.002 | 0.001 | 0.003 | 0.000 | 0.002 | 0.002 | 0.009 | 0.022 | 0.001 | 0.002 | 0.000 | 0.000 | 0.001 | 0.001 | 0.000 |
|  |  | CS140 | 0.012 | 0.041 | 0.009 | 0.005 | 0.023 | 0.003 | 0.027 | 0.010 | 0.003 | 0.007 | 0.002 | 0.000 | 0.003 | 0.016 | 0.004 | 0.003 | 0.000 | 0.005 | 0.008 | 0.008 | 0.038 | 0.001 | 0.010 | 0.000 | 0.003 | 0.001 | 0.001 | 0.000 |
|  |  | CS141 | 0.003 | 0.077 | 0.019 | 0.008 | 0.027 | 0.002 | 0.028 | 0.016 | 0.005 | 0.003 | 0.002 | 0.009 | 0.002 | 0.004 | 0.001 | 0.007 | 0.000 | 0.005 | 0.003 | 0.025 | 0.016 | 0.000 | 0.004 | 0.000 | 0.001 | 0.001 | 0.001 | 0.000 |
|  |  | CS146 | 0.007 | 0.056 | 0.014 | 0.003 | 0.006 | 0.010 | 0.047 | 0.027 | 0.002 | 0.008 | 0.003 | 0.005 | 0.023 | 0.013 | 0.004 | 0.004 | 0.000 | 0.003 | 0.024 | 0.014 | 0.007 | 0.002 | 0.029 | 0.000 | 0.001 | 0.000 | 0.001 | 0.001 |
|  |  | CS147 | 0.002 | 0.063 | 0.012 | 0.003 | 0.009 | 0.004 | 0.004 | 0.004 | 0.009 | 0.007 | 0.001 | 0.007 | 0.000 | 0.011 | 0.005 | 0.004 | 0.000 | 0.001 | 0.017 | 0.002 | 0.016 | 0.001 | 0.017 | 0.000 | 0.004 | 0.000 | 0.001 | 0.004 |
|  |  | CS148 | 0.005 | 0.060 | 0.008 | 0.004 | 0.007 | 0.011 | 0.000 | 0.000 | 0.014 | 0.004 | 0.002 | 0.009 | 0.001 | 0.020 | 0.004 | 0.014 | 0.000 | 0.001 | 0.049 | 0.019 | 0.007 | 0.007 | 0.033 | 0.000 | 0.006 | 0.001 | 0.001 | 0.004 |
|  |  | CS149 | 0.002 | 0.066 | 0.021 | 0.005 | 0.036 | 0.004 | 0.028 | 0.017 | 0.007 | 0.010 | 0.002 | 0.007 | 0.002 | 0.006 | 0.007 | 0.008 | 0.000 | 0.004 | 0.017 | 0.011 | 0.011 | 0.004 | 0.009 | 0.000 | 0.001 | 0.000 | 0.001 | 0.005 |
|  |  | CS150 | 0.011 | 0.049 | 0.017 | 0.003 | 0.003 | 0.006 | 0.000 | 0.000 | 0.012 | 0.006 | 0.002 | 0.008 | 0.021 | 0.030 | 0.014 | 0.002 | 0.000 | 0.004 | 0.022 | 0.000 | 0.006 | 0.002 | 0.025 | 0.000 | 0.006 | 0.001 | 0.002 | 0.003 |
|  |  | CS151 | 0.011 | 0.040 | 0.021 | 0.005 | 0.018 | 0.014 | 0.001 | 0.000 | 0.006 | 0.008 | 0.002 | 0.008 | 0.030 | 0.019 | 0.008 | 0.004 | 0.000 | 0.006 | 0.007 | 0.016 | 0.001 | 0.003 | 0.022 | 0.000 | 0.002 | 0.001 | 0.001 | 0.002 |
|  |  | CS152 | 0.011 | 0.042 | 0.017 | 0.012 | 0.016 | 0.000 | 0.011 | 0.001 | 0.001 | 0.009 | 0.002 | 0.002 | 0.013 | 0.005 | 0.002 | 0.003 | 0.000 | 0.004 | 0.008 | 0.003 | 0.017 | 0.001 | 0.006 | 0.000 | 0.001 | 0.000 | 0.001 | 0.000 |
|  |  | CS153 | 0.020 | 0.057 | 0.013 | 0.006 | 0.011 | 0.006 | 0.000 | 0.000 | 0.001 | 0.004 | 0.001 | 0.011 | 0.075 | 0.014 | 0.007 | 0.002 | 0.000 | 0.005 | 0.019 | 0.013 | 0.008 | 0.001 | 0.019 | 0.000 | 0.002 | 0.001 | 0.001 | 0.002 |
| **Th1/Th2-deficient** | **NSG** | CS154 | 0.000 | 0.029 | 0.008 | 0.005 | 0.018 | 0.014 | 0.055 | 0.014 | 0.000 | 0.000 | 0.000 | 0.000 | 0.043 | 0.000 | 0.000 | 0.000 | 0.011 | 0.002 | 0.000 | 0.000 | 0.011 | 0.003 | 0.002 | 0.003 | 0.005 | 0.000 | 0.000 | 0.000 |
|  |  | CS155 | 0.000 | 0.102 | 0.011 | 0.005 | 0.027 | 0.010 | 0.004 | 0.001 | 0.000 | 0.000 | 0.000 | 0.000 | 0.000 | 0.000 | 0.000 | 0.000 | 0.010 | 0.001 | 0.000 | 0.000 | 0.012 | 0.007 | 0.000 | 0.003 | 0.000 | 0.000 | 0.000 | 0.000 |
|  |  | CS156 | 0.000 | 0.051 | 0.008 | 0.004 | 0.017 | 0.015 | 0.058 | 0.021 | 0.000 | 0.000 | 0.000 | 0.000 | 0.016 | 0.000 | 0.000 | 0.000 | 0.007 | 0.002 | 0.000 | 0.000 | 0.002 | 0.006 | 0.002 | 0.006 | 0.003 | 0.000 | 0.000 | 0.000 |
|  |  | CS157 | 0.000 | 0.115 | 0.011 | 0.006 | 0.022 | 0.016 | 0.020 | 0.005 | 0.000 | 0.000 | 0.000 | 0.000 | 0.000 | 0.000 | 0.000 | 0.000 | 0.012 | 0.001 | 0.000 | 0.000 | 0.017 | 0.007 | 0.002 | 0.002 | 0.000 | 0.000 | 0.000 | 0.000 |
|  |  | CS178 | 0.000 | 0.042 | 0.012 | 0.006 | 0.007 | 0.015 | 0.092 | 0.029 | 0.000 | 0.000 | 0.000 | 0.000 | 0.047 | 0.000 | 0.000 | 0.000 | 0.011 | 0.001 | 0.000 | 0.000 | 0.005 | 0.003 | 0.002 | 0.001 | 0.010 | 0.000 | 0.000 | 0.000 |
|  |  | CS179 | 0.000 | 0.144 | 0.013 | 0.010 | 0.017 | 0.017 | 0.091 | 0.031 | 0.000 | 0.000 | 0.000 | 0.000 | 0.046 | 0.000 | 0.000 | 0.000 | 0.000 | 0.000 | 0.000 | 0.000 | 0.008 | 0.004 | 0.001 | 0.012 | 0.005 | 0.000 | 0.000 | 0.000 |
|  |  | CS180 | 0.000 | 0.059 | 0.009 | 0.007 | 0.015 | 0.008 | 0.135 | 0.033 | 0.000 | 0.000 | 0.000 | 0.000 | 0.000 | 0.000 | 0.000 | 0.000 | 0.000 | 0.001 | 0.000 | 0.000 | 0.002 | 0.006 | 0.001 | 0.007 | 0.010 | 0.000 | 0.000 | 0.000 |
|  |  | CS193 | 0.000 | 0.031 | 0.009 | 0.005 | 0.012 | 0.012 | 0.101 | 0.021 | 0.000 | 0.000 | 0.000 | 0.000 | 0.099 | 0.000 | 0.000 | 0.000 | 0.000 | 0.001 | 0.000 | 0.000 | 0.001 | 0.001 | 0.003 | 0.000 | 0.002 | 0.000 | 0.000 | 0.000 |
|  |  | CS194 | 0.000 | 0.037 | 0.012 | 0.005 | 0.003 | 0.025 | 0.149 | 0.045 | 0.000 | 0.000 | 0.000 | 0.000 | 0.082 | 0.000 | 0.000 | 0.000 | 0.000 | 0.001 | 0.000 | 0.000 | 0.001 | 0.002 | 0.001 | 0.003 | 0.004 | 0.000 | 0.000 | 0.000 |
|  |  | CS195 | 0.000 | 0.051 | 0.005 | 0.006 | 0.006 | 0.010 | 0.113 | 0.028 | 0.000 | 0.000 | 0.000 | 0.000 | 0.061 | 0.000 | 0.000 | 0.000 | 0.000 | 0.001 | 0.000 | 0.000 | 0.007 | 0.001 | 0.001 | 0.000 | 0.002 | 0.000 | 0.000 | 0.000 |
|  |  | CS196 | 0.000 | 0.049 | 0.009 | 0.002 | 0.003 | 0.025 | 0.181 | 0.042 | 0.000 | 0.000 | 0.000 | 0.000 | 0.086 | 0.000 | 0.000 | 0.000 | 0.000 | 0.002 | 0.000 | 0.000 | 0.000 | 0.008 | 0.002 | 0.007 | 0.008 | 0.000 | 0.000 | 0.000 |
|  |  | CS197 | 0.000 | 0.037 | 0.010 | 0.005 | 0.013 | 0.020 | 0.109 | 0.037 | 0.000 | 0.000 | 0.000 | 0.000 | 0.080 | 0.000 | 0.000 | 0.000 | 0.000 | 0.001 | 0.000 | 0.000 | 0.001 | 0.001 | 0.004 | 0.000 | 0.002 | 0.000 | 0.000 | 0.000 |
|  |  | CS198 | 0.000 | 0.046 | 0.005 | 0.006 | 0.007 | 0.014 | 0.050 | 0.020 | 0.000 | 0.000 | 0.000 | 0.000 | 0.091 | 0.000 | 0.000 | 0.000 | 0.000 | 0.001 | 0.000 | 0.000 | 0.013 | 0.001 | 0.001 | 0.002 | 0.002 | 0.000 | 0.000 | 0.000 |
|  |  | CS199 | 0.000 | 0.080 | 0.010 | 0.003 | 0.014 | 0.031 | 0.086 | 0.026 | 0.000 | 0.000 | 0.000 | 0.000 | 0.063 | 0.000 | 0.000 | 0.000 | 0.000 | 0.001 | 0.000 | 0.000 | 0.000 | 0.002 | 0.004 | 0.000 | 0.005 | 0.000 | 0.000 | 0.000 |
|  |  | CS200 | 0.000 | 0.032 | 0.010 | 0.003 | 0.012 | 0.018 | 0.068 | 0.019 | 0.000 | 0.000 | 0.000 | 0.000 | 0.102 | 0.000 | 0.000 | 0.000 | 0.000 | 0.001 | 0.000 | 0.000 | 0.000 | 0.001 | 0.002 | 0.001 | 0.003 | 0.000 | 0.000 | 0.000 |
|  |  | CS201 | 0.000 | 0.052 | 0.013 | 0.006 | 0.011 | 0.040 | 0.101 | 0.027 | 0.000 | 0.000 | 0.000 | 0.000 | 0.092 | 0.000 | 0.000 | 0.000 | 0.000 | 0.001 | 0.000 | 0.000 | 0.000 | 0.002 | 0.010 | 0.000 | 0.008 | 0.000 | 0.000 | 0.000 |
|  |  | CS202 | 0.000 | 0.067 | 0.009 | 0.004 | 0.006 | 0.020 | 0.082 | 0.026 | 0.000 | 0.000 | 0.000 | 0.000 | 0.108 | 0.000 | 0.000 | 0.000 | 0.000 | 0.000 | 0.000 | 0.000 | 0.001 | 0.002 | 0.005 | 0.004 | 0.004 | 0.000 | 0.000 | 0.000 |
| **Wild type** | **BALB/c** | CS162 | 0.036 | 0.068 | 0.001 | 0.000 | 0.012 | 0.008 | 0.009 | 0.002 | 0.007 | 0.003 | 0.002 | 0.010 | 0.114 | 0.006 | 0.004 | 0.007 | 0.000 | 0.001 | 0.011 | 0.002 | 0.000 | 0.004 | 0.000 | 0.000 | 0.001 | 0.001 | 0.000 | 0.006 |
|  |  | CS163 | 0.021 | 0.056 | 0.001 | 0.000 | 0.012 | 0.001 | 0.030 | 0.018 | 0.016 | 0.008 | 0.002 | 0.007 | 0.099 | 0.010 | 0.001 | 0.002 | 0.000 | 0.003 | 0.013 | 0.000 | 0.004 | 0.002 | 0.000 | 0.000 | 0.000 | 0.001 | 0.000 | 0.001 |
|  |  | CS164 | 0.015 | 0.081 | 0.000 | 0.000 | 0.014 | 0.005 | 0.029 | 0.012 | 0.006 | 0.003 | 0.003 | 0.007 | 0.125 | 0.005 | 0.004 | 0.005 | 0.000 | 0.006 | 0.003 | 0.005 | 0.003 | 0.001 | 0.000 | 0.000 | 0.001 | 0.001 | 0.000 | 0.037 |
|  |  | CS165 | 0.022 | 0.033 | 0.000 | 0.000 | 0.019 | 0.010 | 0.008 | 0.006 | 0.025 | 0.002 | 0.002 | 0.012 | 0.082 | 0.009 | 0.000 | 0.002 | 0.000 | 0.001 | 0.016 | 0.000 | 0.001 | 0.000 | 0.001 | 0.000 | 0.000 | 0.002 | 0.001 | 0.004 |
|  |  | CS166 | 0.013 | 0.049 | 0.001 | 0.000 | 0.009 | 0.002 | 0.012 | 0.017 | 0.021 | 0.001 | 0.001 | 0.006 | 0.074 | 0.003 | 0.000 | 0.002 | 0.000 | 0.001 | 0.017 | 0.006 | 0.015 | 0.000 | 0.002 | 0.000 | 0.001 | 0.001 | 0.001 | 0.009 |
|  |  | CS167 | 0.019 | 0.030 | 0.000 | 0.000 | 0.013 | 0.011 | 0.032 | 0.017 | 0.011 | 0.003 | 0.002 | 0.006 | 0.074 | 0.002 | 0.003 | 0.005 | 0.000 | 0.002 | 0.025 | 0.011 | 0.003 | 0.001 | 0.003 | 0.000 | 0.001 | 0.001 | 0.001 | 0.019 |
|  |  | CS168 | 0.035 | 0.049 | 0.004 | 0.000 | 0.017 | 0.012 | 0.005 | 0.003 | 0.027 | 0.004 | 0.002 | 0.007 | 0.100 | 0.008 | 0.005 | 0.002 | 0.000 | 0.001 | 0.033 | 0.003 | 0.004 | 0.001 | 0.003 | 0.000 | 0.000 | 0.001 | 0.001 | 0.012 |
|  |  | CS169 | 0.029 | 0.073 | 0.021 | 0.000 | 0.024 | 0.006 | 0.045 | 0.030 | 0.013 | 0.002 | 0.007 | 0.007 | 0.000 | 0.005 | 0.007 | 0.003 | 0.000 | 0.004 | 0.002 | 0.000 | 0.001 | 0.005 | 0.002 | 0.000 | 0.002 | 0.002 | 0.000 | 0.007 |
|  |  | CS170 | 0.064 | 0.034 | 0.000 | 0.000 | 0.001 | 0.002 | 0.045 | 0.037 | 0.008 | 0.001 | 0.002 | 0.006 | 0.053 | 0.009 | 0.017 | 0.009 | 0.000 | 0.005 | 0.039 | 0.000 | 0.016 | 0.003 | 0.015 | 0.000 | 0.003 | 0.001 | 0.000 | 0.001 |
|  |  | CS171 | 0.045 | 0.041 | 0.000 | 0.000 | 0.002 | 0.000 | 0.007 | 0.002 | 0.004 | 0.007 | 0.005 | 0.004 | 0.095 | 0.010 | 0.007 | 0.051 | 0.000 | 0.004 | 0.048 | 0.000 | 0.006 | 0.004 | 0.002 | 0.000 | 0.003 | 0.001 | 0.001 | 0.030 |
|  |  | CS172 | 0.040 | 0.032 | 0.000 | 0.000 | 0.007 | 0.002 | 0.000 | 0.000 | 0.008 | 0.006 | 0.003 | 0.004 | 0.096 | 0.005 | 0.005 | 0.006 | 0.000 | 0.003 | 0.057 | 0.000 | 0.000 | 0.000 | 0.001 | 0.000 | 0.002 | 0.001 | 0.000 | 0.013 |
|  |  | CS173 | 0.021 | 0.038 | 0.000 | 0.000 | 0.001 | 0.000 | 0.000 | 0.000 | 0.010 | 0.002 | 0.003 | 0.007 | 0.128 | 0.003 | 0.002 | 0.027 | 0.000 | 0.003 | 0.011 | 0.000 | 0.016 | 0.011 | 0.002 | 0.000 | 0.001 | 0.001 | 0.000 | 0.004 |
|  |  | CS174 | 0.039 | 0.028 | 0.000 | 0.000 | 0.005 | 0.021 | 0.016 | 0.007 | 0.033 | 0.007 | 0.003 | 0.005 | 0.071 | 0.044 | 0.008 | 0.010 | 0.000 | 0.002 | 0.012 | 0.000 | 0.000 | 0.001 | 0.010 | 0.000 | 0.001 | 0.001 | 0.002 | 0.016 |
|  |  | CS175 | 0.044 | 0.035 | 0.000 | 0.000 | 0.014 | 0.019 | 0.000 | 0.001 | 0.030 | 0.004 | 0.003 | 0.004 | 0.095 | 0.002 | 0.010 | 0.012 | 0.000 | 0.001 | 0.001 | 0.000 | 0.000 | 0.000 | 0.002 | 0.000 | 0.001 | 0.001 | 0.001 | 0.019 |
|  |  | CS176 | 0.031 | 0.040 | 0.000 | 0.000 | 0.003 | 0.024 | 0.042 | 0.048 | 0.015 | 0.003 | 0.004 | 0.009 | 0.118 | 0.019 | 0.003 | 0.009 | 0.000 | 0.003 | 0.001 | 0.000 | 0.000 | 0.001 | 0.018 | 0.000 | 0.003 | 0.001 | 0.001 | 0.000 |
|  |  | CS177 | 0.017 | 0.027 | 0.000 | 0.000 | 0.007 | 0.005 | 0.066 | 0.045 | 0.007 | 0.024 | 0.005 | 0.005 | 0.088 | 0.008 | 0.005 | 0.005 | 0.000 | 0.004 | 0.003 | 0.000 | 0.007 | 0.002 | 0.000 | 0.000 | 0.002 | 0.000 | 0.001 | 0.002 |
|  | **C57BL/6** | CS203 | 0.007 | 0.000 | 0.010 | 0.000 | 0.001 | 0.017 | 0.030 | 0.037 | 0.009 | 0.033 | 0.001 | 0.003 | 0.007 | 0.055 | 0.009 | 0.003 | 0.126 | 0.000 | 0.005 | 0.000 | 0.000 | 0.003 | 0.000 | 0.000 | 0.014 | 0.001 | 0.008 | 0.082 |
|  |  | CS204 | 0.009 | 0.003 | 0.013 | 0.000 | 0.003 | 0.028 | 0.044 | 0.014 | 0.011 | 0.043 | 0.001 | 0.005 | 0.013 | 0.051 | 0.006 | 0.001 | 0.112 | 0.001 | 0.005 | 0.000 | 0.000 | 0.001 | 0.000 | 0.000 | 0.003 | 0.000 | 0.005 | 0.037 |
|  |  | CS205 | 0.008 | 0.013 | 0.008 | 0.000 | 0.005 | 0.003 | 0.140 | 0.068 | 0.005 | 0.014 | 0.003 | 0.006 | 0.063 | 0.015 | 0.000 | 0.006 | 0.000 | 0.008 | 0.019 | 0.000 | 0.000 | 0.000 | 0.001 | 0.000 | 0.005 | 0.001 | 0.001 | 0.015 |
|  |  | CS206 | 0.012 | 0.015 | 0.013 | 0.000 | 0.005 | 0.009 | 0.107 | 0.039 | 0.010 | 0.044 | 0.003 | 0.005 | 0.073 | 0.016 | 0.002 | 0.003 | 0.015 | 0.004 | 0.011 | 0.000 | 0.000 | 0.001 | 0.001 | 0.000 | 0.005 | 0.001 | 0.001 | 0.005 |
|  |  | CS207 | 0.002 | 0.003 | 0.001 | 0.000 | 0.002 | 0.010 | 0.129 | 0.071 | 0.001 | 0.006 | 0.002 | 0.001 | 0.047 | 0.011 | 0.001 | 0.027 | 0.011 | 0.002 | 0.016 | 0.000 | 0.003 | 0.000 | 0.003 | 0.000 | 0.003 | 0.000 | 0.001 | 0.046 |
|  |  | CS208 | 0.011 | 0.007 | 0.017 | 0.000 | 0.021 | 0.010 | 0.092 | 0.030 | 0.014 | 0.008 | 0.006 | 0.008 | 0.066 | 0.023 | 0.001 | 0.004 | 0.001 | 0.005 | 0.018 | 0.000 | 0.000 | 0.000 | 0.013 | 0.000 | 0.007 | 0.001 | 0.002 | 0.036 |
|  |  | CS209 | 0.003 | 0.002 | 0.005 | 0.000 | 0.004 | 0.015 | 0.012 | 0.009 | 0.001 | 0.004 | 0.002 | 0.005 | 0.050 | 0.030 | 0.010 | 0.030 | 0.040 | 0.002 | 0.034 | 0.000 | 0.000 | 0.000 | 0.027 | 0.000 | 0.005 | 0.000 | 0.002 | 0.050 |
|  |  | CS210 | 0.004 | 0.005 | 0.012 | 0.000 | 0.023 | 0.019 | 0.001 | 0.000 | 0.008 | 0.004 | 0.004 | 0.007 | 0.089 | 0.018 | 0.001 | 0.020 | 0.000 | 0.001 | 0.026 | 0.000 | 0.000 | 0.000 | 0.006 | 0.000 | 0.006 | 0.001 | 0.003 | 0.010 |
|  |  | CS211 | 0.003 | 0.009 | 0.004 | 0.000 | 0.006 | 0.014 | 0.083 | 0.058 | 0.001 | 0.015 | 0.002 | 0.005 | 0.034 | 0.029 | 0.005 | 0.013 | 0.028 | 0.002 | 0.026 | 0.000 | 0.000 | 0.000 | 0.015 | 0.000 | 0.008 | 0.000 | 0.002 | 0.052 |
|  |  | CS212 | 0.002 | 0.008 | 0.009 | 0.000 | 0.004 | 0.006 | 0.158 | 0.051 | 0.005 | 0.020 | 0.006 | 0.002 | 0.032 | 0.007 | 0.000 | 0.005 | 0.006 | 0.006 | 0.012 | 0.000 | 0.000 | 0.000 | 0.014 | 0.000 | 0.008 | 0.000 | 0.001 | 0.045 |
|  |  | CS213 | 0.000 | 0.002 | 0.007 | 0.000 | 0.014 | 0.033 | 0.056 | 0.045 | 0.004 | 0.013 | 0.003 | 0.000 | 0.094 | 0.029 | 0.002 | 0.007 | 0.013 | 0.004 | 0.023 | 0.000 | 0.000 | 0.001 | 0.009 | 0.000 | 0.010 | 0.000 | 0.001 | 0.084 |
|  |  | CS214 | 0.003 | 0.014 | 0.016 | 0.000 | 0.006 | 0.028 | 0.004 | 0.001 | 0.009 | 0.014 | 0.001 | 0.005 | 0.070 | 0.027 | 0.000 | 0.003 | 0.017 | 0.002 | 0.019 | 0.000 | 0.000 | 0.001 | 0.003 | 0.000 | 0.002 | 0.001 | 0.002 | 0.018 |
|  |  | CS215 | 0.006 | 0.002 | 0.010 | 0.000 | 0.006 | 0.013 | 0.072 | 0.033 | 0.008 | 0.025 | 0.003 | 0.000 | 0.047 | 0.019 | 0.003 | 0.006 | 0.001 | 0.005 | 0.023 | 0.000 | 0.000 | 0.001 | 0.002 | 0.000 | 0.008 | 0.001 | 0.002 | 0.021 |
|  |  | CS216 | 0.006 | 0.049 | 0.011 | 0.000 | 0.007 | 0.015 | 0.006 | 0.006 | 0.032 | 0.016 | 0.002 | 0.007 | 0.044 | 0.016 | 0.001 | 0.003 | 0.008 | 0.001 | 0.027 | 0.000 | 0.000 | 0.002 | 0.000 | 0.000 | 0.004 | 0.002 | 0.001 | 0.006 |
|  |  | CS217 | 0.015 | 0.001 | 0.003 | 0.000 | 0.008 | 0.029 | 0.067 | 0.026 | 0.015 | 0.009 | 0.003 | 0.000 | 0.067 | 0.023 | 0.003 | 0.007 | 0.002 | 0.002 | 0.019 | 0.000 | 0.008 | 0.000 | 0.006 | 0.000 | 0.007 | 0.001 | 0.001 | 0.032 |
|  |  | CS218 | 0.002 | 0.010 | 0.007 | 0.000 | 0.011 | 0.018 | 0.001 | 0.001 | 0.008 | 0.021 | 0.002 | 0.003 | 0.053 | 0.015 | 0.002 | 0.005 | 0.014 | 0.002 | 0.027 | 0.000 | 0.005 | 0.001 | 0.005 | 0.000 | 0.003 | 0.001 | 0.002 | 0.003 |
|  |  | CS219 | 0.019 | 0.007 | 0.009 | 0.000 | 0.006 | 0.028 | 0.051 | 0.026 | 0.010 | 0.004 | 0.003 | 0.000 | 0.110 | 0.013 | 0.004 | 0.003 | 0.000 | 0.003 | 0.019 | 0.000 | 0.000 | 0.002 | 0.001 | 0.000 | 0.003 | 0.002 | 0.000 | 0.023 |
|  |  | CS220 | 0.004 | 0.007 | 0.009 | 0.000 | 0.015 | 0.009 | 0.000 | 0.003 | 0.030 | 0.015 | 0.002 | 0.003 | 0.086 | 0.013 | 0.002 | 0.008 | 0.002 | 0.001 | 0.023 | 0.000 | 0.000 | 0.003 | 0.000 | 0.000 | 0.002 | 0.001 | 0.002 | 0.004 |

**Supplementary Table 5.** Differentiation in microbial composition between BALB/c and C57BL/6 wild-type mice. Distinctions in microbiota composition were assessed using LEfSe where OTUs with an LDA > 3.0 are reported.

| Group | OTU | LDA | p-value | Genus |
| --- | --- | --- | --- | --- |
| BALB/c | Otu100 | 3.02 | 2.78E-02 | Uncultured Bacteria |
|  | Otu49 | 3.96 | 1.35E-06 | *Bacteroides* |
|  | Otu92 | 3.54 | 2.53E-04 | *Odoribacter* |
|  | Otu50 | 3.75 | 3.96E-03 | Uncultured *Porphyromonadaceae* |
|  | Otu14 | 4.00 | 1.50E-03 | *Alistipes* |
|  | Otu11 | 4.22 | 4.45E-06 | *Alistipes* |
|  | Otu229 | 3.19 | 3.38E-02 | *Alistipes* |
|  | Otu35 | 3.19 | 3.19E-04 | *Clostridium* *XlVa* |
|  | Otu61 | 3.48 | 3.24E-02 | *Clostridium* *XlVb* |
|  | Otu96 | 3.10 | 1.43E-02 | *Dorea* |
|  | Otu19 | 3.37 | 3.85E-02 | Uncultured *Lachnospiraceae* |
|  | Otu40 | 3.22 | 1.22E-02 | Uncultured *Lachnospiraceae* |
|  | Otu57 | 3.25 | 1.74E-02 | Uncultured *Lachnospiraceae* |
|  | Otu59 | 3.13 | 1.33E-03 | Uncultured *Lachnospiraceae* |
|  | Otu705 | 3.38 | 1.15E-04 | Uncultured *Lachnospiraceae* |
|  | Otu1605 | 3.15 | 3.72E-03 | Uncultured *Lachnospiraceae* |
|  | Otu128 | 3.22 | 1.27E-05 | Uncultured *Lachnospiraceae* |
|  | Otu106 | 3.15 | 5.10E-04 | *Ruminococcus2* |
|  | Otu62 | 3.82 | 3.45E-05 | *Butyricicoccus* |
|  | Otu70 | 3.05 | 1.11E-02 | *Oscillibacter* |
|  | Otu324 | 3.21 | 6.14E-06 | *Oscillibacter* |
|  | Otu155 | 3.12 | 3.00E-03 | *Oscillibacter* |
|  | Otu109 | 3.05 | 4.95E-05 | Uncultured *Ruminococcaceae* |
|  | Otu117 | 3.08 | 4.11E-05 | Uncultured *Ruminococcaceae* |
|  | Otu147 | 3.13 | 6.76E-04 | Uncultured *Ruminococcaceae* |
|  | Otu183 | 3.32 | 1.33E-03 | Uncultured *Ruminococcaceae* |
|  | Otu184 | 3.12 | 3.35E-04 | Uncultured *Ruminococcaceae* |
| C57BL/6 | Otu216 | 3.01 | 8.90E-05 | *Enterorhabdus* |
|  | Otu81 | 3.57 | 2.55E-04 | *Bacteroides* |
|  | Otu165 | 3.07 | 5.47E-04 | *Parabacteroides* |
|  | Otu37 | 3.77 | 2.52E-03 | Uncultured *Porphyromonadaceae* |
|  | Otu56 | 3.86 | 1.94E-04 | Uncultured *Porphyromonadaceae* |
|  | Otu68 | 3.54 | 5.24E-04 | Uncultured *Porphyromonadaceae* |
|  | Otu136 | 3.06 | 4.34E-05 | Uncultured *Porphyromonadaceae* |
|  | Otu211 | 3.23 | 3.75E-03 | Uncultured *Porphyromonadaceae* |
|  | Otu21 | 3.97 | 7.89E-03 | *Alloprevotella* |
|  | Otu67 | 3.61 | 1.69E-04 | *Alistipes* |
|  | Otu337 | 3.38 | 2.18E-05 | *Alistipes* |
|  | Otu86 | 3.44 | 1.18E-02 | *Alistipes* |
|  | Otu10 | 4.15 | 4.92E-02 | *Lactobacillus* |
|  | Otu33 | 3.45 | 2.03E-02 | *Clostridium XlVa* |
|  | Otu88 | 3.25 | 2.08E-02 | Uncultured *Lachnospiraceae* |
|  | Otu27 | 4.30 | 1.49E-05 | Uncultured *Erysipelotrichaceae* |
|  | Otu98 | 3.31 | 3.79E-05 | Uncultured *Erysipelotrichaceae* |
|  | Otu134 | 3.48 | 8.51E-06 | *Parasutterella* |
|  | Otu6 | 4.37 | 5.01E-07 | *Akkermansia* |

**Supplementary Table 6.** BALB/c and C57BL/6 wild-type animals harbor distinct metabolic activity. Distinctions in metabolic activity were assessed by conducting functional analysis with PICRUSt using the EC database, followed by non-parametric Kruskal-Wallis testing with Welch’s uncorrected post-hoc test and Benjamini-Hochberg FDR correction. Only results with an effect size > 0.5 are reported.

| Group | description | p-values (corrected) | Effect size | BALBc: mean rel. freq. (%) | BALBc: std. dev. (%) | C57BL: mean rel. freq. (%) | C57BL: std. dev. (%) |
| --- | --- | --- | --- | --- | --- | --- | --- |
| C57BL/6 | superpathway of menaquinol-9 biosynthesis | 0.0002 | 0.57 | 0.010 | 0.005 | 0.057 | 0.027 |
| C57BL/6 | superpathway of menaquinol-6 biosynthesis I | 0.0002 | 0.57 | 0.010 | 0.005 | 0.057 | 0.027 |
| C57BL/6 | superpathway of demethylmenaquinol-6 biosynthesis I | 0.0002 | 0.56 | 0.007 | 0.003 | 0.040 | 0.020 |
| C57BL/6 | superpathway of demethylmenaquinol-9 biosynthesis | 0.0001 | 0.56 | 0.007 | 0.003 | 0.040 | 0.020 |
| C57BL/6 | superpathway of menaquinol-10 biosynthesis | 0.0002 | 0.57 | 0.010 | 0.005 | 0.057 | 0.027 |

**Supplementary Table 7.** Association of CCGs with differentially abundant OTUs between BALB/c and C57BL/6 mice. The relative abundances of differentially abundant OTUs identified by linear discriminant analysis effect size (LEfSe, LDA > 3.0) were associated to the analyzed CCGs. OTU: Operational taxonomical unit; LDA: linear discriminant analysis score; SD: standard deviation; CCG: cytokine, chemokine and growth factors.

| **Otu** | **Classification** | **CCG** | **Pearson coefficient** | **FDR-corrected**  ***P*-value** |
| --- | --- | --- | --- | --- |
| Otu165 | *Parabacteroides* | GM_CSF | 0.675 | 5.32E-05 |
| Otu67 | *Alistipes* | IL_2 | 0.733 | 1.06E-04 |
| Otu81 | *Bacteroides* | MCP_1 | 0.679 | 1.60E-04 |
| Otu136 | Uncultured *Porphyromonadaceae* | MCP_1 | 0.733 | 2.13E-04 |
| Otu11 | *Alistipes* | GM_CSF | -0.681 | 2.66E-04 |
| Otu11 | *Alistipes* | IL_10 | -0.713 | 3.19E-04 |
| Otu11 | *Alistipes* | IL_1B | -0.675 | 3.72E-04 |
| Otu11 | *Alistipes* | IL_2 | -0.808 | 4.26E-04 |
| Otu67 | *Alistipes* | IL_10 | 0.659 | 4.79E-04 |
| Otu165 | *Parabacteroides* | IL_1B | 0.655 | 5.32E-04 |
| Otu92 | *Odoribacter* | MIP_2 | -0.672 | 5.85E-04 |
| Otu165 | *Parabacteroides* | IL_2 | 0.648 | 6.38E-04 |
| Otu67 | *Alistipes* | MCP_1 | 0.639 | 6.91E-04 |
| Otu92 | *Odoribacter* | IL_4 | -0.629 | 7.45E-04 |
| Otu56 | Uncultured *Porphyromonadaceae* | MIP_2 | 0.623 | 7.98E-04 |
| Otu155 | *Oscillibacter* | GM_CSF | -0.622 | 8.51E-04 |
| Otu67 | *Alistipes* | GM_CSF | 0.618 | 9.04E-04 |
| Otu6 | *Akkermansia* | IL_2 | 0.619 | 9.57E-04 |
| Otu211 | Uncultured *Porphyromonadaceae* | IL_2 | 0.619 | 1.01E-03 |
| Otu81 | *Bacteroides* | IL_10 | 0.618 | 1.06E-03 |
| Otu68 | Uncultured *Porphyromonadaceae* | IL_2 | 0.617 | 1.12E-03 |
| Otu136 | Uncultured *Porphyromonadaceae* | IL_2 | 0.614 | 1.17E-03 |
| Otu61 | *Clostridium XlVb* | IL_6 | 0.611 | 1.22E-03 |
| Otu49 | *Bacteroides* | MIP_2 | -0.599 | 1.28E-03 |
| Otu165 | *Parabacteroides* | IL_10 | 0.595 | 1.33E-03 |
| Otu67 | *Alistipes* | IL_1B | 0.593 | 1.38E-03 |
| Otu21 | *Alloprevotella* | IL_2 | 0.593 | 1.44E-03 |
| Otu155 | *Oscillibacter* | IL_2 | -0.593 | 1.49E-03 |
| Otu136 | Uncultured *Porphyromonadaceae* | IL_10 | 0.582 | 1.54E-03 |
| Otu67 | *Alistipes* | IL_4 | -0.580 | 1.60E-03 |
| Otu136 | Uncultured *Porphyromonadaceae* | IL_1B | 0.578 | 1.65E-03 |
| Otu68 | Uncultured *Porphyromonadaceae* | IL_22 | 0.576 | 1.70E-03 |
| Otu27 | Uncultured *Erysipelotrichaceae* | IL_2 | 0.573 | 1.76E-03 |
| Otu117 | Uncultured *Ruminococcaceae* | IL_2 | -0.569 | 1.81E-03 |
| Otu11 | *Alistipes* | MCP_1 | -0.562 | 1.86E-03 |
| Otu67 | *Alistipes* | IL_17A | 0.561 | 1.91E-03 |
| Otu98 | Uncultured *Erysipelotrichaceae* | IL_2 | 0.556 | 1.97E-03 |
| Otu165 | *Parabacteroides* | MCP_1 | 0.554 | 2.02E-03 |
| Otu324 | *Oscillibacter* | IL_2 | -0.553 | 2.07E-03 |
| Otu216 | *Enterorhabdus* | IL_22 | 0.550 | 2.13E-03 |
| Otu134 | *Parasutterella* | MIP_2 | 0.547 | 2.18E-03 |
| Otu705 | Uncultured *Lachnospiraceae* | IFN_g | 0.545 | 2.23E-03 |
| Otu86 | *Alistipes* | MIP_2 | 0.543 | 2.29E-03 |
| Otu128 | Uncultured *Lachnospiraceae* | IL_12p70 | 0.536 | 2.34E-03 |
| Otu211 | Uncultured *Porphyromonadaceae* | IL_10 | 0.534 | 2.39E-03 |
| Otu165 | *Parabacteroides* | IL_4 | -0.532 | 2.45E-03 |
| Otu183 | Uncultured *Ruminococcaceae* | IL_17F | 0.528 | 2.50E-03 |
| Otu86 | *Alistipes* | KC_GRO | 0.528 | 2.55E-03 |
| Otu117 | Uncultured *Ruminococcaceae* | IL_1B | -0.526 | 2.61E-03 |
| Otu35 | *Clostridium XlVa* | IFN_g | 0.524 | 2.66E-03 |
| Otu33 | *Clostridium XlVa* | IL_17A | 0.524 | 2.71E-03 |
| Otu96 | *Dorea* | IL_12p70 | 0.521 | 2.77E-03 |
| Otu211 | Uncultured *Porphyromonadaceae* | IL_22 | 0.520 | 2.82E-03 |
| Otu117 | Uncultured *Ruminococcaceae* | IL_10 | -0.515 | 2.87E-03 |
| Otu6 | *Akkermansia* | GM_CSF | 0.513 | 2.93E-03 |
| Otu50 | Uncultured *Porphyromonadaceae* | MIP_2 | -0.513 | 2.98E-03 |
| Otu86 | *Alistipes* | IL_23 | 0.509 | 3.03E-03 |
| Otu6 | *Akkermansia* | MCP_1 | 0.507 | 3.09E-03 |
| Otu81 | *Bacteroides* | IL_2 | 0.505 | 3.14E-03 |
| Otu59 | Uncultured *Lachnospiraceae* | MIP_2 | -0.503 | 3.19E-03 |
| Otu81 | *Bacteroides* | IL_1B | 0.503 | 3.24E-03 |
| Otu147 | Uncultured *Ruminococcaceae* | IL_2 | -0.501 | 3.30E-03 |

**Supplementary Table 8.** Differentiation in microbial composition between BALB/c genotypes. Distinctions in microbiota composition were assessed using LEfSe where OTUs with an LDA > 3.0 are reported.

| Group | OTU | LDA | p-value | Genus |
| --- | --- | --- | --- | --- |
| IL-33^-/-^ | Otu80 | 3.24 | 1.36E-02 | *Acetatifactor* |
|  | Otu156 | 3.26 | 8.52E-06 | *Acetatifactor* |
|  | Otu11 | 4.00 | 8.80E-03 | *Alistipes* |
|  | Otu130 | 3.34 | 9.22E-06 | Uncultured Bacteria |
|  | Otu49 | 3.96 | 1.12E-05 | *Bacteroides* |
|  | Otu125 | 3.01 | 2.71E-02 | Uncultured Clostridiales |
|  | Otu420 | 3.17 | 1.56E-03 | Uncultured Clostridiales |
|  | Otu146 | 3.34 | 5.09E-03 | Uncultured Clostridiales |
|  | Otu456 | 3.52 | 5.34E-03 | *Clostridium* *XlVa* |
|  | Otu24 | 3.85 | 3.28E-07 | *Desulfovibrio* |
|  | Otu312 | 4.50 | 1.80E-02 | Uncultured *Lachnospiraceae* |
|  | Otu15 | 4.26 | 1.28E-06 | Uncultured *Lachnospiraceae* |
|  | Otu17 | 4.36 | 5.23E-06 | Uncultured *Lachnospiraceae* |
|  | Otu32 | 3.64 | 2.68E-03 | Uncultured *Lachnospiraceae* |
|  | Otu44 | 3.80 | 4.47E-05 | Uncultured *Lachnospiraceae* |
|  | Otu51 | 3.66 | 2.68E-03 | Uncultured *Lachnospiraceae* |
|  | Otu52 | 3.73 | 1.78E-05 | Uncultured *Lachnospiraceae* |
|  | Otu59 | 3.29 | 2.20E-03 | Uncultured *Lachnospiraceae* |
|  | Otu88 | 3.56 | 1.37E-05 | Uncultured *Lachnospiraceae* |
|  | Otu242 | 3.03 | 8.42E-04 | Uncultured *Lachnospiraceae* |
|  | Otu110 | 3.21 | 1.24E-06 | Uncultured *Lachnospiraceae* |
|  | Otu516 | 3.21 | 1.34E-02 | Uncultured *Lachnospiraceae* |
|  | Otu127 | 3.31 | 1.30E-05 | Uncultured *Lachnospiraceae* |
|  | Otu131 | 3.36 | 1.04E-05 | Uncultured *Lachnospiraceae* |
|  | Otu143 | 3.05 | 4.90E-05 | Uncultured *Lachnospiraceae* |
|  | Otu186 | 3.02 | 1.44E-03 | Uncultured *Lachnospiraceae* |
|  | Otu213 | 3.25 | 4.42E-05 | Uncultured *Lachnospiraceae* |
|  | Otu70 | 3.62 | 1.07E-04 | *Oscillibacter* |
|  | Otu155 | 3.28 | 7.13E-04 | *Oscillibacter* |
|  | Otu50 | 3.62 | 3.90E-02 | Uncultured *Porphyromonadaceae* |
|  | Otu56 | 3.59 | 1.11E-04 | Uncultured *Porphyromonadaceae* |
|  | Otu64 | 3.77 | 1.69E-04 | Uncultured *Porphyromonadaceae* |
|  | Otu87 | 3.78 | 5.74E-07 | Uncultured *Porphyromonadaceae* |
|  | Otu102 | 3.36 | 2.06E-04 | Uncultured *Porphyromonadaceae* |
|  | Otu113 | 3.03 | 7.91E-03 | Uncultured *Porphyromonadaceae* |
|  | Otu124 | 3.58 | 5.03E-04 | Uncultured *Porphyromonadaceae* |
|  | Otu132 | 3.14 | 4.25E-05 | Uncultured *Porphyromonadaceae* |
|  | Otu175 | 3.31 | 3.00E-04 | Uncultured Porphyromonadaceae |
|  | Otu29 | 3.81 | 1.17E-02 | *Prevotella* |
|  | Otu30 | 3.70 | 4.03E-06 | Uncultured *Ruminococcaceae* |
|  | Otu66 | 3.68 | 6.83E-05 | *Ruminococcus* |
| IL-4Rα^-/-^ | Otu337 | 3.81 | 8.13E-06 | *Alistipes* |
|  | Otu83 | 3.66 | 2.26E-07 | *Anaeroplasma* |
|  | Otu100 | 3.32 | 1.64E-02 | Uncultured Bacteria |
|  | Otu78 | 3.69 | 1.43E-04 | Uncultured Bacteroidales |
|  | Otu33 | 3.51 | 4.49E-05 | *Clostridium XlVa* |
|  | Otu35 | 3.65 | 2.84E-04 | *Clostridium XlVa* |
|  | Otu7 | 4.70 | 4.62E-07 | Uncultured *Lachnospiraceae* |
|  | Otu41 | 3.43 | 4.98E-04 | Uncultured *Lachnospiraceae* |
|  | Otu53 | 3.60 | 1.59E-05 | Uncultured *Lachnospiraceae* |
|  | Otu144 | 3.20 | 6.62E-03 | Uncultured *Lachnospiraceae* |
|  | Otu157 | 3.08 | 3.58E-04 | Uncultured *Lachnospiraceae* |
|  | Otu26 | 4.14 | 6.93E-07 | *Odoribacter* |
|  | Otu134 | 3.13 | 6.04E-07 | *Parasutterella* |
|  | Otu37 | 4.25 | 2.87E-06 | Uncultured *Porphyromonadaceae* |
|  | Otu47 | 3.90 | 3.28E-04 | Uncultured *Porphyromonadaceae* |
|  | Otu99 | 3.06 | 2.64E-09 | Uncultured *Porphyromonadaceae* |
|  | Otu115 | 3.01 | 1.11E-05 | Uncultured *Porphyromonadaceae* |
|  | Otu136 | 3.20 | 2.39E-03 | Uncultured *Porphyromonadaceae* |
|  | Otu145 | 3.30 | 2.17E-08 | Uncultured *Porphyromonadaceae* |
|  | Otu217 | 3.21 | 7.14E-09 | Uncultured *Porphyromonadaceae* |
|  | Otu187 | 3.05 | 1.14E-05 | Uncultured *Ruminococcaceae* |
|  | Otu106 | 3.27 | 1.40E-02 | *Ruminococcus2* |
|  | Otu844 | 3.03 | 8.73E-07 | *Ruminococcus2* |
| BALB/c | Otu14 | 4.53 | 1.90E-07 | *Alistipes* |
|  | Otu229 | 3.32 | 3.23E-05 | *Alistipes* |
|  | Otu21 | 3.66 | 1.10E-07 | *Alloprevotella* |
|  | Otu162 | 3.10 | 4.50E-04 | Uncultured Bacteroidales |
|  | Otu63 | 3.55 | 6.43E-08 | *Bacteroides* |
|  | Otu62 | 3.72 | 5.62E-04 | *Butyricicoccus* |
|  | Otu104 | 3.21 | 7.98E-06 | Uncultured Clostridiales |
|  | Otu18 | 4.01 | 1.02E-08 | Uncultured *Desulfovibrionaceae* |
|  | Otu96 | 3.28 | 2.85E-03 | *Dorea* |
|  | Otu5 | 4.19 | 1.84E-02 | Uncultured *Lachnospiraceae* |
|  | Otu13 | 3.96 | 5.44E-03 | Uncultured *Lachnospiraceae* |
|  | Otu19 | 3.63 | 2.54E-03 | Uncultured *Lachnospiraceae* |
|  | Otu1085 | 3.51 | 9.83E-06 | Uncultured *Lachnospiraceae* |
|  | Otu158 | 3.41 | 1.40E-06 | Uncultured *Lachnospiraceae* |
|  | Otu678 | 3.40 | 2.65E-06 | Uncultured *Lachnospiraceae* |
|  | Otu55 | 3.47 | 2.79E-02 | Uncultured *Lachnospiraceae* |
|  | Otu58 | 3.00 | 1.05E-05 | Uncultured *Lachnospiraceae* |
|  | Otu902 | 3.64 | 6.31E-07 | Uncultured *Lachnospiraceae* |
|  | Otu705 | 3.31 | 1.29E-04 | Uncultured *Lachnospiraceae* |
|  | Otu581 | 3.23 | 6.27E-07 | Uncultured *Lachnospiraceae* |
|  | Otu105 | 3.17 | 9.94E-04 | Uncultured *Lachnospiraceae* |
|  | Otu1605 | 3.28 | 5.53E-03 | Uncultured *Lachnospiraceae* |
|  | Otu128 | 3.22 | 3.72E-06 | Uncultured *Lachnospiraceae* |
|  | Otu329 | 3.15 | 7.92E-03 | Uncultured *Lachnospiraceae* |
|  | Otu8 | 3.47 | 1.80E-04 | *Lactobacillus* |
|  | Otu92 | 3.81 | 8.32E-07 | *Odoribacter* |
|  | Otu324 | 3.24 | 1.21E-05 | *Oscillibacter* |
|  | Otu60 | 3.47 | 3.57E-04 | Uncultured *Porphyromonadaceae* |
|  | Otu91 | 3.55 | 1.03E-06 | Uncultured *Porphyromonadaceae* |
|  | Otu107 | 3.44 | 1.47E-08 | Uncultured *Porphyromonadaceae* |
|  | Otu118 | 3.38 | 7.64E-04 | Uncultured *Porphyromonadaceae* |
|  | Otu73 | 3.23 | 1.06E-02 | Uncultured *Ruminococcaceae* |

**Supplementary Table 9.** BALB/c wild-type animals harbor distinct metabolic activity compared to IL-33^-/-^ and IL-4Rα^-/-^ knock-out animals constructed in BALB/c background. Distinctions in metabolic activity were assessed by conducting functional analysis with PICRUSt using the EC database, followed by non-parametric Kruskal-Wallis testing with Welch’s uncorrected post-hoc test and Benjamini-Hochberg FDR correction. Only results with an effect size > 0.5 are reported.

| Group | description | p-values (corrected) | Effect size | IL-33^-/-^: mean rel. freq. (%) | IL-33^-/-^: std. dev. (%) | IL-4Rα^-/-^: mean rel. freq. (%) | IL-4Rα^-/-^: std. dev. (%) | BALBc: mean rel. freq. (%) | BALBc: std. dev. (%) |
| --- | --- | --- | --- | --- | --- | --- | --- | --- | --- |
| BALB/c | superpathway of purine nucleotides de novo biosynthesis II | 1.21E-05 | 0.70 | 0.278 | 0.047 | 0.300 | 0.032 | 0.418 | 0.043 |
|  | superpathway of histidine, purine, and pyrimidine biosynthesis | 9.00E-06 | 0.81 | 0.214 | 0.032 | 0.269 | 0.036 | 0.379 | 0.035 |
|  | superpathway of sulfur oxidation (Acidianus ambivalens) | 1.15E-05 | 0.69 | 0.034 | 0.009 | 0.040 | 0.012 | 0.116 | 0.040 |
|  | superpathway of L-methionine biosynthesis (by sulfhydrylation) | 7.60E-06 | 0.85 | 0.044 | 0.014 | 0.113 | 0.018 | 0.156 | 0.025 |
|  | superpathway of guanosine nucleotides de novo biosynthesis II | 1.06E-05 | 0.68 | 0.191 | 0.037 | 0.206 | 0.025 | 0.302 | 0.039 |
|  | pyrimidine deoxyribonucleotides de novo biosynthesis III | 9.63E-06 | 0.64 | 0.117 | 0.022 | 0.132 | 0.016 | 0.185 | 0.027 |
|  | thiazole biosynthesis II (Bacillus) | 1.14E-05 | 0.71 | 0.042 | 0.010 | 0.045 | 0.011 | 0.111 | 0.031 |
|  | superpathway of thiamin diphosphate biosynthesis II | 1.03E-05 | 0.75 | 0.156 | 0.030 | 0.164 | 0.028 | 0.298 | 0.052 |
|  | pyrimidine deoxyribonucleotides de novo biosynthesis I | 1.11E-05 | 0.68 | 0.127 | 0.024 | 0.138 | 0.018 | 0.207 | 0.029 |
|  | pyrimidine deoxyribonucleotides de novo biosynthesis II | 1.35E-05 | 0.70 | 0.219 | 0.035 | 0.237 | 0.025 | 0.324 | 0.031 |
|  | superpathway of pyrimidine ribonucleosides salvage | 1.04E-05 | 0.69 | 0.187 | 0.037 | 0.203 | 0.026 | 0.307 | 0.042 |
|  | pyrimidine deoxyribonucleotide phosphorylation | 1.11E-05 | 0.70 | 0.114 | 0.023 | 0.125 | 0.017 | 0.193 | 0.028 |
|  | superpathway of pyrimidine deoxyribonucleoside salvage | 1.21E-05 | 0.71 | 0.163 | 0.029 | 0.178 | 0.021 | 0.258 | 0.031 |
|  | superpathway of pyrimidine deoxyribonucleotides de novo biosynthesis | 1.27E-05 | 0.71 | 0.173 | 0.031 | 0.191 | 0.023 | 0.270 | 0.029 |
|  | superpathway of guanosine nucleotides de novo biosynthesis I | 1.10E-05 | 0.68 | 0.168 | 0.033 | 0.181 | 0.023 | 0.269 | 0.037 |
|  | inosine-5'-phosphate biosynthesis III | 7.42E-06 | 0.76 | 0.100 | 0.019 | 0.175 | 0.050 | 0.301 | 0.064 |
|  | myo-, chiro- and scillo-inositol degradation | 1.11E-04 | 0.66 | 0.036 | 0.007 | 0.037 | 0.020 | 0.097 | 0.028 |
|  | mannan degradation | 1.27E-04 | 0.53 | 0.121 | 0.033 | 0.065 | 0.014 | 0.133 | 0.030 |
|  | superpathway of purine nucleotides de novo biosynthesis I | 9.77E-06 | 0.68 | 0.267 | 0.046 | 0.285 | 0.030 | 0.397 | 0.043 |
|  | tRNA processing | 1.45E-05 | 0.72 | 0.024 | 0.007 | 0.035 | 0.008 | 0.075 | 0.021 |
|  | superpathway of pyrimidine deoxyribonucleotides de novo biosynthesis (E. coli) | 1.17E-05 | 0.69 | 0.215 | 0.035 | 0.232 | 0.024 | 0.316 | 0.031 |
|  | sulfate reduction I (assimilatory) | 1.32E-05 | 0.86 | 0.017 | 0.006 | 0.055 | 0.011 | 0.087 | 0.016 |
|  | superpathway of sulfate assimilation and cysteine biosynthesis | 7.16E-06 | 0.86 | 0.038 | 0.013 | 0.114 | 0.020 | 0.172 | 0.029 |
| IL-33^-/-^ | N10-formyl-tetrahydrofolate biosynthesis | 1.39E-05 | 0.67 | 0.813 | 0.012 | 0.762 | 0.017 | 0.765 | 0.018 |
|  | superpathway of L-aspartate and L-asparagine biosynthesis | 1.41E-04 | 0.52 | 0.794 | 0.024 | 0.767 | 0.019 | 0.740 | 0.021 |
|  | Calvin-Benson-Bassham cycle | 1.08E-04 | 0.59 | 1.061 | 0.031 | 0.987 | 0.021 | 0.992 | 0.030 |
|  | L-lysine biosynthesis I | 4.53E-05 | 0.58 | 0.813 | 0.044 | 0.793 | 0.026 | 0.715 | 0.038 |
|  | acetylene degradation | 2.23E-05 | 0.62 | 0.537 | 0.035 | 0.491 | 0.062 | 0.377 | 0.062 |
|  | photorespiration | 1.10E-05 | 0.68 | 0.033 | 0.015 | 0.031 | 0.011 | 0.000 | 0.000 |
|  | L-lysine biosynthesis III | 9.61E-06 | 0.66 | 0.996 | 0.021 | 0.972 | 0.018 | 0.923 | 0.027 |
|  | biotin biosynthesis II | 2.23E-05 | 0.62 | 0.013 | 0.007 | 0.011 | 0.005 | 0.000 | 0.000 |
|  | L-lysine biosynthesis VI | 1.49E-05 | 0.61 | 0.989 | 0.027 | 0.958 | 0.018 | 0.916 | 0.027 |
|  | ubiquinol-7 biosynthesis (prokaryotic) | 1.32E-05 | 0.64 | 0.015 | 0.007 | 0.012 | 0.005 | 0.001 | 0.001 |
|  | ubiquinol-9 biosynthesis (prokaryotic) | 1.97E-05 | 0.64 | 0.015 | 0.007 | 0.012 | 0.005 | 0.001 | 0.001 |
|  | ubiquinol-10 biosynthesis (prokaryotic) | 1.76E-05 | 0.64 | 0.015 | 0.007 | 0.012 | 0.005 | 0.001 | 0.001 |
|  | superpathway of sulfolactate degradation | 1.35E-05 | 0.62 | 0.013 | 0.007 | 0.011 | 0.005 | 0.000 | 0.000 |
|  | ubiquinol-8 biosynthesis (prokaryotic) | 1.44E-05 | 0.64 | 0.015 | 0.007 | 0.012 | 0.005 | 0.001 | 0.001 |
|  | D-fructuronate degradation | 1.32E-04 | 0.51 | 0.697 | 0.057 | 0.648 | 0.054 | 0.552 | 0.070 |
|  | superpathway of purine deoxyribonucleosides degradation | 1.00E-05 | 0.71 | 0.582 | 0.030 | 0.541 | 0.052 | 0.424 | 0.049 |
|  | superpathway of pyrimidine deoxyribonucleosides degradation | 1.14E-05 | 0.75 | 0.602 | 0.033 | 0.585 | 0.039 | 0.460 | 0.040 |
|  | superpathway of ubiquinol-8 biosynthesis (prokaryotic) | 1.58E-05 | 0.64 | 0.014 | 0.006 | 0.012 | 0.005 | 0.001 | 0.001 |
| IL-4Rα^-/-^ | glycogen degradation I (bacterial) | 1.66E-04 | 0.51 | 1.047 | 0.055 | 1.061 | 0.051 | 0.946 | 0.048 |
|  | succinate fermentation to butanoate | 1.17E-05 | 0.61 | 0.007 | 0.002 | 0.035 | 0.013 | 0.032 | 0.012 |
|  | starch degradation V | 1.61E-04 | 0.53 | 0.988 | 0.051 | 1.014 | 0.051 | 0.895 | 0.044 |
|  | nitrate reduction VI (assimilatory) | 1.92E-04 | 0.68 | 0.105 | 0.022 | 0.215 | 0.050 | 0.116 | 0.020 |
